# Association of non-conventional lipid-inflammatory parameters with incident hypertension risk: a prospective cohort study

**DOI:** 10.7189/jogh.16.04237

**Published:** 2026-08-07

**Authors:** Mengjie Zhao, Miaoran Wang, Zirong Li, Yufei Wu, Fang Lu, Qiuyan Li

**Affiliations:** 1 Xiyuan Hospital, China Academy of Chinese Medical Sciences, Beijing, China; 2 Graduate School, Beijing University of Chinese Medicine, Beijing, China

**Keywords:** non-conventional lipid parameters, hs-CRP, hypertension, cohort, CHARLS

## Abstract

**Background:**

Emerging research has associated non-conventional lipid indices with hypertension risk; however, the joint contribution of these lipid metrics and inflammatory biomarkers to hypertension susceptibility remains unclear. Using a nationally representative cohort, we aimed to examine the independent associations of non-conventional lipid indices and their inflammatory composites with incident hypertension.

**Methods:**

We included 6891 participants from the China Health and Retirement Longitudinal Study (2011–2020). We used multivariable Cox proportional hazards regression to assess the association between baseline and cumulative non-conventional lipid-inflammatory parameters and incident hypertension. We used restricted cubic spline modelling to characterise dose-response relationships. We evaluated receiver operating characteristic curves, net reclassification improvement, and integrated discrimination improvement as secondary conditional analyses.

**Results:**

Over a median follow-up of 7.79 years, 2532 incident hypertension cases occurred. Higher tertiles of non-conventional lipid indices were associated with progressively increased hypertension risk. In the primary model, elevated baseline and cumulative non-conventional lipid metrics were associated with a higher risk of hypertension across all composite indices. The lipoprotein combine index-C-reactive protein showed the largest effect size (hazard ratio = 1.59; 95% confidence interval = 1.44–1.75). Composite indices demonstrated modest incremental discrimination over individual measures (area under the curve range = 0.55–0.58).

**Conclusions:**

Non-conventional lipid indices are independently associated with incident hypertension among middle-aged and older Chinese adults. Composite lipid-inflammatory markers provide modest incremental prognostic information beyond conventional lipids, though their standalone discriminatory performance is weak.

According to the most recent report from the World Health Organization, the global prevalence of hypertension in adults increased 2-fold, rising from 650 million to 1.3 billion over the 1990–2019 period [1]. Current forecasts suggest that the global prevalence of clinically diagnosed hypertension is projected to reach 1.56 billion by 2025 [2]. Hypertension stands as a major global risk factor for mortality and disability; in 2019, this condition was responsible for 10.8 million fatalities, with most of these deaths linked to cardiovascular diseases (CVDs) [1,3]. In China, the prevalence of hypertension increased from 18.8% in 2002 to 27.5% by 2018 [4].

Dyslipidaemia can elevate blood pressure through multiple pathways, including inducing endothelial dysfunction, exacerbating arterial stiffness, and inhibiting nitric oxide synthesis [5]. Studies have shown that levels of four conventional lipid markers deteriorate with increasing hypertension grades, among which total cholesterol (TC) and high-density lipoprotein cholesterol (HDL-C) exhibit the highest correlations with blood pressure [6]. Elevated levels of low-density lipoprotein cholesterol (LDL-C) and triglycerides (TGs), together with reduced HDL-C, are major risk factors for CVDs, with age and gender modulating lipid profiles in hypertensive patients [7]. As lipid research advances, non-conventional lipid indices have increasingly been utilised to investigate disease associations. Wang *et al.* demonstrated that elevated non-conventional lipid indices (*e.g.* TC/HDL-C, TG/HDL-C, LDL-C/HDL-C ratios, and non-HDL-C) are closely linked to deterioration in renal function among Chinese individuals with H-type hypertension [8] and further exhibit associations with the progression of stroke, prediabetes, and non-alcoholic hepatic steatosis [9–11]. Additional investigations have demonstrated that indicators such as remnant cholesterol (RC) and atherogenic index of plasma (AIP) are also associated with hypertension [12,13]. However, current assessments of these parameters rely predominantly on single cross-sectional measurements, with limited attention to their temporal variability and long-term implications. Dynamic assessment may provide more clinically valuable prognostic information, yet longitudinal studies investigating these changes remain scarce.

Lipid metabolism and inflammation are closely interconnected and mutually regulated [14]. LDL can activate the NLRP3 inflammasome *via* cholesterol crystals, trigger the release of interleukin-1β, disrupt T cell subset homeostasis, and exacerbate inflammation in atherosclerotic plaques [15]. Postprandial very-low-density lipoprotein remnants can act synergistically with tumour necrosis factor-α to enhance endothelial inflammation, while free fatty acids released during very-low-density lipoprotein TG hydrolysis can damage vascular endothelium, trigger local inflammation, and further accelerate the inflammatory process [16,17]. C-reactive protein (CRP) has been independently associated with hypertension and is recognised as a prognostic marker for this condition. It can elevate blood pressure through mechanisms such as activating inflammatory cells, inhibiting nitric oxide synthesis, impairing endothelial function, and promoting vascular smooth muscle cell proliferation [18,19]. Furthermore, CRP levels can predict long-term hypertension risk even in individuals with normal baseline blood pressure [20]. To date, longitudinal investigations integrating non-conventional lipid indices and high-sensitivity CRP (hs-CRP) remain limited, and the relationships between cumulative non-conventional lipid metrics, lipid-inflammatory composite parameters, and hypertension have yet to be fully clarified.

We hypothesised that non-conventional lipid-inflammatory composite markers are independently associated with incident hypertension among middle-aged and older Chinese adults, with long-term cumulative exposure exhibiting a stronger association than single-point measurements. We aimed to evaluate this hypothesis through a pre-specified three-tier framework: primary Cox regression analyses of baseline and cumulative exposure, dose-response characterisation, and conditional evaluation of incremental prognostic utility.

## METHODS

### Study design and participants

We performed secondary data analysis on the China Health and Retirement Longitudinal Study (CHARLS), a nationally representative, comprehensive longitudinal cohort study. We adhered to the Journal of Global Health's Guidelines for Reporting Analyses of Big Data Repositories Open to the Public (Table S1 in the **Online Supplementary Document**) [21]. The CHARLS employed a multistage stratified probability sampling to recruit eligible study subjects from urban and rural regions spanning 150 counties (districts) across 28 Chinese provinces, enrolling 17 708 individuals aged ≥45 years from 10 257 households at baseline [22]. Information on participants’ sociodemographic profiles, lifestyle behaviours, and health status was gathered *via* validated standardised questionnaires [23]. CHARLS initiated its first baseline assessment in 2011; by the time of this study’s implementation, five follow-up waves had been conducted, and four waves of follow-up data had been made available covering 2013, 2015, 2018, and 2020.

We excluded participants aged <45 years (n = 648), those with missing laboratory data on TC, TG, HDL-C, LDL-C, and hs-CRP (n = 5791), those with incomplete baseline diagnostic information for hypertension (n = 22), participants with confirmed hypertension at baseline (n = 3058), and those with missing hypertension diagnosis data during follow-up (n = 1298). Ultimately, we excluded 10 817 participants and included a total of 6891 eligible participants in our analysis (Figure S1 in the **Online Supplementary Document**).

### Data collection

We derived a comprehensive set of covariates from annual CHARLS questionnaire data, encompassing sociodemographic variables (age, gender, ethnicity, residential location, educational level, marital status, disability status, employment status, medical insurance coverage, smoking, and drinking behaviours), anthropometric measures (height, body weight, and waist circumference), medical history (diabetes, heart disease, stroke, and kidney disease), medication usage (hypoglycaemic and lipid-lowering agents), life satisfaction, sleep duration, and blood biochemical parameters. We generated a directed acyclic graph to illustrate the core interrelationships between non-conventional lipid indices, hs-CRP, hypertension, and all study covariates (Figure S2, Table S2 in the **Online Supplementary Document**).

Trained medical personnel from the Chinese Center for Disease Control and Prevention obtained the fasting venous blood specimens. They followed standardised operational protocols and transported these specimens to the central laboratory for biochemical analysis [24]. They quantified TC, TG, HDL-C, and LDL-C *via* an enzymatic colourimetric assay and assessed hs-CRP concentrations using an immunoturbidimetric method.

### Exposure assessment

We derived non-conventional lipid indices and lipid-inflammatory composite markers *via* the following formulas [25–31]:

− Non-HDL-C = TC − HDL-C
− Atherogenic coefficient (AC) = non-HDL-C / HDL-C
− AIP = lg(TG / HDL-C)
− Castelli risk index (CRI)-I = TC / HDL-C
− CRI-II = LDL-C / HDL-C
− Lipoprotein combine index (LCI) = (TC × TG × LDL-C) / HDL-C
− RC = TC − HDL-C − LDL-C
− RC/HDL-C ratio = RC / HDL-C
− We estimated cumulative exposure levels from 2012 to 2015 *via* the time-weighted average method: cumulative non-conventional lipid metric = ((parameter value in 2012 + parameter value in 2015) / 2) × (2015 − 2012)
− We generated lipid-inflammatory metrics by combining lipid parameters with hs-CRP: non-conventional lipid-inflammatory metric = non-conventional lipid metric (mg/dL) × hs-CRP (mg/L) / 10.

The CHARLS baseline survey was initiated in 2011, with fieldwork and blood sample collection extending through early 2012. In the cumulative exposure formula, the baseline biomarker value is abbreviated as the ‘2012’ measurement; this corresponds to the baseline (2011–2012) assessment. We used these two biomarker time points (2011–2012 and 2015) to calculate cumulative exposure because complete lipid panel and hs-CRP data were not available for the full cohort in the 2013 assessment. We used the time-weighted average of measurements separated by approximately three years to estimate cumulative metabolic burden, consistent with prior approaches in CHARLS-based cumulative exposure studies [9].

Under a landmark cohort framework, cumulative exposure indices were treated as fixed baseline exposures ascertained at the 2015 landmark. Only participants free of hypertension at the 2015 landmark were included in the cumulative exposure analysis, with follow-up for incident hypertension extending through 2020. This design ensures that exposure accumulation was completed before the start of the outcome observation window.

### Outcome

The primary endpoint was incident hypertension. At each follow-up wave (2013, 2015, 2018, and 2020), incident hypertension was defined as a self-reported physician diagnosis of hypertension. To ensure consistent ascertainment criteria across all waves – and in acknowledgement that standardised blood pressure measurements were unavailable at waves 4 and 5 – we did not incorporate measured blood pressure values into the outcome definition. For participants who reported a physician diagnosis, we assigned the self-reported year of first diagnosis as the event date, provided this date was after the baseline assessment. If the year of diagnosis was not reported, we used the interview date of the follow-up wave at which the case was first identified as the event date.

### Data analysis

#### Statistical analysis

We reported non-normally distributed continuous variables as median (interquartile range), performing intergroup comparisons using the Kruskal-Wallis test. We summarised categorical variables as frequency (percentage), assessing intergroup differences using the χ^2^ test.

#### Missing data and selection bias assessment

We imputed missing covariate data using the missForest algorithm in *R*, which applies a random forest approach to multiple imputation (Table S3 in the **Online Supplementary Document**). The imputation model included all variables from the primary Cox model as predictors. A total of five imputed data sets were generated. To verify that this number was adequate, we computed the fraction of missing information for key regression coefficients; all values were <0.3, indicating sufficient statistical efficiency. We assessed model convergence *via* out-of-bag imputation error across iterations, which stabilised for all variables. To evaluate potential selection bias arising from the exclusion of 10 817 participants, we compared baseline demographic, socioeconomic, and health characteristics between the analytical cohort (n = 6891) and the excluded subset using standardised mean differences (SMD); values <0.10 were considered an acceptable balance.

#### Primary analysis

We constructed two sequential Cox proportional hazards regression models. Model 1 adjusted for age and sex. Model 2 (the primary model) further adjusted for ethnicity, marital status, educational level, residential location, employment status, medical insurance, smoking and drinking history, sleep duration, and life satisfaction. We identified these covariates as the minimal sufficient adjustment set from a directed acyclic graph constructed *a priori* to minimise overadjustment and collider bias (Figure S2 in the **Online Supplementary Document**). We applied the Benjamini-Hochberg false discovery rate procedure across the eight non-conventional lipid-inflammatory indices to control for multiplicity. To characterise the shape of the exposure-response relationship, we performed multivariable-adjusted restricted cubic spline analysis to test linearity assumptions.

#### Secondary conditional analysis

We assessed incremental prognostic utility using receiver operating characteristic curves, with the area under the curve (AUC) as the quantitative metric. We employed net reclassification improvement (NRI) and integrated discrimination improvement (IDI) to evaluate whether composite indices added information beyond conventional lipids. We performed these analyses only for indices with a statistically significant association in the primary model and treated them as hypothesis-supportive rather than independent hypothesis tests.

#### Exploratory analyses

We performed subgroup analyses stratified by age, sex, educational level, employment status, residential location, smoking and drinking history, life satisfaction, and baseline medication use (antidiabetic and lipid-lowering drugs) to assess potential effect modification. We conducted a series of pre-specified sensitivity analyses to examine the stability of the primary hazard ratios (HRs): one excluding individuals with baseline CVD; one removing incident hypertension cases ascertained at the second follow-up wave to mitigate reverse causality; one expanding the model to include additional haematological markers (*i.e.* fasting blood glucose, white blood cell count, platelet count, blood urea nitrogen, serum creatinine, glycated haemoglobin A1c, and uric acid); one repeating analyses using the complete-case (non-imputed) data set; Cox regression with time-updated covariates; and inverse probability weighting to approximate the covariate distribution of the full CHARLS baseline sample (selection model included age, sex, ethnicity, marital status, education, residential location, employment, insurance, smoking, drinking, sleep duration, and life satisfaction). Additionally, we computed E-values to quantify the minimum strength of association an unmeasured confounder would need to fully explain the observed associations. These checks served as robustness validations for the primary analysis model and did not constitute independent analytical aims.

We stratified the participants into tertiles based on non-conventional lipid-inflammatory index levels. We used Kaplan-Meier survival curves with log-rank tests to examine cumulative incidence. We evaluated multicollinearity *via* the variance inflation factor; all variables had values of <5 (Figures S3 and S4 in the **Online Supplementary Document**).

We used *R*, version 4.5.1 (R Core Team, Vienna, Austria) for all analyses. A two-tailed *P*-value <0.05 was considered statistically significant.

## RESULTS

### Baseline characteristics

We included 6891 participants in the analysis. Over a median follow-up of 7.79 years, 2532 incident hypertension cases were identified (**Table 1**). The cohort had a median age of 57 years, and 53.46% were female. Participants who developed incident hypertension tended to be older, had lower educational attainment, were more likely to reside in rural regions, and had higher rates of disability, alcohol consumption, and obesity (*P* < 0.001). This subgroup also had a higher prevalence of comorbidities, including hyperlipidaemia, diabetes mellitus, heart disease, and kidney disease (*P* < 0.001).

**Table 1:** Baseline characteristics of the study population stratified by new-onset hypertension

	Total (n = 6891)	Non-hypertension (n = 4359)	Hypertension (n = 2532)	*P-*value
Age in years*	57.00 (50.00–63.00)	56.00 (49.00–61.00)	58.00 (52.00–65.00)	<0.001
**Age†**				<0.001
<60	4338 (62.95)	2948 (67.63)	1390 (54.9)	
≥60	2553 (37.05)	1411 (32.37)	1142 (45.1)	
**Gender†**				0.236
Female	3684 (53.46)	2354 (54)	1330 (52.53)	
Male	3207 (46.54)	2005 (46)	1202 (47.47)	
**Nation†**				0.002
Other	486 (7.05)	276 (6.33)	210 (8.29)	
Han ethnicity	6405 (92.95)	4083 (93.67)	2322 (91.71)	
**Education†**				<0.001
Elementary school and below	4675 (67.84)	2850 (65.38)	1825 (72.08)	
Above elementary school	2216 (32.16)	1509 (34.62)	707 (27.92)	
**Marital status†**				<0.001
Married	6224 (90.32)	3999 (91.74)	2225 (87.88)	
Other	667 (9.68)	360 (8.26)	307 (12.12)	
**Residence†**				0.008
City	2316 (33.61)	1515 (34.76)	801 (31.64)	
Rural	4575 (66.39)	2884 (65.24)	1731 (68.36)	
**Work†**				<0.001
No	1929 (27.99)	1138 (26.11)	791 (31.24)	
Yes	4962 (72.01)	3221 (73.89)	1741 (68.76)	
**Health insurance†**				0.503
No	370 (5.37)	228 (5.23)	142 (5.61)	
Yes	6521 (94.63)	4131 (94.77)	2390 (94.39)	
**Life satisfaction†**				0.264
Satisfied	5854 (84.95)	3719 (85.32)	2135 (84.32)	
Unsatisfied	1037 (15.05)	640 (14.68)	397 (15.68)	
**Disability†**	1077 (15.63)	633 (14.52)	444 (17.54)	<0.001
**Waist (cm), x (SD)**	82.97 (12.09)	81.60 (11.82)	85.32 (12.17)	<0.001
**BMI (kg/m^2^), x (SD)**	23.13 (3.69)	22.75 (3.59)	23.79 (3.76)	<0.001
**BMI†**				<0.001
<24	4366 (63.36)	2974 (68.23)	1392 (54.98)	
≥24	2525 (36.64)	1385 (31.77)	1140 (45.02)	
**Smoke†**				0.445
No	4229 (61.37)	2690 (61.71)	1539 (60.78)	
Yes	2662 (38.63)	1669 (38.29)	993 (39.22)	
**Drink†**				0.027
No	4213 (61.14)	2708 (62.12)	1505 (59.44)	
Yes	2678 (38.86)	1651 (37.88)	1027 (40.56)	
Sleep duration, hours*	6.50 (5.00–8.00)	7.00 (5.00–8.00)	6.00 (5.00–8.00)	0.056
**Hyperlipidaemia†**	418 (6.07)	234 (5.37)	184 (7.27)	0.001
**DM†**	261 (3.79)	133 (3.05)	128 (5.06)	<0.001
**HD†**	531 (7.71)	286 (6.56)	245 (9.68)	<0.001
**Stroke†**	77 (1.12)	42 (0.96)	35 (1.38)	0.111
**Kidney disease†**	374 (5.43)	216 (4.96)	158 (6.24)	0.023
**Antidiabetic drugs†**	160 (2.32)	78 (1.79)	82 (3.24)	<0.001
**Lipid-lowering drug†**	179 (2.60)	94 (2.16)	85 (3.36)	0.003
TC (mg/dL)*	189.40 (166.60–213.80)	187.10 (165.50–211.30)	192.90 (168.90–217.70)	<0.001
TG (mg/dL)*	100.90 (73.46–148.70)	97.35 (70.80–140.30)	109.70 (77.88–163.10)	<0.001
HDL-C (mg/dL)*	50.26 (40.98–60.70)	51.03 (41.75–61.08)	49.10 (39.82–59.15)	<0.001
LDL-C (mg/dL)*	114.00 (93.17–135.70)	112.90 (92.40–134.20)	115.60 (93.94–139.20)	<0.001
CRP (mg/L)*	0.91 (0.51–1.89)	0.85 (0.49–1.74)	1.03 (0.56–2.15)	<0.001
AC*	2.73 (2.02–3.65)	2.63 (1.98–3.53)	2.94 (2.15–3.84)	<0.001
AIP*	0.30 (0.10–0.53)	0.27 (0.09–0.50)	0.35 (0.14–0.58)	<0.001
CRI-I*	3.73 (3.02–4.65)	3.63 (2.98–4.53)	3.94 (3.15–4.84)	<0.001
CRI-II*	2.28 (1.74–2.92)	2.20 (1.70–2.85)	2.38 (1.80–3.02)	<0.001
LCI*	43 860.00 (24 330.00–80 980.00)	40 480.00 (22 720.00–73 840.00)	50 880.00 (27 530.00–93 610.00)	<0.001
Non-HDL-C*	136.90 (114.00–162.00)	134.50 (112.50–158.50)	141.50 (117.90–166.20)	<0.001
RC*	18.94 (10.82–30.54)	17.78 (10.44–28.61)	20.88 (12.37–34.12)	<0.001
RC/HDL-C*	0.37 (0.19–0.69)	0.34 (0.18–0.63)	0.42 (0.21–0.78)	<0.001

AC – atherogenic coefficient, AIP – atherogenic index of plasma, BMI – body mass index, CRI – Castelli risk index, CRP – C-reactive protein, DM – diabetes mellitus, HD – heart disease, HDL-C – high-density lipoprotein cholesterol, IQR – interquartile range, LCI – lipoprotein combine index, LDL-C – low-density lipoprotein cholesterol, MD – median, RC – remnant cholesterol, TC – total cholesterol, TG – triglycerides

*Values are presented as MD (IQR)

†Values presented as n (%).

We also compared baseline characteristics between the analytical cohort (n = 6891) and excluded participants (n = 10 817) (Table S4 in the **Online Supplementary Document**). Because participants with prevalent hypertension at baseline were excluded by design, the analytical cohort differed from the excluded subset on several expected dimensions. Specifically, the analytical cohort was younger (57.6 *vs.* 59.7 years; SMD = 0.213), had lower cardiometabolic comorbidity burden (*e.g.* heart disease:  8% *vs.* 15%; SMD = 0.23), and was more likely to be employed (72% *vs.* 57%; SMD = 0.31). These differences are consistent with the intentional restriction to participants free of baseline hypertension, which represents the appropriate denominator for estimating incident disease risk.

### Association between non-conventional lipid indices and risk of incident hypertension

After full covariate adjustment, individuals in the highest tertile of non-conventional lipid indices had a higher risk of incident hypertension compared with those in the lowest tertile. Among these parameters, LCI showed the largest effect size (HR = 1.61; 95% confidence interval (CI) = 1.47–1.78). Kaplan-Meier curves showed patterns consistent with these findings, with higher tertiles of non-conventional lipid indices associated with a stepwise increase in incident hypertension risk (*P* < 0.0001); the magnitude of these associations was greater than that observed for conventional lipid markers. Multivariable-adjusted restricted cubic spline analysis indicated a linear, positive association between AIP, CRI-II, and non-HDL-C and incident hypertension risk (*P* for nonlinearity >0.05) (Figures S5 and S6, Table S5 in the **Online Supplementary Document**).

### Association of cumulative non-conventional lipid indices with incident hypertension risk

**Figure 1:**
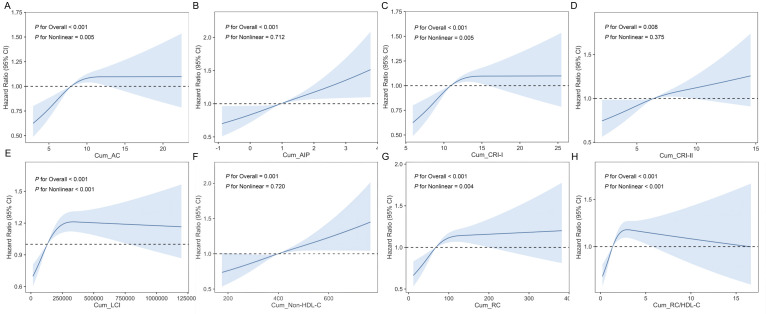
Dose-response relationship between cumulative non-conventional lipid parameters and hypertension risk. **Panel A.** Cum_AC. **Panel B.** Cum_AIP. **Panel C.** Cum_CRI-I. **Panel D.** Cum_CRI-II. **Panel E.** Cum_LCI. **Panel F.** Cum_Non-HDL-C. **Panel G.** Cum_RC. **Panel H.** Cum_RC/HDL-C. CI – confidence interval, Cum_AC – cumulative atherogenic coefficient, Cum_AIP – cumulative atherogenic index of plasma, Cum_CRI-I – cumulative Castelli risk index-I, Cum_CRI-II – cumulative Castelli risk index-II, Cum_LCI – cumulative lipoprotein combine index, Cum_Non-HDL-C – cumulative non-high-density lipoprotein cholesterol, Cum_RC – cumulative remnant cholesterol, Cum_RC/HDL-C – cumulative remnant cholesterol/high density lipoprotein cholesterol.

After stratification by tertiles of cumulative parameters in the primary model, higher levels of all cumulative non-conventional lipid indices were independently associated with incident hypertension risk. Among these, cumulative LCI had the largest effect size (HR = 1.70, 95% CI = 1.46–1.97), followed by cumulative AIP (HR = 1.69; 95% CI = 1.46–1.95) (**Table 2**). Kaplan-Meier curves were consistent with these findings, with elevated cumulative non-conventional lipid indices associated with a progressive increase in incident hypertension risk (*P* < 0.0001). The magnitude of these associations was greater than that for conventional lipid markers (Figure S7, Table S5 in the **Online Supplementary Document**). Multivariable-adjusted restricted cubic spline analysis indicated linear, positive associations for cumulative AIP, CRI-II, and non-HDL-C (*P* for nonlinearity >0.05). In contrast, cumulative AC, CRI-I, LCI, RC, and RC/HDL-C showed nonlinear associations (*P* for nonlinearity <0.05) (**Figure 1**, Panels A–H). These findings suggest that cumulative non-conventional lipid indices are independently associated with incident hypertension risk over extended follow-up.

**Table 2:** Associations between cumulative non-conventional lipid parameters and hypertension

		Crude model		Model 1*		Model 2†		
	**n (%)**	**HR (95% CI)**	***P*-value**	**HR (95% CI)**	***P*-value**	**HR (95% CI)**	***P*-value**	**Adjusted *P*-value**
**Cumulative AC**								
T1	303 (21.7)	ref		ref		ref		
T2	394 (28.2)	1.36 (1.17–1.58)	<0.001	1.37 (1.18–1.59)	<0.001	1.41 (1.21–1.64)	<0.001	<0.001
T3	446 (32)	1.56 (1.35–1.81)	<0.001	1.59 (1.37–1.84)	<0.001	1.67 (1.44–1.93)	<0.001	<0.001
*P* for trend			<0.001		<0.001		<0.001	<0.001
**Cumulative AIP**								
T1	315 (22.6)	ref		ref		ref		
T2	375 (26.9)	1.23 (1.06–1.42)	0.008	1.25 (1.08–1.45)	0.004	1.28 (1.10–1.49)	0.001	0.001
T3	453 (32.5)	1.52 (1.32–1.75)	<0.001	1.61 (1.39–1.86)	<0.001	1.69 (1.46–1.95)	<0.001	<0.001
*P* for trend			<0.001		<0.001		<0.001	<0.001
**Cumulative CRI-I**								
T1	303 (21.7)	ref		ref		ref		
T2	394 (28.2)	1.36 (1.17–1.58)	<0.001	1.37 (1.18–1.59)	<0.001	1.41 (1.21–1.64)	<0.001	<0.001
T3	446 (32)	1.56 (1.35–1.81)	<0.001	1.59 (1.37–1.84)	<0.001	1.67 (1.44–1.93)	<0.001	<0.001
*P* for trend			<0.001		<0.001		<0.001	<0.001
**Cumulative CRI-II**								
T1	320 (23)	ref		ref		ref		
T2	387 (27.7)	1.25 (1.08–1.45)	0.003	1.27 (1.10–1.48)	0.001	1.30 (1.12–1.50)	0.001	0.001
T3	436 (31.3)	1.43 (1.24–1.65)	<0.001	1.43 (1.23–1.65)	<0.001	1.48 (1.28–1.71)	<0.001	<0.001
*P* for trend			<0.001		<0.001		<0.001	<0.001
**Cumulative LCI**								
T1	303 (21.7)	ref		ref		ref		
T2	391 (28)	1.35 (1.17–1.57)	<0.001	1.37 (1.18–1.60)	<0.001	1.40 (1.21–1.63)	<0.001	<0.001
T3	449 (32.2)	1.57 (1.36–1.82)	<0.001	1.63 (1.41–1.89)	<0.001	1.70 (1.46–1.97)	<0.001	<0.001
*P* for trend			<0.001		<0.001		<0.001	<0.001
**Cumulative Non-HDL-C**								
T1	331 (23.7)	ref		ref		ref		
T2	362 (25.9)	1.11 (0.95–1.28)	0.183	1.10 (0.95–1.28)	0.216	1.12 (0.96–1.30)	0.150	0.150
T3	450 (32.3)	1.42 (1.23–1.64)	<0.001	1.43 (1.23–1.65)	<0.001	1.46 (1.26–1.69)	<0.001	<0.001
*P* for trend			<0.001		<0.001		<0.001	<0.001
**Cumulative RC**								
T1	325 (23.3)	ref		ref		ref		
T2	358 (25.7)	1.12 (0.96–1.30)	0.144	1.12 (0.96–1.30)	0.136	1.14 (0.98–1.32)	0.094	0.094
T3	460 (33)	1.49 (1.29–1.72)	<0.001	1.56 (1.35–1.80)	<0.001	1.60 (1.39–1.85)	<0.001	<0.001
*P* for trend			<0.001		<0.001		<0.001	<0.001
**Cumulative RC/HDL-C**								
T1	319 (22.9)	ref		ref		ref		
T2	366 (26.2)	1.18 (1.01–1.37)	0.034	1.20 (1.03–1.39)	0.019	1.22 (1.05–1.42)	0.010	0.010
T3	458 (32.9)	1.52 (1.32–1.75)	<0.001	1.59 (1.38–1.83)	<0.001	1.65 (1.43–1.91)	<0.001	<0.001
*P* for trend			<0.001		<0.001		<0.001	<0.001

AC – atherogenic coefficient, AIP – atherogenic index of plasma, CI – confidence interval, CRI – Castelli risk index, HDL-C – high-density lipoprotein cholesterol, HR – hazard ratio, LCI – lipoprotein combine index, RC – remnant cholesterol, ref – reference, T1 – first tertile, T2 – second tertile, T3 – third tertile

*Adjusted for age and gender.

**†**Adjusted for age, gender, ethnicity, marital status, educational level, residential location, employment status, medical insurance coverage, smoking and drinking history, sleep duration, and life satisfaction.

### Association of non-conventional lipid-inflammatory indices with incident hypertension risk

**Figure 2:**
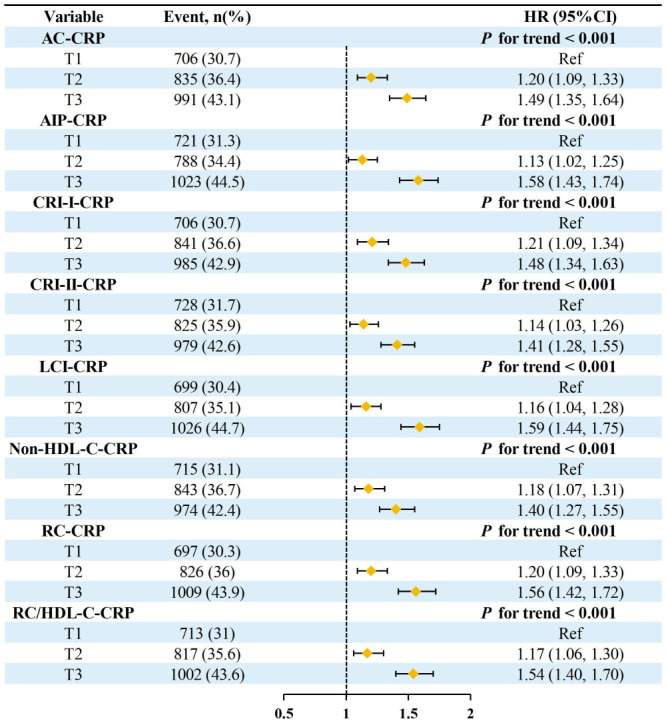
Associations of non-conventional lipid and lipid-inflammatory parameters with hypertension. AC – atherogenic coefficient, AIP – atherogenic index of plasma, CI – confidence interval, CRI – Castelli risk index, CRP – C-reactive protein, HDL-C – high-density lipoprotein cholesterol, HR – hazard ratio, LCI – lipoprotein combine index, RC – remnant cholesterol, T1 – first tertile, T2 – second tertile, T3 – third tertile.

In the primary model, after stratifying participants into tertiles, all tested parameters were positively associated with incident hypertension risk; compared with the lowest tertile, the highest tertile was associated with a higher risk. Among these, LCI-CRP had the largest effect sizes (HR = 1.59; 95% CI = 1.44–1.75), with estimates consistent across subgroup and sensitivity analyses (**Figure 2**).

Analysis of cumulative parameter levels showed that cumulative LCI-CRP had the largest effect size (HR = 1.84; 95% CI = 1.59–2.13), followed by cumulative AIP-CRP (HR = 1.79; 95% CI = 1.54–2.07) (**Table 3**). Kaplan-Meier curves were consistent with these findings, with elevated non-conventional lipid-inflammatory indices associated with a progressive increase in incident hypertension risk (*P* < 0.0001) (Figures S8 and S9 in the **Online Supplementary Document**).

**Table 3:** Associations between cumulative non-conventional lipid-inflammatory parameters and hypertension

		Crude model		Model 1*		Model 2†		
	**n (%)**	**HR (95% CI)**	***P*-value**	**HR (95% CI)**	***P*-value**	**HR (95% CI)**	***P*-value**	**Adjusted *P*-value**
**Cumulative AC-CRP**								
T1	294 (21.1)	ref		ref		ref		
T2	393 (28.2)	1.39 (1.20–1.62)	<0.001	1.38 (1.18–1.60)	<0.001	1.39 (1.19–1.62)	<0.001	<0.001
T3	456 (32.7)	1.66 (1.43–1.92)	<0.001	1.64 (1.41–1.90)	<0.001	1.67 (1.44–1.94)	<0.001	<0.001
*P* for trend			<0.001		<0.001		<0.001	<0.001
**Cumulative AIP-CRP**								
T1	298 (21.4)	ref		ref		ref		
T2	375 (26.9)	1.30 (1.12–1.51)	0.001	1.30 (1.12–1.52)	0.001	1.32 (1.14–1.54)	<0.001	<0.001
T3	470 (33.7)	1.70 (1.47–1.97)	<0.001	1.73 (1.49–2.00)	<0.001	1.79 (1.54–2.07)	<0.001	<0.001
*P* for trend			<0.001		<0.001		<0.001	<0.001
**Cumulative CRI-I-CRP**								
T1	287 (20.6)	ref		ref		ref		
T2	402 (28.8)	1.47 (1.27–1.71)	<0.001	1.45 (1.25–1.69)	<0.001	1.46 (1.25–1.70)	<0.001	<0.001
T3	454 (32.5)	1.70 (1.46–1.97)	<0.001	1.66 (1.43–1.93)	<0.001	1.69 (1.46–1.97)	<0.001	<0.001
*P* for trend			<0.001		<0.001		<0.001	<0.001
**Cumulative CRI-II-CRP**								
T1	292 (20.9)	ref		ref		ref		
T2	405 (29.1)	1.45 (1.25–1.69)	<0.001	1.43 (1.23–1.67)	<0.001	1.44 (1.24–1.67)	<0.001	<0.001
T3	446 (32)	1.63 (1.41–1.89)	<0.001	1.60 (1.38–1.85)	<0.001	1.63 (1.40–1.89)	<0.001	<0.001
*P* for trend			<0.001		<0.001		<0.001	<0.001
**Cumulative LCI-CRP**								
T1	289 (20.7)	ref		ref		ref		
T2	376 (27)	1.35 (1.15–1.57)	<0.001	1.32 (1.14–1.54)	<0.001	1.34 (1.15–1.56)	<0.001	<0.001
T3	478 (34.3)	1.79 (1.55–2.08)	<0.001	1.79 (1.55–2.08)	<0.001	1.84 (1.59–2.13)	<0.001	<0.001
*P* for trend			<0.001		<0.001		<0.001	<0.001
**Cumulative Non-HDL-C-CRP**								
T1	297 (21.3)	ref		ref		ref		
T2	393 (28.2)	1.37 (1.18–1.60)	<0.001	1.34 (1.15–1.56)	<0.001	1.35 (1.16–1.57)	<0.001	<0.001
T3	453 (32.5)	1.63 (1.41–1.89)	<0.001	1.59 (1.37–1.84)	<0.001	1.61 (1.39–1.87)	<0.001	<0.001
*P* for trend			<0.001		<0.001		<0.001	<0.001
**Cumulative RC-CRP**								
T1	298 (21.4)	ref		ref		ref		
T2	383 (27.5)	1.33 (1.15–1.55)	<0.001	1.32 (1.13–1.53)	<0.001	1.33 (1.14–1.55)	<0.001	<0.001
T3	462 (33.1)	1.66 (1.44–1.92)	<0.001	1.66 (1.43–1.92)	<0.001	1.69 (1.46–1.95)	<0.001	<0.001
*P* for trend			<0.001		<0.001		<0.001	<0.001
**Cumulative RC/HDL-C-CRP**								
T1	289 (20.7)	ref		ref		ref		
T2	392 (28.1)	1.43 (1.23–1.66)	<0.001	1.43 (1.23–1.66)	<0.001	1.45 (1.24–1.68)	<0.001	<0.001
T3	462 (33.1)	1.72 (1.49–2.00)	<0.001	1.72 (1.49–1.99)	<0.001	1.77 (1.52–2.05)	<0.001	<0.001
*P* for trend			<0.001		<0.001		<0.001	<0.001

AC – atherogenic coefficient, AIP – atherogenic index of plasma, CI – confidence interval, CRI – Castelli risk index, CRP – C-reactive protein, HDL-C – high-density lipoprotein cholesterol, HR – hazard ratio, LCI – lipoprotein combine index, RC – remnant cholesterol, ref – reference, T1 – first tertile, T2 – second tertile, T3 – third tertile

*Adjusted for age and gender.

**†**Adjusted for age, gender, ethnicity, marital status, educational level, residential location, employment status, medical insurance coverage, smoking and drinking history, sleep duration, and life satisfaction.

### Predictive efficacy of non-conventional lipid and lipid-inflammatory indices

**Figure 3:**
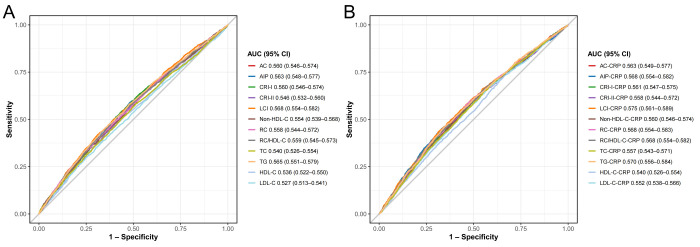
Predictive ability of lipid and lipid-inflammatory parameters for hypertension. **Panel A.** Non-traditional lipid parameters. **Panel B.** Non-traditional lipid-inflammation parameters. AC – atherogenic coefficient, AIP – atherogenic index of plasma, AUC – area under the curve, CI – confidence interval, CRI – Castelli risk index, CRP – C-reactive protein, HDL-C – high-density lipoprotein cholesterol, LCI – lipoprotein combine index, RC – remnant cholesterol.

Receiver operating characteristic curve analysis indicated that individual lipid parameters had weak to modest discriminatory performance for incident hypertension, with non-conventional lipid indices demonstrating marginally higher AUC values than conventional ones (**Figure 3**, Panels A and B; Table S6 in the **Online Supplementary Document**). The AUC values for conventional lipid indices ranged from 0.527 to 0.565, whereas those for non-conventional lipid indices ranged from 0.546 to 0.568; LCI had the highest AUC among individual indices (AUC = 0.568; 95% CI = 0.554–0.582). Adding hs-CRP to non-conventional lipid indices yielded modest incremental discrimination, with LCI-CRP showing the highest (AUC = 0.575; 95% CI = 0.561–0.589); similar patterns were observed for cumulative parameters (Figure S10, Table S7 in the **Online Supplementary Document**).

We used NRI and IDI to assess incremental reclassification utility (Tables S8 and S9 in the **Online Supplementary Document**). Adding non-conventional lipid markers to the traditional parameter-based model was associated with small increases in NRI and IDI (*P* < 0.001). The RC/HDL-C-CRP model had an NRI of 0.087 (95% CI = 0.066–0.114), and the cumulative RC/HDL-C-CRP model had an NRI of 0.127 (95% CI = 0.088–0.165). Subgroup analyses suggested that these predictive estimates varied by gender and age (Figures S11–14 in the **Online Supplementary Document**).

### Subgroup analysis

We performed subgroup analyses stratified by pre-specified factors, including age, sex, educational attainment, employment status, residential setting, smoking behaviour, alcohol intake, life satisfaction, antidiabetic drugs, and lipid-lowering drugs (Tables S10 and S11 in the **Online Supplementary Document**). Subgroup analysis of non-conventional lipid-inflammatory markers showed that no significant interaction was identified between each stratification factor and these markers (Tables S10 and S11 in the **Online Supplementary Document**).

### Sensitivity analysis

To examine the stability of the primary findings under alternative specifications, we conducted several sensitivity analyses. First, we excluded participants with baseline CVD (heart disease and stroke). Second, we excluded hypertension cases ascertained at the second follow-up wave to mitigate potential reverse causality. Third, we expanded the regression model to include additional haematological indicators (fasting blood glucose, white blood cell count, platelet count, blood urea nitrogen, serum creatinine, glycated haemoglobin A1c, and uric acid). Fourth, we repeated the analyses using the complete-case data set (n = 5497). Fifth, we performed Cox regression with time-updated covariates. Sixth, we applied inverse probability weighting to account for potential selection bias. The results across these sensitivity analyses were generally consistent with the primary findings (Tables S12–17 in the **Online Supplementary Document**). In addition, E-values for the primary lipid parameters ranged from 2.15 to 3.08 (Table S18 in the **Online Supplementary Document**), suggesting that the observed associations would require moderate-to-strong unmeasured confounding to be fully explained away.

## DISCUSSION

Using a large, nationally representative prospective cohort, we examined the association between eight non-conventional lipid indices and incident hypertension risk among Chinese middle-aged and older adults. Our findings indicate that non-conventional lipid indices were independently associated with modestly increased hypertension risk. The addition of hs-CRP to non-conventional lipid indices yielded a small improvement in risk reclassification, as reflected by NRI and IDI estimates of limited clinical magnitude. Furthermore, higher cumulative levels of lipid-inflammatory composite markers were associated with elevated hypertension risk over extended follow-up.

Conventional lipid markers, including LDL-C, HDL-C, and TG, serve as core indicators for CVD risk assessment. Studies have confirmed that reducing LDL-C can significantly reduce cardiovascular events, but 50% residual risk remains after reaching the target, necessitating the development of novel predictive indicators [32]. HDL-C is involved in reverse cholesterol transport and inflammation regulation, serving as a protective factor against CVD [33]; however, quantifying its concentration alone cannot fully assess its function [34]. A large-scale genetic study of more than one million participants identified novel lipid-associated gene loci, illuminating the tight link between blood pressure homeostasis and lipid metabolism [35]. Mendelian randomisation studies have confirmed that different lipid parameters exert a causal effect on blood pressure, among which the TG component of small-particle HDL has the most significant effect on increasing blood pressure [36]. TG can promote lipid exchange between lipoproteins, transforming HDL into a subtype rich in TG and depleted of cholesterol ester core, leading to an increased proportion of small-particle HDL and a reduced anti-atherosclerotic HDL subpopulation, thereby impairing HDL function [37]. Such small-particle HDL is a key factor in elevating blood pressure. In addition, dyslipidaemia damages vascular endothelium, disrupts the balance of endothelium-derived vasoactive factors, inhibits nitric oxide synthesis, and induces endothelial dysfunction and blood pressure disorders [38]. Endothelial injury further exacerbates the loss of vascular motor activity and contraction imbalance, forming a vicious cycle of elevated blood pressure [39]. Traditional lipid markers fail to reflect the interactions and balance between lipid components, which are crucial for CVD risk stratification. Over the past decade, non-conventional lipid indices have emerged as a prominent research focus. By integrating multiple lipid parameters, they can capture residual risks overlooked by traditional markers and enhance prognostic value. Studies have confirmed that RC [40], AIP [41], non-HDL-C [42], AC [43], and other parameters have good predictive value for hypertension. However, existing evidence is primarily derived from single cross-sectional assessments, overlooking temporal fluctuations in metabolic-inflammatory load and lacking longitudinal evaluations of cumulative impacts, which are susceptible to regression dilution bias and compromise the reliability of study conclusions. We constructed cumulative indicators using multi-time point data to accurately capture the synergistic interplay between chronic inflammation and metabolic dysfunction.

Our findings indicate that cumulative non-conventional lipid indices, which reflect long-term lipid metabolic burden, were independently associated with incident hypertension risk among middle-aged and older adults. In the fully adjusted multivariable model, the odds of hypertension in the top tertile of cumulative LCI were 70% higher than those in the bottom tertile (HR = 1.70; 95% CI = 1.46–1.97); cumulative AIP followed (HR = 1.69; 95% CI = 1.46–1.95). The LCI is a potential biomarker for metabolic syndrome-related diseases, widely used in CVD risk prediction [44]. The AIP is linked to hypertension – a Chinese cohort study showed that each one-unit elevation in AIP was linked to an 84% greater risk of incident hypertension [13]. The association between non-HDL-C and hypertension risk is supported by prior evidence [6]; clinical guidelines acknowledge that non-HDL-C reflects the TC content of apolipoprotein B-containing lipoproteins and is associated with cardiovascular risk to an extent comparable to LDL-C [45]. The link between RC and incident hypertension has been corroborated by multiple studies [40,46], and a meta-analysis also verified that higher RC levels are associated with an elevated risk of hypertension [47]. RC can infiltrate the arterial endothelial layer and deposit, trigger foam cell generation and inflammatory reactions, impair endothelial function, increase vascular resistance [48,49], and regulate obesity-related HD risk [50]. An Iranian cohort study showed that high cumulative RC exposure and high variability were linked to a higher risk of incident hypertension [51], consistent with our results. Relative to single lipid markers, comprehensive non-conventional lipid markers like LCI, AIP, and AC can more holistically reflect lipid metabolism status, showing higher clinical utility in predicting CVD risk [52,53].

Dyslipidaemia is closely associated with inflammation and serves as a core driver of inflammatory progression. After subendothelial LDL is modified into modified LDL, it binds to endothelial receptors to activate the endothelium, up-regulate adhesion molecules and chemokines [54], and recruit monocytes to migrate to the intima and differentiate into macrophages [55]. Macrophages phagocytose modified LDL to transform into foam cells [56], which not only reflect lipid accumulation but also release pro-inflammatory factors to amplify the inflammatory cascade reaction [57]. Inflammation also up-regulates lipid uptake receptors and inhibits cholesterol efflux [58], exacerbating lipid deposition. Prior research has reported that elevated TG, TC, and LDL-C levels, as well as decreased HDL-C levels, are associated with inflammatory activation and higher serum CRP concentrations [59–61]. CRP acts as a pivotal factor mediating dyslipidaemia and atherosclerotic pathogenesis, and its mechanism likely involves the modulation of oxidative stress responses [62]. Building on this evidence, we constructed lipid-inflammatory composite indices and evaluated their incremental prognostic utility relative to individual lipid measures.

Longitudinal changes in non-conventional lipid-inflammatory composite indices were associated with subsequent hypertension risk in this observational cohort. While prior evidence suggests that elevated CRP may precede incident hypertension in older adults [20], the temporal relationship between dyslipidaemia, inflammation, and elevated blood pressure cannot be firmly established here. These findings are hypothesis-generating: integrated lipid-inflammatory markers may reflect cumulative pathophysiological processes, but whether they provide clinically meaningful early detection signals remains speculative and warrants further investigation.

The HRs we observed represent modest associations at the individual level. However, at the population level, the large size of the middle-aged and older adult population in China means that a 40–60% increase in relative risk may translate into a substantial population attributable fraction, particularly given the high prevalence of dyslipidaemia and subclinical inflammation in this demographic. We emphasise that this population-level perspective does not imply individual-level clinical decisiveness. Such lipid-inflammatory markers may serve only as supplementary components within multivariable risk assessment frameworks, rather than as standalone screening or prevention tools. Any application to clinical risk stratification or preventive resource allocation would require independent external validation, cost-effectiveness analyses, and demonstration of net benefit before implementation.

The AUC values for both conventional (AUC range = 0.53–0.57) and non-conventional (AUC range = 0.55–0.58) lipid indices indicated weak-to-modest discrimination, consistent with the known performance limitations of individual metabolic biomarkers for multifactorial chronic conditions. We did not seek to establish standalone predictive efficacy for any single index, but examined whether these markers provide statistically significant incremental information beyond conventional lipid measures. Cumulative RC/HDL-C-CRP composite achieved an NRI of 0.127 (95% CI = 0.088–0.165), indicating a modest but statistically significant improvement in risk reclassification. While this degree of reclassification is modest, it may offer incremental value for population-level risk assessment if applied at a sufficient scale. We acknowledge that the weak discrimination we observed precludes the use of non-conventional lipid-inflammatory indices as standalone screening tools. In settings where advanced risk stratification tools are unavailable, these indices might serve as low-cost supplementary data points within broader multivariable assessment frameworks. The incremental NRI of 0.127 suggests that integrating these markers could reclassify roughly one in eight individuals into an alternative risk tier; however, whether such reclassification translates into improved clinical outcomes is unknown. Any consideration of clinical implementation would require external validation, cost-effectiveness evaluation, and evidence of net benefit from randomised trials before guideline adoption.

### Strengths and limitations

This study has several methodological features worth noting. The prospective design and cumulative exposure metrics allowed temporal sequencing of lipid-inflammatory markers relative to incident hypertension. The nationally representative CHARLS cohort provides findings that may be generalisable to middle-aged and older Chinese adults, though extrapolation to other populations requires caution.

Several limitations should be acknowledged. First, despite comprehensive multivariable adjustment, residual confounding cannot be excluded; we quantified the potential impact of unmeasured confounders using E-values. Second, findings from this single Chinese cohort require replication in independent cohorts before broader generalisation. As with all observational studies, causal inference is precluded by the non-randomised design. Additionally, batch effects across CHARLS follow-up waves may have introduced systematic variation in lipid parameter measurements. Also, outcome ascertainment relied solely on self-reported hypertension diagnosis and medication use; undiagnosed hypertensive cases could not be identified. This non-differential outcome misclassification would likely bias HR estimates toward the null, suggesting that the true associations may be stronger than those reported. Future studies incorporating standardised blood pressure measurement at all follow-up waves are warranted. Moreover, HRs for the highest *vs.* lowest tertiles ranged from 1.4 to 1.6, indicating a modest association that, while statistically significant given the large sample size, has limited utility for individual clinical decision-making. Lastly, the study population was restricted to middle-aged and older adults, limiting generalisability to younger age groups.

## CONCLUSIONS

We provide observational evidence that non-conventional lipid indices are independently associated with incident hypertension risk among middle-aged and older Chinese adults. Composite lipid-inflammatory markers demonstrated modest incremental prognostic information beyond conventional lipids, though their standalone discriminatory performance was weak. These findings are hypothesis-generating and support continued investigation of integrated metabolic-inflammatory markers as supplementary components within multivariable risk assessment frameworks, pending external validation.

## Additional material


Online Supplementary Document

## Data Availability

**Data availability:** We utilised the publicly accessible CHARLS data set (https://charls.pku.edu.cn/).
