# Supplementary material for: Association of non-conventional lipid-inflammatory parameters with incident hypertension risk: a prospective cohort study
Source: J Glob Health. 2026 Aug 7;16:04237. doi: 10.7189/jogh.16.04237 (PMC13449563; doi:10.7189/jogh.16.04237)
Supplement: Supplementary file 1 [file jogh-16-04237-s001.pdf]

Supplement to: Zhao M, Wang M, Li Z, Wu Y, Lu F, Li Q. Association of non-conventional lipid-inflammatory parameters with incident hypertension risk: a prospective cohort study. J Glob Health. 2026;16:04237.

Table S1. Outline of JoGH's Guidelines for Reporting Analyses of Big Data Repositories Open to the Public (GRABDROP) items

JoGH guideline item	Author's Response
1. Please list all papers published by each co-author in previous 3 years that were based on secondary analysis of a big data repository	1.**Zhao M**, Xiao M, Tan Q, **Lu F**. Triglyceride glucose index as a predictor of mortality in middle-aged and elderly patients with type 2 diabetes in the US. Sci Rep. 2023;13(1):16478.<br>2.**Zhao M**, Xiao M, Tan Q, Ji J, **Lu F**. Cumulative residual cholesterol predicts the risk of cardiovascular disease in the general population aged 45 years and older. Lipids Health Dis. 2024;23(1):19.<br>3.Ji JJ, **Zhao MJ**, Xiao ML, Zhang HE, Tan Q, Cheng YR, **Lu F**. Association between relative muscle strength and cardiovascular disease among middle-aged and older adults in China. BMC Public Health. 2024;24(1):1928.<br>4.**Zhao M,** Cheng Y, Li M, Zhang W, Ji J, **Lu F**. Temporal changes in lipid concentrations and the prevalence of dyslipidemia among individuals with diabetes, prediabetes, and normal blood glucose from 2011 to 2015. Lipids Health Dis. 2024;23(1):394.<br>5.**Zhao M**, Xiao M, Zhang H, Tan Q, Ji J, Cheng Y, **Lu F**. combine and mediating effects of remnant cholesterol and renal function on hypertension risk in Chinese middle-aged and elderly people. Front Endocrinol (Lausanne). 2025;16:1442918.<br>6.**Zhao M**, Xiao M, Zhang H, Tan Q, Ji J, Cheng Y, **Lu F**. Relationship between plasma atherogenic index and incidence of cardiovascular diseases in Chinese middle-aged and elderly people. Sci Rep. 2025;15(1):8775.<br>7.Cao J, **Li Z**, **Wu Y**, Ni T, Zhang J, Xu Y, Zhu Z, Wang M, **Li Q**. Association of long-term exposure to air pollution with sleep among middle-aged and older adults in China: A nationwide study from 2015 to 2018. PLoS One. 2026;21(3):e0336665.
2. Please explain the key elements of your study design and the use of the available datasets that make your study an original scientific contribution	This study is among the first prospective investigations to examine composite lipid-inflammatory indices—combining eight non-conventional lipid parameters with hs-CRP—in relation to incident hypertension among middle-aged and older Chinese adults. Using repeated biomarker measurements from the China Health and Retirement Longitudinal Study, we constructed cumulative exposure metrics under a landmark cohort framework and applied a directed acyclic graph a priori to guide confounder adjustment, reducing the risk of overadjustment for mediators and colliders. These design elements may offer relevant observational evidence for lipid-hypertension associations in populations where Western-centric findings require further validation.

3. Please list all publications that addressed similar research questions in the same dataset and indicate where you cited them in your paper	Huang X, Li C, Zeng P, Ling Y, Tan S, Bai Z, et al. Non-traditional lipid-inflammatory parameters estimate the risk of stroke in middle-aged and older Chinese adults: a nationwide prospective cohort study. J Adv Res. 2025:S2090-1232(25)00924-5, cited as reference 9 in this paper.
4. Please explain how you addressed multiple testing through an appropriately rigorous statistical threshold and indicate this in the methods section	To address multiple testing, we pre-specified our primary inferences to the core exposure-outcome association examined in Tier 1. The significance of the primary associations was assessed using the Benjamini–Hochberg false discovery rate procedure, a robust method controlling the expected proportion of false discoveries across the eight lipid indices tested.
5. Please declare to what extent have AI chatbots been used in developing your paper and to which parts of the paper did they contribute	We declare that AI chatbots were used solely for language editing.

Table S2. Covariate classification based on directed acyclic graph

**Covariate Category**	**Included Variables**	**Inclusion / Exclusion Rationale**
Confounders (to be adjusted)	Age, gender, ethnicity, marital status, educational level, residential location, employment status, medical insurance type, smoking, alcohol consumption	These factors are common causes of lipid-inflammatory disorders and hypertension risk. Adjustment for these variables blocks the backdoor pathways in causal inference.
Intermediate mediators (not adjusted in primary analysis)	Body mass index (BMI), waist circumference, diabetes, heart disease, stroke, kidney disease	These variables lie on the causal pathway from dyslipidemia/inflammation to hypertension. Adjustment for such mediators will cause overadjustment bias and underestimate the true total effect.
Precision variables (adjusted in secondary models)	Sleep duration, life satisfaction	These variables are not confounders but are correlated with the study outcome. They can improve statistical efficiency and are only adjusted in secondary analysis models.
Colliders (not adjusted)	Lipid-lowering medication, hypoglycemic medication	These indicators are outcomes of the exposure (dyslipidemia) or confounded by medical consultation behaviors. Adjustment for colliders will open biased causal pathways and induce collider bias.

Table S3. Distribution of missing data

Variables	Deficiencies	
	**N**	**%**
Age	0	0
Gender	0	0
Residence	0	0
Education	10	0.1
Marital Status	5	0.1
Smoke	30	0.4
Disability	33	0.5
Drink	35	0.5
Stroke	51	0.7
Health insurance	54	0.8
HD	67	1
Kidney disease	71	1
DM	101	1.5
Antidiabetic drugs	102	1.5
Work	114	1.7
Hyperlipidemia	165	2.4
Lipid-lowering drug	167	2.4
Sleep duration	325	4.7
Nation	433	6.3
Life satisfaction	928	13.5
Waist	953	13.8
Weight	955	13.8
Height	970	14

HD – heart disease, DM – diabetes mellitus.

Table S4. Comparison of characteristics of included and excluded populations

Variable	N	Total (n = 17,708)	Exclusion (n = 10,817)	Inclusion (n = 6,891)	*P*	SMD
Age, years	17568	58.87 (10.17)	59.70 (10.98)	57.60 (8.62)	<0.001	0.21
Gender, n (%)	17706				0.004	0.04
Female		9,227 (52.11)	5,543 (51.25)	3,684 (53.46)		
Male		8,479 (47.89)	5,272 (48.75)	3,207 (46.54)		
Nation, n (%)	13567				0.09	0.03
Other		1,049 (7.73)	576 (8.10)	473 (7.32)		
Han ethnicity		12,518 (92.27)	6,533 (91.90)	5,985 (92.68)		
Education, n (%)	17653				0.006	0.04
Elementary school and below		11,755 (66.59)	7,089 (65.81)	4,666 (67.81)		
Above elementary school		5,898 (33.41)	3,683 (34.19)	2,215 (32.19)		
Marital Status, n (%)	17675				<0.001	0.16
Married		15,417 (87.22)	9,197 (85.24)	6,220 (90.33)		
Other		2,258 (12.78)	1,592 (14.76)	666 (9.67)		
Work, n (%)	17257				<0.001	0.31
No		6,394 (37.05)	4,495 (42.89)	1,899 (28.02)		
Yes		10,863 (62.95)	5,985 (57.11)	4,878 (71.98)		
Residence, n (%)	17708				<0.001	0.23
City		7,171 (40.50)	4,855 (44.88)	2,316 (33.61)		
Rural		10,537 (59.50)	5,962 (55.12)	4,575 (66.39)		
Health insurance, n (%)	17467				<0.001	0.09
No		1,179 (6.75)	810 (7.62)	369 (5.40)		
Yes		16,288 (93.25)	9,820 (92.38)	6,468 (94.60)		
Life satisfaction, n (%)	14362				>0.999	0
Satisfied		12,086 (84.15)	7,068 (84.15)	5,018 (84.15)		
Unsatisfied		2,276 (15.85)	1,331 (15.85)	945 (15.85)		
Disability, n (%)	17565				<0.001	0.1
No		14,421 (82.10)	8,638 (80.68)	5,783 (84.32)		

Yes		3,144 (17.90)	2,069 (19.32)	1,075 (15.68)		
Smoke, n (%)	17559				0.081	0.03
No		10,628 (60.53)	6,420 (60.01)	4,208 (61.33)		
Yes		6,931 (39.47)	4,278 (39.99)	2,653 (38.67)		
Drink, n (%)	17541				0.954	0
No		10,718 (61.10)	6,527 (61.09)	4,191 (61.13)		
Yes		6,823 (38.90)	4,158 (38.91)	2,665 (38.87)		
Sleep duration, h	16091	6.37 (1.90)	6.37 (1.92)	6.38 (1.86)	0.751	-0.01
Hyperlipidemia, n (%)	17213				<0.001	0.21
No		15,517 (90.15)	9,208 (87.80)	6,309 (93.80)		
Yes		1,696 (9.85)	1,279 (12.20)	417 (6.20)		
DM, n (%)	17408				<0.001	0.16
No		16,346 (93.90)	9,817 (92.46)	6,529 (96.16)		
Yes		1,062 (6.10)	801 (7.54)	261 (3.84)		
HD, n (%)	17467				<0.001	0.229
No		15,337 (87.81)	9,044 (84.98)	6,293 (92.22)		
Yes		2,130 (12.19)	1,599 (15.02)	531 (7.78)		
Stroke, n (%)	17526				<0.001	0.17
No		17,040 (97.23)	10,277 (96.17)	6,763 (98.87)		
Yes		486 (2.77)	409 (3.83)	77 (1.13)		
Kidney disease, n (%)	17452				0.476	0.01
No		16,473 (94.39)	10,025 (94.29)	6,448 (94.55)		
Yes		979 (5.61)	607 (5.71)	372 (5.45)		
Antidiabetic drugs, n (%)	17404				<0.001	0.15
No		16,692 (95.91)	10,063 (94.80)	6,629 (97.64)		
Yes		712 (4.09)	552 (5.20)	160 (2.36)		
Lipid lowering drug, n (%)	17201				<0.001	0.19
No		16,336 (94.97)	9,791 (93.45)	6,545 (97.34)		
Yes		865 (5.03)	686 (6.55)	179 (2.66)		

BMI, kg/m²	13616	23.47 (3.93)	23.74 (4.13)	23.11 (3.62)	<0.001	0.16
Waist, cm	13769	84.28 (12.62)	85.33 (12.86)	82.89 (12.17)	<0.001	0.2

Table S5. Associations of traditional and non-conventional lipid parameters with hypertension risk

Variable	Event, n(%)	Crude Model		Model 1		Model 2		
		HR (95%CI)	*P* value	HR (95%CI)	*P* value	HR (95%CI)	*P* value	Adjusted *P*
**Traditional lipid parameters**								
TC								
T1	764 (33.3)	Ref		Ref		Ref		
T2	838 (35.9)	1.10 (1.00 – 1.21)	0.054	1.10 (0.99 – 1.21)	0.066	1.10 (1.00 – 1.21)	0.057	0.057
T3	930 (41)	1.30 (1.18 – 1.43)	<0.001	1.27 (1.15 – 1.40)	<0.001	1.27 (1.15 – 1.39)	<0.001	<0.001
P for trend			<0.001		<0.001		<0.001	<0.001
TG								
T1	722 (31.1)	Ref		Ref		Ref		
T2	803 (35.6)	1.17 (1.06 – 1.29)	0.002	1.19 (1.07 – 1.31)	0.001	1.19 (1.08 – 1.32)	0.001	0.001
T3	1007 (43.6)	1.53 (1.39 – 1.68)	<0.001	1.58 (1.43 – 1.74)	<0.001	1.60 (1.46 – 1.77)	<0.001	<0.001
P for trend			<0.001		<0.001		<0.001	<0.001
HDL-C								
T1	953 (40.6)	Ref		Ref		Ref		
T2	800 (35.6)	0.85 (0.77 – 0.93)	0.001	0.84 (0.77 – 0.92)	<0.001	0.83 (0.76 – 0.91)	<0.001	<0.001
T3	779 (33.9)	0.80 (0.73 – 0.88)	<0.001	0.77 (0.70 – 0.85)	<0.001	0.75 (0.68 – 0.82)	<0.001	<0.001
P for trend			<0.001		<0.001		<0.001	<0.001
LDL-C								
T1	786 (34.5)	Ref		Ref		Ref		
T2	819 (35.7)	1.04 (0.94 – 1.15)	0.409	1.04 (0.94 – 1.15)	0.445	1.05 (0.95 – 1.16)	0.307	0.307
T3	927 (39.9)	1.19 (1.08 – 1.31)	<0.001	1.16 (1.05 – 1.27)	0.003	1.17 (1.06 – 1.29)	0.002	0.002
P for trend			<0.001		<0.001		0.001	0.001
**Non-conventional lipid parameters**								
AC								

T1	716 (31.2)	Ref		Ref		Ref		
T2	834 (36.3)	1.20 (1.09 – 1.33)	<0.001	1.23 (1.11 – 1.36)	<0.001	1.26 (1.14 – 1.39)	<0.001	<0.001
T3	982 (42.8)	1.49 (1.35 – 1.64)	<0.001	1.52 (1.38 – 1.67)	<0.001	1.57 (1.43 – 1.74)	<0.001	<0.001
P for trend			<0.001		<0.001		<0.001	<0.001
AIP								
T1	728 (31.7)	Ref		Ref		Ref		
T2	797 (34.7)	1.10 (1.00 – 1.22)	0.056	1.13 (1.02 – 1.25)	0.02	1.14 (1.03 – 1.26)	0.012	0.012
T3	1007 (43.8)	1.51 (1.37 – 1.66)	<0.001	1.57 (1.43 – 1.73)	<0.001	1.61 (1.46 – 1.78)	<0.001	<0.001
P for trend			<0.001		<0.001		<0.001	<0.001
CRI-I								
T1	716 (31.2)	Ref		Ref		Ref		
T2	834 (36.3)	1.20 (1.09 – 1.33)	<0.001	1.23 (1.11 – 1.36)	<0.001	1.26 (1.14 – 1.39)	<0.001	<0.001
T3	982 (42.8)	1.49 (1.35 – 1.64)	<0.001	1.52 (1.38 – 1.67)	<0.001	1.57 (1.43 – 1.74)	<0.001	<0.001
P for trend			<0.001		<0.001		<0.001	<0.001
CRI-II								
T1	739 (32.2)	Ref		Ref		Ref		
T2	844 (36.7)	1.17 (1.06 – 1.29)	0.002	1.18 (1.07 – 1.31)	0.001	1.21 (1.09 – 1.33)	<0.001	<0.001
T3	949 (41.3)	1.37 (1.24 – 1.50)	<0.001	1.37 (1.24 – 1.50)	<0.001	1.41 (1.28 – 1.55)	<0.001	<0.001
P for trend			<0.001		<0.001		<0.001	<0.001
LCI								
T1	716 (31.2)	Ref		Ref		Ref		
T2	804 (35)	1.15 (1.04 – 1.27)	0.008	1.15 (1.04 – 1.28)	0.005	1.18 (1.06 – 1.30)	0.002	0.002
T3	1012 (44.1)	1.55 (1.40 – 1.70)	<0.001	1.58 (1.43 – 1.74)	<0.001	1.61 (1.47 – 1.78)	<0.001	<0.001
P for trend			<0.001		<0.001		<0.001	<0.001
Non-HDL-C								
T1	734 (32.1)	Ref		Ref		Ref		
T2	836 (36.3)	1.17 (1.06 – 1.29)	0.002	1.17 (1.06 – 1.29)	0.002	1.19 (1.07 – 1.31)	0.001	0.001
T3	962 (41.9)	1.40 (1.27 – 1.54)	<0.001	1.38 (1.26 – 1.52)	<0.001	1.40 (1.27 – 1.55)	<0.001	<0.001
P for trend			<0.001		<0.001		<0.001	<0.001

RC								
T1	710 (31.6)	Ref		Ref		Ref		
T2	853 (35.9)	1.17 (1.06 – 1.29)	0.002	1.17 (1.06 – 1.29)	0.002	1.17 (1.06 – 1.30)	0.002	0.002
T3	969 (42.8)	1.47 (1.33 – 1.61)	<0.001	1.50 (1.37 – 1.66)	<0.001	1.51 (1.37 – 1.67)	<0.001	<0.001
P for trend			<0.001		<0.001		<0.001	<0.001
RC/HDL-C								
T1	747 (32.5)	Ref		Ref		Ref		
T2	798 (34.7)	1.08 (0.97 – 1.19)	0.145	1.09 (0.99 – 1.21)	0.087	1.10 (1.00 – 1.22)	0.059	0.059
T3	987 (43)	1.42 (1.29 – 1.56)	<0.001	1.47 (1.34 – 1.62)	<0.001	1.50 (1.36 – 1.65)	<0.001	<0.001
P for trend			<0.001		<0.001		<0.001	<0.001

Model 1: adjusted for age and gender.

Model 2: adjusted for Model 1 + ethnicity, marital status, educational level, residential location, employment status, medical insurance coverage, smoking and drinking history, sleep duration, life satisfaction.

AC – atherogenic coefficient, AIP – atherogenic index of plasma, LCI – lipoprotein combine index, Non-HDL-C – non-high-density lipoprotein cholesterol, CRI-I – Castelli risk index-I, CRI-II – Castelli risk index-II, RC – remnant cholesterol, RC/HDL-C – remnant cholesterol/high density lipoprotein cholesterol.

Table S6. AUC, optimal cut-off values, sensitivity, and specificity of lipid parameters in hypertension prediction

Variables	AUC	95% CI	Best threshold	Specificity	Sensitivity	*P* value
**Traditional lipid parameters**						
TC	0.540	0.526–0.554	191	0.488	0.581	< 0.001
TG	0.565	0.551–0.579	126	0.673	0.432	< 0.001
HDL-C	0.536	0.522–0.550	52.1	0.552	0.512	< 0.001
LDL-C	0.527	0.513–0.541	118	0.795	0.254	< 0.001
**Non-conventional lipid parameters**						
AC	0.560	0.546–0.574	2.98	0.593	0.513	< 0.001
AIP	0.563	0.548–0.577	0.325	0.519	0.589	< 0.001
CRI-I	0.560	0.546–0.574	3.98	0.593	0.513	< 0.001
CRI-II	0.546	0.532–0.560	2.36	0.46	0.63	< 0.001
LCI	0.568	0.554–0.582	65830	0.69	0.418	< 0.001
Non-HDL-C	0.554	0.539–0.568	139	0.464	0.621	< 0.001

RC	0.558	0.544–0.572	24.6	0.693	0.396	< 0.001
RC/HDL-C	0.559	0.545–0.573	0.589	0.581	0.518	< 0.001
**Non-conventional lipid-inflammatory parameters**						
AC-CRP	0.563	0.549–0.577	0.704	0.524	0.584	< 0.001
AIP-CRP	0.568	0.554–0.582	0.079	0.67	0.45	< 0.001
CRI-I-CRP	0.561	0.547–0.575	0.93	0.496	0.605	< 0.001
CRI-II-CRP	0.558	0.544–0.572	0.553	0.513	0.588	< 0.001
LCI-CRP	0.575	0.561–0.589	15076	0.687	0.434	< 0.001
Non-HDL-C-CRP	0.560	0.546–0.574	31.3	0.439	0.665	< 0.001
RC-CRP	0.568	0.554–0.583	5.74	0.525	0.592	< 0.001
RC/HDL-C-CRP	0.568	0.554–0.582	0.151	0.613	0.497	< 0.001

AUC – area under the curve, CI – confidence interval, AC – atherogenic coefficient, AIP – atherogenic index of plasma, LCI – lipoprotein combine index, Non-HDL-C – non-high-density lipoprotein cholesterol, CRI-I – Castelli risk index-I, CRI-II – Castelli risk index-II, RC – remnant cholesterol, RC/HDL-C – remnant cholesterol/high density lipoprotein cholesterol, CRP – C-reactive protein.

Table S7. AUC, optimal cut-off values, sensitivity, and specificity of cumulative lipid parameters in hypertension prediction

Variables	AUC	95% CI	Best threshold	Specificity	Sensitivity	*P* value
**Traditional lipid parameters**						
Cum_TC	0.541	0.522–0.561	568	0.717	0.359	< 0.001
Cum_TG	0.562	0.542–0.581	385	0.554	0.552	< 0.001
Cum_HDL-C	0.537	0.517–0.556	159	0.738	0.332	< 0.001
Cum_LDL-C	0.533	0.513–0.553	330	0.747	0.313	< 0.001
**Non-conventional lipid parameters**						
Cum_AC	0.562	0.543–0.582	8.31	0.466	0.642	< 0.001
Cum_AIP	0.563	0.543–0.582	1.02	0.524	0.577	< 0.001
Cum_CRI-I	0.562	0.543–0.582	11.3	0.466	0.642	< 0.001
Cum_CRI-II	0.551	0.532–0.571	6.53	0.467	0.624	< 0.001
Cum_LCI	0.568	0.548–0.587	181290	0.641	0.46	< 0.001
Cum_Non-HDL-C	0.555	0.536–0.575	409	0.622	0.47	< 0.001
Cum_RC	0.560	0.540–0.579	81.1	0.713	0.391	< 0.001

Cum_RC/HDL-C	0.561	0.541–0.581	1.78	0.462	0.635	< 0.001
**Non-conventional lipid-inflammatory parameters**						
Cum_AC-CRP	0.573	0.553–0.592	2.19	0.443	0.682	< 0.001
Cum_AIP-CRP	0.570	0.550–0.589	0.27	0.629	0.5	< 0.001
Cum_CRI-I-CRP	0.571	0.552–0.590	2.89	0.513	0.614	< 0.001
Cum_CRI-II-CRP	0.571	0.551–0.590	1.61	0.453	0.668	< 0.001
Cum_LCI-CRP	0.579	0.560–0.598	63748	0.577	0.556	< 0.001
Cum_Non-HDL-C-CRP	0.571	0.552–0.590	95.9	0.512	0.621	< 0.001
Cum_RC-CRP	0.570	0.551–0.590	21.9	0.551	0.58	< 0.001
Cum_RC/HDL-C-CRP	0.571	0.551–0.590	0.574	0.463	0.666	< 0.001

AUC – area under the curve, CI – confidence interval, AC – atherogenic coefficient, AIP – atherogenic index of plasma, LCI – lipoprotein combine index, Non-HDL-C – non-high-density lipoprotein cholesterol, CRI-I – Castelli risk index-I, CRI-II – Castelli risk index-II, RC – remnant cholesterol, RC/HDL-C – remnant cholesterol/high density lipoprotein cholesterol, CRP – C-reactive protein.

Table S8. Incremental predictive value of non-conventional lipid and lipid-inflammatory parameters in hypertension prediction

	NRI		IDI	
	**Estimate (95%CI)**	***P* value**	**Estimate (95%CI)**	***P* value**
Basic model	Ref		Ref	
Basic model + CRP	0.046 (0.022 – 0.071)	<0.001	0.005 (0.002 – 0.008)	<0.001
Basic model + AC	0.100 (0.074 – 0.128)	<0.001	0.011 (0.006 – 0.016)	<0.001
Basic model + AC + CRP	0.094 (0.070 – 0.123)	<0.001	0.014 (0.009 – 0.021)	<0.001
Basic model + AIP	0.117 (0.087 – 0.140)	<0.001	0.013 (0.007 – 0.019)	<0.001
Basic model + AIP + CRP	0.105 (0.077 – 0.128)	<0.001	0.016 (0.010 – 0.023)	<0.001
Basic model + CRI-I	0.100 (0.074 – 0.128)	<0.001	0.011 (0.006 – 0.016)	<0.001
Basic model + CRI-I + CRP	0.094 (0.070 – 0.123)	<0.001	0.014 (0.009 – 0.021)	<0.001
Basic model + CRI-II	0.076 (0.047 – 0.107)	<0.001	0.006 (0.003 – 0.010)	<0.001
Basic model + CRI-II + CRP	0.084 (0.057 – 0.112)	<0.001	0.009 (0.006 – 0.016)	<0.001
Basic model + LCI	0.112 (0.085 – 0.140)	<0.001	0.012 (0.008 – 0.018)	<0.001
Basic model + LCI + CRP	0.106 (0.078 – 0.133)	<0.001	0.015 (0.010 – 0.023)	<0.001
Basic model + Non-HDL-C	0.068 (0.044 – 0.101)	<0.001	0.006 (0.003 – 0.010)	<0.001

Basic model + Non-HDL-C + CRP	0.081 (0.055 – 0.111)	<0.001	0.010 (0.006 – 0.015)	<0.001
Basic model + RC	0.096 (0.061 – 0.130)	<0.001	0.009 (0.005 – 0.014)	<0.001
Basic model + RC + CRP	0.094 (0.071 – 0.125)	<0.001	0.013 (0.008 – 0.019)	<0.001
Basic model + RC/HDL-C	0.100 (0.071 – 0.125)	<0.001	0.009 (0.005 – 0.014)	<0.001
Basic model + RC/HDL-C + CRP	0.087 (0.066 – 0.114)	<0.001	0.012 (0.008 – 0.019)	<0.001

The baseline model includes variables such as age, gender, ethnicity, marital status, education level, place of residence, employment status, health insurance, smoking and drinking history, sleep duration, life satisfaction. NRI – net reclassification index, IDI – integrated discrimination improvement index, CI – confidence interval, AC – atherogenic coefficient, AIP – atherogenic index of plasma, LCI – lipoprotein combine index, Non-HDL-C – non-high-density lipoprotein cholesterol, CRI-I – Castelli risk index-I, CRI-II – Castelli risk index-II, RC – remnant cholesterol, RC/HDL-C – remnant cholesterol/high density lipoprotein cholesterol, CRP – C-reactive protein.

Table S9. Incremental predictive value of cumulative non-conventional lipid and lipid-inflammatory parameters in hypertension prediction

	**NRI**		**IDI**	
	**Estimate (95%CI)**	***P* value**	**Estimate (95%CI)**	***P* value**
Basic model	Ref		Ref	
Basic model + Cum_CRP	0.080 (0.035 – 0.122)	<0.001	0.007 (0.002 – 0.013)	<0.001
Basic model + Cum_AC	0.105 (0.055 – 0.148)	<0.001	0.010 (0.005 – 0.017)	<0.001
Basic model + Cum_AC + CRP	0.123 (0.074 – 0.155)	<0.001	0.015 (0.008 – 0.023)	<0.001
Basic model + Cum_AIP	0.099 (0.057 – 0.144)	<0.001	0.011 (0.005 – 0.018)	<0.001
Basic model + Cum_AIP + CRP	0.114 (0.075 – 0.154)	<0.001	0.015 (0.009 – 0.024)	<0.001
Basic model + Cum_CRI-I	0.105 (0.055 – 0.148)	<0.001	0.010 (0.005 – 0.017)	<0.001
Basic model + Cum_CRI-I + CRP	0.123 (0.074 – 0.155)	<0.001	0.015 (0.008 – 0.023)	<0.001
Basic model + Cum_CRI-II	0.078 (0.029 – 0.116)	0.007	0.006 (0.002 – 0.012)	<0.001
Basic model + Cum_CRI-II + CRP	0.080 (0.035 – 0.122)	<0.001	0.007 (0.002 – 0.013)	<0.001
Basic model + Cum_LCI	0.081 (0.034 – 0.130)	<0.001	0.010 (0.005 – 0.017)	<0.001
Basic model + Cum_LCI + CRP	0.117 (0.075 – 0.158)	<0.001	0.015 (0.008 – 0.025)	<0.001
Basic model + Cum_Non-HDL-C	0.052 (0.007 – 0.091)	0.013	0.005 (0.001 – 0.012)	<0.001
Basic model + Cum_Non-HDL-C + CRP	0.096 (0.055 – 0.137)	<0.001	0.011 (0.005 – 0.020)	<0.001
Basic model + Cum_RC	0.077 (0.030 – 0.114)	<0.001	0.008 (0.003 – 0.014)	<0.001
Basic model + Cum_RC + CRP	0.109 (0.067 – 0.154)	<0.001	0.013 (0.006 – 0.021)	<0.001

Basic model + Cum_RC/HDL-C	0.097 (0.057 – 0.142)	<0.001	0.010 (0.005 – 0.017)	<0.001
Basic model + Cum_RC/HDL-C + CRP	0.127 (0.088 – 0.165)	<0.001	0.014 (0.008 – 0.023)	<0.001

The baseline model includes variables such as age, gender, ethnicity, marital status, education level, place of residence, employment status, health insurance, smoking and drinking history, sleep duration, life satisfaction. NRI – net reclassification index, IDI – integrated discrimination improvement index, CI – confidence interval, AC – atherogenic coefficient, AIP – atherogenic index of plasma, LCI – lipoprotein combine index, Non-HDL-C – non-high-density lipoprotein cholesterol, CRI-I – Castelli risk index-I, CRI-II – Castelli risk index-II, RC – remnant cholesterol, RC/HDL-C – remnant cholesterol/high density lipoprotein cholesterol, CRP – C-reactive protein.

Table S10. Subgroup analysis of non-conventional lipid parameters

**Subgroup**	**Variable**	**HR (95%CI)**	***P* value**	***P* for interaction**	**Variable**	**HR (95%CI)**	***P* value**	***P* for interaction**
Age				0.034				0.07
< 60	AC_T1	Ref			AIP_T1	Ref		
	AC_T2	1.32 (1.15 – 1.51)	<0.001		AIP_T2	1.26 (1.10 – 1.45)	0.001	
	AC_T3	1.75 (1.53 – 2.00)	<0.001		AIP_T3	1.73 (1.52 – 1.98)	<0.001	
≥ 60	AC_T1	Ref			AIP_T1	Ref		
	AC_T2	1.18 (1.02 – 1.36)	0.03		AIP_T2	1.00 (0.86 – 1.15)	0.953	
	AC_T3	1.34 (1.16 – 1.55)	<0.001		AIP_T3	1.43 (1.25 – 1.65)	<0.001	
Gender				0.256				0.668
Female	AC_T1	Ref			AIP_T1	Ref		
	AC_T2	1.24 (1.08 – 1.43)	0.002		AIP_T2	1.15 (1.00 – 1.33)	0.049	
	AC_T3	1.46 (1.27 – 1.67)	<0.001		AIP_T3	1.57 (1.37 – 1.80)	<0.001	
Male	AC_T1	Ref			AIP_T1	Ref		
	AC_T2	1.27 (1.09 – 1.46)	0.002		AIP_T2	1.11 (0.96 – 1.28)	0.175	
	AC_T3	1.68 (1.46 – 1.93)	<0.001		AIP_T3	1.62 (1.41 – 1.86)	<0.001	
Education				0.315				0.375
Elementary school and below	AC_T1	Ref			AIP_T1	Ref		
	AC_T2	1.22 (1.08 – 1.37)	0.001		AIP_T2	1.09 (0.97 – 1.23)	0.141	
	AC_T3	1.50 (1.33 – 1.68)	<0.001		AIP_T3	1.56 (1.39 – 1.75)	<0.001	
Above elementary school	AC_T1	Ref			AIP_T1	Ref		
	AC_T2	1.39 (1.14 – 1.69)	0.001		AIP_T2	1.28 (1.05 – 1.56)	0.014	

	AC_T3	1.81 (1.49 – 2.19)	<0.001		AIP_T3	1.77 (1.47 – 2.13)	<0.001	
Work				0.849				0.626
No	AC_T1	Ref			AIP_T1	Ref		
	AC_T2	1.20 (0.99 – 1.45)	0.06		AIP_T2	1.06 (0.88 – 1.27)	0.567	
	AC_T3	1.51 (1.26 – 1.81)	<0.001		AIP_T3	1.57 (1.32 – 1.87)	<0.001	
Yes	AC_T1	Ref			AIP_T1	Ref		
	AC_T2	1.28 (1.14 – 1.45)	<0.001		AIP_T2	1.18 (1.04 – 1.33)	0.008	
	AC_T3	1.60 (1.43 – 1.80)	<0.001		AIP_T3	1.64 (1.46 – 1.84)	<0.001	
Residence				0.177				0.63
City	AC_T1	Ref			AIP_T1	Ref		
	AC_T2	1.27 (1.04 – 1.55)	0.019		AIP_T2	1.14 (0.94 – 1.38)	0.193	
	AC_T3	1.75 (1.46 – 2.11)	<0.001		AIP_T3	1.68 (1.41 – 2.01)	<0.001	
Rural	AC_T1				AIP_T1	Ref		
	AC_T2	1.26 (1.12 – 1.42)	<0.001		AIP_T2	1.14 (1.01 – 1.28)	0.029	
	AC_T3	1.50 (1.34 – 1.69)	<0.001		AIP_T3	1.58 (1.41 – 1.77)	<0.001	
Smoke				0.99				0.693
No	AC_T1	Ref			AIP_T1	Ref		
	AC_T2	1.27 (1.12 – 1.44)	<0.001		AIP_T2	1.18 (1.03 – 1.34)	0.014	
	AC_T3	1.58 (1.39 – 1.79)	<0.001		AIP_T3	1.66 (1.47 – 1.89)	<0.001	
Yes	AC_T1	Ref			AIP_T1	Ref		
	AC_T2	1.25 (1.07 – 1.47)	0.006		AIP_T2	1.09 (0.93 – 1.28)	0.292	
	AC_T3	1.57 (1.34 – 1.83)	<0.001		AIP_T3	1.54 (1.32 – 1.79)	<0.001	
Drink				0.443				0.548
No	AC_T1	Ref			AIP_T1	Ref		
	AC_T2	1.27 (1.11 – 1.45)	<0.001		AIP_T2	1.14 (1.00 – 1.30)	0.053	
	AC_T3	1.52 (1.34 – 1.73)	<0.001		AIP_T3	1.56 (1.37 – 1.77)	<0.001	
Yes	AC_T1	Ref			AIP_T1	Ref		
	AC_T2	1.25 (1.07 – 1.46)	0.006		AIP_T2	1.14 (0.97 – 1.33)	0.105	
	AC_T3	1.66 (1.42 – 1.93)	<0.001		AIP_T3	1.68 (1.45 – 1.95)	<0.001	

Life satisfaction				0.034				0.352
Satisfied	AC_T1	Ref			AIP_T1	Ref		
	AC_T2	1.29 (1.16 – 1.44)	<0.001		AIP_T2	1.15 (1.03 – 1.29)	0.011	
	AC_T3	1.66 (1.49 – 1.85)	<0.001		AIP_T3	1.66 (1.49 – 1.84)	<0.001	
Unsatisfied	AC_T1	Ref			AIP_T1	Ref		
	AC_T2	1.13 (0.89 – 1.45)	0.314		AIP_T2	1.07 (0.84 – 1.38)	0.585	
	AC_T3	1.22 (0.96 – 1.56)	0.111		AIP_T3	1.41 (1.11 – 1.79)	0.005	
Antidiabetic drugs				0.566				0.28
No	AC_T1	Ref			AIP_T1	Ref		
	AC_T2	1.25 (1.13 – 1.39)	<0.001		AIP_T2	1.12 (1.02 – 1.25)	0.025	
	AC_T3	1.56 (1.41 – 1.72)	<0.001		AIP_T3	1.59 (1.45 – 1.76)	<0.001	
Yes	AC_T1	Ref			AIP_T1	Ref		
	AC_T2	1.38 (0.70 – 2.72)	0.345		AIP_T2	1.79 (0.89 – 3.59)	0.104	
	AC_T3	1.97 (1.06 – 3.65)	0.031		AIP_T3	2.33 (1.21 – 4.49)	0.011	
Lipid-lowering drug				0.944				0.373
No	AC_T1	Ref			AIP_T1	Ref		
	AC_T2	1.25 (1.13 – 1.39)	<0.001		AIP_T2	1.14 (1.03 – 1.26)	0.012	
	AC_T3	1.56 (1.42 – 1.73)	<0.001		AIP_T3	1.62 (1.47 – 1.79)	<0.001	
Yes	AC_T1	Ref			AIP_T1	Ref		
	AC_T2	1.14 (0.56 – 2.31)	0.723		AIP_T2	0.91 (0.47 – 1.74)	0.773	
	AC_T3	1.44 (0.75 – 2.77)	0.27		AIP_T3	1.18 (0.67 – 2.08)	0.575	
Age				0.034				0.192
< 60	CRI-I_T1	Ref			CRI-II_T1	Ref		
	CRI-I_T2	1.32 (1.15 – 1.51)	<0.001		CRI-II_T2	1.27 (1.11 – 1.46)	<0.001	
	CRI-I_T3	1.75 (1.53 – 2.00)	<0.001		CRI-II_T3	1.53 (1.34 – 1.75)	<0.001	
≥ 60	CRI-I_T1	Ref			CRI-II_T1	Ref		
	CRI-I_T2	1.18 (1.02 – 1.36)	0.03		CRI-II_T2	1.16 (1.00 – 1.34)	0.051	
	CRI-I_T3	1.34 (1.16 – 1.55)	<0.001		CRI-II_T3	1.27 (1.10 – 1.46)	0.001	
Gender				0.256				0.046

Female	CRI-I_T1	Ref			CRI-II_T1	Ref		
	CRI-I_T2	1.24 (1.08 – 1.43)	0.002		CRI-II_T2	1.25 (1.09 – 1.43)	0.001	
	CRI-I_T3	1.46 (1.27 – 1.67)	<0.001		CRI-II_T3	1.29 (1.13 – 1.48)	<0.001	
Male	CRI-I_T1	Ref			CRI-II_T1	Ref		
	CRI-I_T2	1.27 (1.09 – 1.46)	0.002		CRI-II_T2	1.17 (1.01 – 1.36)	0.031	
	CRI-I_T3	1.68 (1.46 – 1.93)	<0.001		CRI-II_T3	1.53 (1.33 – 1.75)	<0.001	
Education				0.315				0.369
Elementary school and below	CRI-I_T1	Ref			CRI-II_T1	Ref		
	CRI-I_T2	1.22 (1.08 – 1.37)	0.001		CRI-II_T2	1.18 (1.06 – 1.33)	0.004	
	CRI-I_T3	1.50 (1.33 – 1.68)	<0.001		CRI-II_T3	1.34 (1.19 – 1.50)	<0.001	
Above elementary school	CRI-I_T1	Ref			CRI-II_T1	Ref		
	CRI-I_T2	1.39 (1.14 – 1.69)	0.001		CRI-II_T2	1.29 (1.06 – 1.57)	0.011	
	CRI-I_T3	1.81 (1.49 – 2.19)	<0.001		CRI-II_T3	1.60 (1.33 – 1.93)	<0.001	
Work				0.849				0.211
No	CRI-I_T1	Ref			CRI-II_T1	Ref		
	CRI-I_T2	1.20 (0.99 – 1.45)	0.06		CRI-II_T2	1.21 (1.01 – 1.46)	0.038	
	CRI-I_T3	1.51 (1.26 – 1.81)	<0.001		CRI-II_T3	1.27 (1.06 – 1.52)	0.009	
Yes	CRI-I_T1	Ref			CRI-II_T1	Ref		
	CRI-I_T2	1.28 (1.14 – 1.45)	<0.001		CRI-II_T2	1.20 (1.07 – 1.35)	0.002	
	CRI-I_T3	1.60 (1.43 – 1.80)	<0.001		CRI-II_T3	1.48 (1.31 – 1.66)	<0.001	
Residence				0.177				0.325
City	CRI-I_T1	Ref			CRI-II_T1	Ref		
	CRI-I_T2	1.27 (1.04 – 1.55)	0.019		CRI-II_T2	1.10 (0.91 – 1.33)	0.306	
	CRI-I_T3	1.75 (1.46 – 2.11)	<0.001		CRI-II_T3	1.42 (1.19 – 1.70)	<0.001	
Rural	CRI-I_T1	Ref			CRI-II_T1	Ref		
	CRI-I_T2	1.26 (1.12 – 1.42)	<0.001		CRI-II_T2	1.26 (1.12 – 1.42)	<0.001	
	CRI-I_T3	1.50 (1.34 – 1.69)	<0.001		CRI-II_T3	1.40 (1.24 – 1.57)	<0.001	
Smoke				0.99				0.388
No	CRI-I_T1	Ref			CRI-II_T1	Ref		

	CRI-I_T2	1.27 (1.12 – 1.44)	<0.001		CRI-II_T2	1.25 (1.10 – 1.42)	0.001	
	CRI-I_T3	1.58 (1.39 – 1.79)	<0.001		CRI-II_T3	1.38 (1.22 – 1.57)	<0.001	
Yes	CRI-I_T1	Ref			CRI-II_T1	Ref		
	CRI-I_T2	1.25 (1.07 – 1.47)	0.006		CRI-II_T2	1.15 (0.98 – 1.35)	0.088	
	CRI-I_T3	1.57 (1.34 – 1.83)	<0.001		CRI-II_T3	1.45 (1.25 – 1.69)	<0.001	
Drink				0.443				0.284
No	CRI-I_T1	Ref			CRI-II_T1	Ref		
	CRI-I_T2	1.27 (1.11 – 1.45)	<0.001		CRI-II_T2	1.25 (1.10 – 1.42)	0.001	
	CRI-I_T3	1.52 (1.34 – 1.73)	<0.001		CRI-II_T3	1.37 (1.20 – 1.56)	<0.001	
Yes	CRI-I_T1	Ref			CRI-II_T1	Ref		
	CRI-I_T2	1.25 (1.07 – 1.46)	0.006		CRI-II_T2	1.17 (1.00 – 1.36)	0.048	
	CRI-I_T3	1.66 (1.42 – 1.93)	<0.001		CRI-II_T3	1.48 (1.28 – 1.72)	<0.001	
Life satisfaction				0.034				0.067
Satisfied	CRI-I_T1	Ref			CRI-II_T1	Ref		
	CRI-I_T2	1.29 (1.16 – 1.44)	<0.001		CRI-II_T2	1.24 (1.11 – 1.38)	<0.001	
	CRI-I_T3	1.66 (1.49 – 1.85)	<0.001		CRI-II_T3	1.48 (1.33 – 1.64)	<0.001	
Unsatisfied	CRI-I_T1	Ref			CRI-II_T1	Ref		
	CRI-I_T2	1.13 (0.89 – 1.45)	0.314		CRI-II_T2	1.08 (0.85 – 1.38)	0.512	
	CRI-I_T3	1.22 (0.96 – 1.56)	0.111		CRI-II_T3	1.12 (0.88 – 1.43)	0.361	
Antidiabetic drugs				0.566				0.547
No	CRI-I_T1	Ref			CRI-II_T1	Ref		
	CRI-I_T2	1.25 (1.13 – 1.39)	<0.001		CRI-II_T2	1.20 (1.09 – 1.33)	<0.001	
	CRI-I_T3	1.56 (1.41 – 1.72)	<0.001		CRI-II_T3	1.39 (1.26 – 1.54)	<0.001	
Yes	CRI-I_T1	Ref			CRI-II_T1	Ref		
	CRI-I_T2	1.38 (0.70 – 2.72)	0.345		CRI-II_T2	1.37 (0.72 – 2.61)	0.341	
	CRI-I_T3	1.97 (1.06 – 3.65)	0.031		CRI-II_T3	1.60 (0.86 – 2.98)	0.138	
Lipid-lowering drug				0.944				0.964
No	CRI-I_T1	Ref			CRI-II_T1	Ref		
	CRI-I_T2	1.25 (1.13 – 1.39)	<0.001		CRI-II_T2	1.20 (1.09 – 1.33)	<0.001	

	CRI-I_T3	1.56 (1.42 – 1.73)	<0.001		CRI-II_T3	1.40 (1.27 – 1.55)	<0.001	
Yes	CRI-I_T1	Ref			CRI-II_T1	Ref		
	CRI-I_T2	1.14 (0.56 – 2.31)	0.723		CRI-II_T2	0.95 (0.48 – 1.87)	0.875	
	CRI-I_T3	1.44 (0.75 – 2.77)	0.27		CRI-II_T3	1.21 (0.65 – 2.26)	0.554	
Age				0.017				0.226
< 60	LCI_T1	Ref			Non-HDL-C_T1	Ref		
	LCI_T2	1.34 (1.16 – 1.54)	<0.001		Non-HDL-C_T2	1.27 (1.11 – 1.45)	0.001	
	LCI_T3	1.79 (1.57 – 2.05)	<0.001		Non-HDL-C_T3	1.51 (1.33 – 1.72)	<0.001	
≥ 60	LCI_T1	Ref			Non-HDL-C_T1	Ref		
	LCI_T2	1.00 (0.86 – 1.16)	0.986		Non-HDL-C_T2	1.08 (0.93 – 1.26)	0.289	
	LCI_T3	1.39 (1.20 – 1.61)	<0.001		Non-HDL-C_T3	1.26 (1.08 – 1.45)	0.002	
Gender				0.056				0.143
Female	LCI_T1	Ref			Non-HDL-C_T1	Ref		
	LCI_T2	1.15 (0.99 – 1.32)	0.061		Non-HDL-C_T2	1.14 (0.99 – 1.31)	0.07	
	LCI_T3	1.45 (1.26 – 1.66)	<0.001		Non-HDL-C_T3	1.26 (1.10 – 1.44)	0.001	
Male	LCI_T1	Ref			Non-HDL-C_T1	Ref		
	LCI_T2	1.19 (1.03 – 1.38)	0.016		Non-HDL-C_T2	1.22 (1.06 – 1.41)	0.005	
	LCI_T3	1.79 (1.55 – 2.05)	<0.001		Non-HDL-C_T3	1.54 (1.34 – 1.77)	<0.001	
Education				0.401				0.15
Elementary school and below	LCI_T1	Ref			Non-HDL-C_T1	Ref		
	LCI_T2	1.13 (1.01 – 1.28)	0.038		Non-HDL-C_T2	1.15 (1.03 – 1.30)	0.016	
	LCI_T3	1.55 (1.38 – 1.73)	<0.001		Non-HDL-C_T3	1.31 (1.17 – 1.47)	<0.001	
Above elementary school	LCI_T1	Ref			Non-HDL-C_T1	Ref		
	LCI_T2	1.29 (1.06 – 1.58)	0.01		Non-HDL-C_T2	1.28 (1.06 – 1.55)	0.012	
	LCI_T3	1.80 (1.50 – 2.18)	<0.001		Non-HDL-C_T3	1.65 (1.37 – 1.99)	<0.001	
Work				0.017				0.86
No	LCI_T1	Ref			Non-HDL-C_T1	Ref		
	LCI_T2	0.94 (0.78 – 1.14)	0.535		Non-HDL-C_T2	1.15 (0.96 – 1.39)	0.128	
	LCI_T3	1.49 (1.25 – 1.78)	<0.001		Non-HDL-C_T3	1.35 (1.13 – 1.62)	0.001	

Yes	LCI_T1	Ref			Non-HDL-C_T1	Ref		
	LCI_T2	1.29 (1.14 – 1.45)	<0.001		Non-HDL-C_T2	1.20 (1.07 – 1.35)	0.003	
	LCI_T3	1.66 (1.48 – 1.87)	<0.001		Non-HDL-C_T3	1.43 (1.27 – 1.61)	<0.001	
Residence				0.417				0.638
City	LCI_T1	Ref			Non-HDL-C_T1	Ref		
	LCI_T2	1.12 (0.92 – 1.36)	0.253		Non-HDL-C_T2	1.20 (0.99 – 1.45)	0.06	
	LCI_T3	1.67 (1.40 – 2.00)	<0.001		Non-HDL-C_T3	1.49 (1.25 – 1.78)	<0.001	
Rural	LCI_T1	Ref			Non-HDL-C_T1	Ref		
	LCI_T2	1.20 (1.07 – 1.36)	0.002		Non-HDL-C_T2	1.19 (1.06 – 1.34)	0.004	
	LCI_T3	1.59 (1.41 – 1.79)	<0.001		Non-HDL-C_T3	1.38 (1.22 – 1.55)	<0.001	
Smoke				0.316				0.145
No	LCI_T1	Ref			Non-HDL-C_T1	Ref		
	LCI_T2	1.22 (1.06 – 1.39)	0.004		Non-HDL-C_T2	1.18 (1.04 – 1.35)	0.01	
	LCI_T3	1.58 (1.39 – 1.79)	<0.001		Non-HDL-C_T3	1.31 (1.16 – 1.49)	<0.001	
Yes	LCI_T1	Ref			Non-HDL-C_T1	Ref		
	LCI_T2	1.12 (0.96 – 1.31)	0.165		Non-HDL-C_T2	1.17 (1.00 – 1.37)	0.048	
	LCI_T3	1.68 (1.44 – 1.96)	<0.001		Non-HDL-C_T3	1.55 (1.33 – 1.81)	<0.001	
Drink				0.372				0.629
No	LCI_T1	Ref			Non-HDL-C_T1	Ref		
	LCI_T2	1.16 (1.02 – 1.33)	0.027		Non-HDL-C_T2	1.16 (1.02 – 1.32)	0.028	
	LCI_T3	1.53 (1.35 – 1.74)	<0.001		Non-HDL-C_T3	1.34 (1.18 – 1.53)	<0.001	
Yes	LCI_T1	Ref			Non-HDL-C_T1	Ref		
	LCI_T2	1.20 (1.03 – 1.40)	0.023		Non-HDL-C_T2	1.22 (1.05 – 1.42)	0.011	
	LCI_T3	1.74 (1.50 – 2.03)	<0.001		Non-HDL-C_T3	1.50 (1.29 – 1.74)	<0.001	
Life satisfaction				0.033				0.135
Satisfied	LCI_T1	Ref			Non-HDL-C_T1	Ref		
	LCI_T2	1.16 (1.04 – 1.30)	0.007		Non-HDL-C_T2	1.19 (1.07 – 1.33)	0.002	
	LCI_T3	1.68 (1.51 – 1.87)	<0.001		Non-HDL-C_T3	1.46 (1.31 – 1.62)	<0.001	
Unsatisfied	LCI_T1	Ref			Non-HDL-C_T1	Ref		

	LCI_T2	1.25 (0.98 – 1.60)	0.071		Non-HDL-C_T2	1.20 (0.94 – 1.53)	0.142	
	LCI_T3	1.32 (1.03 – 1.68)	0.029		Non-HDL-C_T3	1.17 (0.92 – 1.49)	0.211	
Antidiabetic drugs				0.395				0.372
No	LCI_T1	Ref			Non-HDL-C_T1	Ref		
	LCI_T2	1.16 (1.05 – 1.29)	0.004		Non-HDL-C_T2	1.17 (1.06 – 1.30)	0.002	
	LCI_T3	1.60 (1.45 – 1.77)	<0.001		Non-HDL-C_T3	1.40 (1.27 – 1.55)	<0.001	
Yes	LCI_T1	Ref			Non-HDL-C_T1	Ref		
	LCI_T2	1.56 (0.82 – 2.98)	0.175		Non-HDL-C_T2	1.62 (0.85 – 3.09)	0.142	
	LCI_T3	1.78 (0.96 – 3.29)	0.065		Non-HDL-C_T3	1.48 (0.79 – 2.76)	0.217	
Lipid-lowering drug				0.243				0.500
No	LCI_T1	Ref			Non-HDL-C_T1	Ref		
	LCI_T2	1.17 (1.06 – 1.30)	0.002		Non-HDL-C_T2	1.18 (1.07 – 1.31)	0.001	
	LCI_T3	1.62 (1.47 – 1.79)	<0.001		Non-HDL-C_T3	1.41 (1.28 – 1.56)	<0.001	
Yes	LCI_T1	Ref			Non-HDL-C_T1	Ref		
	LCI_T2	0.81 (0.42 – 1.54)	0.513		Non-HDL-C_T2	1.03 (0.55 – 1.93)	0.925	
	LCI_T3	0.97 (0.52 – 1.79)	0.912		Non-HDL-C_T3	0.97 (0.54 – 1.74)	0.928	
Age				0.132				0.350
< 60	RC_T1	Ref			RC/HDL-C_T1	Ref		
	RC_T2	1.28 (1.11 – 1.47)	<0.001		RC/HDL-C_T2	1.15 (1.00 – 1.33)	0.043	
	RC_T3	1.63 (1.43 – 1.86)	<0.001		RC/HDL-C_T3	1.58 (1.38 – 1.80)	<0.001	
≥ 60	RC_T1	Ref			RC/HDL-C_T1	Ref		
	RC_T2	1.06 (0.92 – 1.22)	0.445		RC/HDL-C_T2	1.03 (0.90 – 1.20)	0.645	
	RC_T3	1.34 (1.16 – 1.55)	<0.001		RC/HDL-C_T3	1.37 (1.19 – 1.58)	<0.001	
Gender				0.465				0.544
Female	RC_T1	Ref			RC/HDL-C_T1	Ref		
	RC_T2	1.18 (1.02 – 1.35)	0.023		RC/HDL-C_T2	1.13 (0.98 – 1.30)	0.081	
	RC_T3	1.44 (1.26 – 1.65)	<0.001		RC/HDL-C_T3	1.47 (1.28 – 1.67)	<0.001	
Male	RC_T1	Ref			RC/HDL-C_T1	Ref		
	RC_T2	1.17 (1.01 – 1.35)	0.033		RC/HDL-C_T2	1.07 (0.92 – 1.23)	0.382	

	RC_T3	1.57 (1.36 – 1.81)	<0.001		RC/HDL-C_T3	1.51 (1.32 – 1.74)	<0.001	
Education				0.073				0.236
Elementary school and below	RC_T1	Ref			RC/HDL-C_T1	Ref		
	RC_T2	1.09 (0.97 – 1.22)	0.164		RC/HDL-C_T2	1.04 (0.93 – 1.17)	0.473	
	RC_T3	1.46 (1.30 – 1.64)	<0.001		RC/HDL-C_T3	1.44 (1.29 – 1.61)	<0.001	
Above elementary school	RC_T1	Ref			RC/HDL-C_T1	Ref		
	RC_T2	1.41 (1.17 – 1.71)	<0.001		RC/HDL-C_T2	1.27 (1.05 – 1.55)	0.015	
	RC_T3	1.67 (1.38 – 2.02)	<0.001		RC/HDL-C_T3	1.69 (1.40 – 2.03)	<0.001	
Work				0.402				0.303
No	RC_T1	Ref			RC/HDL-C_T1	Ref		
	RC_T2	1.10 (0.92 – 1.32)	0.308		RC/HDL-C_T2	0.97 (0.81 – 1.16)	0.732	
	RC_T3	1.36 (1.14 – 1.62)	0.001		RC/HDL-C_T3	1.37 (1.15 – 1.62)	<0.001	
Yes	RC_T1	Ref			RC/HDL-C_T1	Ref		
	RC_T2	1.20 (1.07 – 1.35)	0.003		RC/HDL-C_T2	1.16 (1.03 – 1.31)	0.014	
	RC_T3	1.60 (1.42 – 1.80)	<0.001		RC/HDL-C_T3	1.57 (1.40 – 1.76)	<0.001	
Residence				0.059				0.183
City	RC_T1	Ref			RC/HDL-C_T1	Ref		
	RC_T2	1.41 (1.16 – 1.70)	0.001		RC/HDL-C_T2	1.25 (1.03 – 1.51)	0.026	
	RC_T3	1.75 (1.46 – 2.11)	<0.001		RC/HDL-C_T3	1.70 (1.41 – 2.03)	<0.001	
Rural	RC_T1	Ref			RC/HDL-C_T1	Ref		
	RC_T2	1.09 (0.97 – 1.23)	0.135		RC/HDL-C_T2	1.05 (0.93 – 1.18)	0.408	
	RC_T3	1.44 (1.28 – 1.61)	<0.001		RC/HDL-C_T3	1.43 (1.28 – 1.61)	<0.001	
Smoke				0.319				0.029
No	RC_T1	Ref			RC/HDL-C_T1	Ref		
	RC_T2	1.25 (1.10 – 1.42)	0.001		RC/HDL-C_T2	1.23 (1.08 – 1.39)	0.002	
	RC_T3	1.59 (1.40 – 1.80)	<0.001		RC/HDL-C_T3	1.63 (1.44 – 1.84)	<0.001	
Yes	RC_T1				RC/HDL-C_T1	Ref		
	RC_T2	1.08 (0.92 – 1.27)	0.324		RC/HDL-C_T2	0.94 (0.80 – 1.11)	0.479	
	RC_T3	1.41 (1.21 – 1.65)	<0.001		RC/HDL-C_T3	1.33 (1.14 – 1.55)	<0.001	

Drink				0.130				0.120
No	RC_T1	Ref			RC/HDL-C_T1	Ref		
	RC_T2	1.19 (1.04 – 1.35)	0.010		RC/HDL-C_T2	1.10 (0.97 – 1.25)	0.146	
	RC_T3	1.43 (1.26 – 1.62)	<0.001		RC/HDL-C_T3	1.41 (1.24 – 1.59)	<0.001	
Yes	RC_T1	Ref			RC/HDL-C_T1	Ref		
	RC_T2	1.16 (0.99 – 1.36)	0.065		RC/HDL-C_T2	1.11 (0.94 – 1.30)	0.21	
	RC_T3	1.64 (1.41 – 1.92)	<0.001		RC/HDL-C_T3	1.64 (1.41 – 1.91)	<0.001	
Life satisfaction				0.454				0.427
Satisfied	RC_T1	Ref			RC/HDL-C_T1	Ref		
	RC_T2	1.18 (1.06 – 1.32)	0.003		RC/HDL-C_T2	1.11 (0.99 – 1.23)	0.068	
	RC_T3	1.55 (1.39 – 1.72)	<0.001		RC/HDL-C_T3	1.53 (1.38 – 1.70)	<0.001	
Unsatisfied	RC_T1	Ref			RC/HDL-C_T1	Ref		
	RC_T2	1.12 (0.88 – 1.44)	0.357		RC/HDL-C_T2	1.09 (0.84 – 1.40)	0.518	
	RC_T3	1.37 (1.07 – 1.75)	0.011		RC/HDL-C_T3	1.35 (1.07 – 1.72)	0.013	
Antidiabetic drugs				0.143				0.058
No	RC_T1	Ref			RC/HDL-C_T1	Ref		
	RC_T2	1.19 (1.07 – 1.31)	0.001		RC/HDL-C_T2	1.12 (1.01 – 1.24)	0.033	
	RC_T3	1.51 (1.36 – 1.66)	<0.001		RC/HDL-C_T3	1.49 (1.35 – 1.64)	<0.001	
Yes	RC_T1	Ref			RC/HDL-C_T1	Ref		
	RC_T2	0.60 (0.31 – 1.15)	0.125		RC/HDL-C_T2	0.53 (0.28 – 1.03)	0.06	
	RC_T3	1.47 (0.87 – 2.50)	0.151		RC/HDL-C_T3	1.46 (0.86 – 2.48)	0.158	
Lipid-lowering drug				0.135				0.139
No	RC_T1	Ref			RC/HDL-C_T1	Ref		
	RC_T2	1.18 (1.06 – 1.30)	0.001		RC/HDL-C_T2	1.10 (1.00 – 1.22)	0.056	
	RC_T3	1.53 (1.39 – 1.69)	<0.001		RC/HDL-C_T3	1.52 (1.37 – 1.67)	<0.001	
Yes	RC_T1	Ref			RC/HDL-C_T1	Ref		
	RC_T2	0.91 (0.50 – 1.64)	0.75		RC/HDL-C_T2	0.90 (0.49 – 1.64)	0.724	
	RC_T3	0.90 (0.53 – 1.53)	0.706		RC/HDL-C_T3	0.95 (0.56 – 1.62)	0.847	

BMI – body mass index, AC – atherogenic coefficient, AIP – atherogenic index of plasma, LCI – lipoprotein combine index, Non-HDL-C – non-high-density lipoprotein cholesterol, CRI-I – Castelli risk index-I, CRI-II – Castelli risk index-II, RC – remnant cholesterol, RC/HDL-C – remnant cholesterol/high density lipoprotein cholesterol.

Table S11. Subgroup analysis of non-conventional lipid-inflammatory parameters

Subgroup	Variable	HR (95%CI)	*P* value	*P* for interaction	Variable	HR (95%CI)	*P* value	*P* for interaction
Age				0.192				0.214
< 60	AC-CRP_T1	Ref			AIP-CRP_T1	Ref		
	AC-CRP_T2	1.29 (1.13 – 1.47)	<0.001		AIP-CRP_T2	1.20 (1.04 – 1.37)	0.010	
	AC-CRP_T3	1.52 (1.33 – 1.73)	<0.001		AIP-CRP_T3	1.70 (1.49 – 1.94)	<0.001	
≥ 60	AC-CRP_T1	Ref			AIP-CRP_T1	Ref		
	AC-CRP_T2	1.09 (0.94 – 1.27)	0.270		AIP-CRP_T2	1.05 (0.90 – 1.22)	0.565	
	AC-CRP_T3	1.44 (1.24 – 1.67)	<0.001		AIP-CRP_T3	1.41 (1.22 – 1.63)	<0.001	
Gender				0.449				0.689
Female	AC-CRP_T1	Ref			AIP-CRP_T1	Ref		
	AC-CRP_T2	1.20 (1.05 – 1.39)	0.010		AIP-CRP_T2	1.13 (0.98 – 1.30)	0.103	
	AC-CRP_T3	1.55 (1.35 – 1.77)	<0.001		AIP-CRP_T3	1.61 (1.40 – 1.84)	<0.001	
Male	AC-CRP_T1	Ref			AIP-CRP_T1	Ref		
	AC-CRP_T2	1.20 (1.03 – 1.38)	0.015		AIP-CRP_T2	1.12 (0.97 – 1.30)	0.116	
	AC-CRP_T3	1.41 (1.22 – 1.63)	<0.001		AIP-CRP_T3	1.51 (1.31 – 1.73)	<0.001	
Education				0.734				0.522
Elementary school and below	AC-CRP_T1	Ref			AIP-CRP_T1	Ref		
	AC-CRP_T2	1.17 (1.04 – 1.31)	0.010		AIP-CRP_T2	1.11 (0.99 – 1.25)	0.084	
	AC-CRP_T3	1.45 (1.30 – 1.63)	<0.001		AIP-CRP_T3	1.51 (1.35 – 1.69)	<0.001	
Above elementary school	AC-CRP_T1	Ref			AIP-CRP_T1	Ref		
	AC-CRP_T2	1.30 (1.07 – 1.58)	0.008		AIP-CRP_T2	1.19 (0.98 – 1.45)	0.086	
	AC-CRP_T3	1.60 (1.32 – 1.94)	<0.001		AIP-CRP_T3	1.77 (1.47 – 2.13)	<0.001	
Work				0.798				0.493
No	AC-CRP_T1	Ref			AIP-CRP_T1	Ref		
	AC-CRP_T2	1.17 (0.97 – 1.42)	0.105		AIP-CRP_T2	1.02 (0.84 – 1.24)	0.838	
	AC-CRP_T3	1.43 (1.19 – 1.71)	<0.001		AIP-CRP_T3	1.48 (1.24 – 1.77)	<0.001	
Yes	AC-CRP_T1	Ref			AIP-CRP_T1	Ref		

	AC-CRP_T2	1.21 (1.08 – 1.37)	0.001		AIP-CRP_T2	1.18 (1.05 – 1.33)	0.007	
	AC-CRP_T3	1.53 (1.36 – 1.72)	<0.001		AIP-CRP_T3	1.62 (1.44 – 1.81)	<0.001	
Residence				0.764				0.486
City	AC-CRP_T1	Ref			AIP-CRP_T1	Ref		
	AC-CRP_T2	1.26 (1.04 – 1.53)	0.019		AIP-CRP_T2	1.13 (0.93 – 1.38)	0.216	
	AC-CRP_T3	1.57 (1.31 – 1.88)	<0.001		AIP-CRP_T3	1.67 (1.40 – 2.00)	<0.001	
Rural	AC-CRP_T1	Ref			AIP-CRP_T1	Ref		
	AC-CRP_T2	1.19 (1.05 – 1.34)	0.005		AIP-CRP_T2	1.14 (1.01 – 1.28)	0.033	
	AC-CRP_T3	1.46 (1.30 – 1.64)	<0.001		AIP-CRP_T3	1.53 (1.36 – 1.72)	<0.001	
Smoke				0.231				0.388
No	AC-CRP_T1	Ref			AIP-CRP_T1	Ref		
	AC-CRP_T2	1.26 (1.11 – 1.44)	<0.001		AIP-CRP_T2	1.19 (1.04 – 1.35)	0.011	
	AC-CRP_T3	1.59 (1.40 – 1.80)	<0.001		AIP-CRP_T3	1.66 (1.46 – 1.88)	<0.001	
Yes	AC-CRP_T1	Ref			AIP-CRP_T1	Ref		
	AC-CRP_T2	1.11 (0.95 – 1.30)	0.203		AIP-CRP_T2	1.05 (0.90 – 1.24)	0.528	
	AC-CRP_T3	1.34 (1.15 – 1.57)	<0.001		AIP-CRP_T3	1.46 (1.25 – 1.70)	<0.001	
Drink				0.613				0.106
No	AC-CRP_T1	Ref			AIP-CRP_T1	Ref		
	AC-CRP_T2	1.25 (1.09 – 1.42)	0.001		AIP-CRP_T2	1.21 (1.06 – 1.39)	0.004	
	AC-CRP_T3	1.49 (1.31 – 1.70)	<0.001		AIP-CRP_T3	1.56 (1.37 – 1.78)	<0.001	
Yes	AC-CRP_T1	Ref			AIP-CRP_T1	Ref		
	AC-CRP_T2	1.15 (0.98 – 1.34)	0.089		AIP-CRP_T2	1.02 (0.87 – 1.19)	0.827	
	AC-CRP_T3	1.49 (1.28 – 1.73)	<0.001		AIP-CRP_T3	1.60 (1.38 – 1.85)	<0.001	
Life satisfaction				0.97				0.635
Satisfied	AC-CRP_T1	Ref			AIP-CRP_T1	Ref		
	AC-CRP_T2	1.21 (1.08 – 1.35)	0.001		AIP-CRP_T2	1.13 (1.02 – 1.27)	0.025	
	AC-CRP_T3	1.49 (1.34 – 1.66)	<0.001		AIP-CRP_T3	1.60 (1.44 – 1.78)	<0.001	
Unsatisfied	AC-CRP_T1	Ref			AIP-CRP_T1	Ref		
	AC-CRP_T2	1.18 (0.92 – 1.52)	0.198		AIP-CRP_T2	1.14 (0.89 – 1.47)	0.295	

	AC-CRP_T3	1.50 (1.18 – 1.92)	0.001		AIP-CRP_T3	1.48 (1.16 – 1.89)	0.002	
Antidiabetic drugs				0.652				0.105
No	AC-CRP_T1	Ref			AIP-CRP_T1	Ref		
	AC-CRP_T2	1.20 (1.09 – 1.33)	<0.001		AIP-CRP_T2	1.12 (1.01 – 1.24)	0.035	
	AC-CRP_T3	1.49 (1.35 – 1.65)	<0.001		AIP-CRP_T3	1.57 (1.43 – 1.74)	<0.001	
Yes	AC-CRP_T1	Ref			AIP-CRP_T1	Ref		
	AC-CRP_T2	1.17 (0.61 – 2.26)	0.643		AIP-CRP_T2	1.57 (0.79 – 3.12)	0.196	
	AC-CRP_T3	1.17 (0.65 – 2.13)	0.598		AIP-CRP_T3	1.32 (0.69 – 2.52)	0.409	
Lipid-lowering drug				0.286				0.298
No	AC-CRP_T1	Ref			AIP-CRP_T1	Ref		
	AC-CRP_T2	1.22 (1.10 – 1.35)	<0.001		AIP-CRP_T2	1.14 (1.03 – 1.27)	0.010	
	AC-CRP_T3	1.50 (1.35 – 1.65)	<0.001		AIP-CRP_T3	1.58 (1.43 – 1.74)	<0.001	
Yes	AC-CRP_T1	Ref			AIP-CRP_T1	Ref		
	AC-CRP_T2	0.75 (0.39 – 1.45)	0.387		AIP-CRP_T2	0.66 (0.34 – 1.29)	0.223	
	AC-CRP_T3	1.09 (0.61 – 1.96)	0.771		AIP-CRP_T3	1.12 (0.63 – 1.98)	0.699	
Age				0.355				0.533
< 60	CRI-I-CRP_T1	Ref			CRI-II-CRP_T1	Ref		
	CRI-I-CRP_T2	1.28 (1.12 – 1.46)	<0.001		CRI-II-CRP_T2	1.17 (1.03 – 1.34)	0.020	
	CRI-I-CRP_T3	1.49 (1.31 – 1.70)	<0.001		CRI-II-CRP_T3	1.38 (1.21 – 1.57)	<0.001	
≥ 60	CRI-I-CRP_T1	Ref			CRI-II-CRP_T1	Ref		
	CRI-I-CRP_T2	1.12 (0.96 – 1.30)	0.153		CRI-II-CRP_T2	1.10 (0.95 – 1.29)	0.209	
	CRI-I-CRP_T3	1.44 (1.24 – 1.67)	<0.001		CRI-II-CRP_T3	1.43 (1.23 – 1.66)	<0.001	
Gender				0.725				0.922
Female	CRI-I-CRP_T1	Ref			CRI-II-CRP_T1	Ref		
	CRI-I-CRP_T2	1.22 (1.06 – 1.40)	0.006		CRI-II-CRP_T2	1.13 (0.98 – 1.30)	0.088	
	CRI-I-CRP_T3	1.50 (1.31 – 1.72)	<0.001		CRI-II-CRP_T3	1.40 (1.23 – 1.60)	<0.001	
Male	CRI-I-CRP_T1	Ref			CRI-II-CRP_T1	Ref		
	CRI-I-CRP_T2	1.20 (1.04 – 1.39)	0.014		CRI-II-CRP_T2	1.16 (1.00 – 1.34)	0.048	
	CRI-I-CRP_T3	1.42 (1.23 – 1.64)	<0.001		CRI-II-CRP_T3	1.39 (1.21 – 1.61)	<0.001	

Education				0.537				0.872
Elementary school and below	CRI-I-CRP_T1	Ref			CRI-II-CRP_T1	Ref		
	CRI-I-CRP_T2	1.16 (1.04 – 1.31)	0.011		CRI-II-CRP_T2	1.12 (1.00 – 1.26)	0.056	
	CRI-I-CRP_T3	1.43 (1.27 – 1.60)	<0.001		CRI-II-CRP_T3	1.37 (1.22 – 1.54)	<0.001	
Above elementary school	CRI-I-CRP_T1	Ref			CRI-II-CRP_T1	Ref		
	CRI-I-CRP_T2	1.34 (1.10 – 1.63)	0.003		CRI-II-CRP_T2	1.20 (0.99 – 1.46)	0.061	
	CRI-I-CRP_T3	1.61 (1.33 – 1.95)	<0.001		CRI-II-CRP_T3	1.50 (1.24 – 1.81)	<0.001	
Work				0.896				0.610
No	CRI-I-CRP_T1	Ref			CRI-II-CRP_T1	Ref		
	CRI-I-CRP_T2	1.24 (1.02 – 1.51)	0.028		CRI-II-CRP_T2	1.17 (0.97 – 1.42)	0.1	
	CRI-I-CRP_T3	1.54 (1.28 – 1.85)	<0.001		CRI-II-CRP_T3	1.36 (1.13 – 1.63)	0.001	
Yes	CRI-I-CRP_T1	Ref			CRI-II-CRP_T1	Ref		
	CRI-I-CRP_T2	1.20 (1.07 – 1.35)	0.002		CRI-II-CRP_T2	1.13 (1.00 – 1.27)	0.042	
	CRI-I-CRP_T3	1.46 (1.30 – 1.64)	<0.001		CRI-II-CRP_T3	1.44 (1.28 – 1.62)	<0.001	
Residence				0.630				0.955
City	CRI-I-CRP_T1	Ref			CRI-II-CRP_T1	Ref		
	CRI-I-CRP_T2	1.26 (1.04 – 1.53)	0.017		CRI-II-CRP_T2	1.18 (0.97 – 1.42)	0.094	
	CRI-I-CRP_T3	1.58 (1.32 – 1.89)	<0.001		CRI-II-CRP_T3	1.43 (1.20 – 1.71)	<0.001	
Rural	CRI-I-CRP_T1	Ref			CRI-II-CRP_T1	Ref		
	CRI-I-CRP_T2	1.20 (1.06 – 1.35)	0.003		CRI-II-CRP_T2	1.13 (1.01 – 1.28)	0.038	
	CRI-I-CRP_T3	1.43 (1.27 – 1.61)	<0.001		CRI-II-CRP_T3	1.40 (1.24 – 1.57)	<0.001	
Smoke				0.421				0.655
No	CRI-I-CRP_T1	Ref			CRI-II-CRP_T1	Ref		
	CRI-I-CRP_T2	1.25 (1.10 – 1.42)	0.001		CRI-II-CRP_T2	1.18 (1.04 – 1.34)	0.011	
	CRI-I-CRP_T3	1.55 (1.37 – 1.76)	<0.001		CRI-II-CRP_T3	1.44 (1.27 – 1.63)	<0.001	
Yes	CRI-I-CRP_T1	Ref			CRI-II-CRP_T1	Ref		
	CRI-I-CRP_T2	1.14 (0.97 – 1.34)	0.107		CRI-II-CRP_T2	1.08 (0.92 – 1.27)	0.354	
	CRI-I-CRP_T3	1.36 (1.16 – 1.58)	<0.001		CRI-II-CRP_T3	1.35 (1.15 – 1.57)	<0.001	
Drink				0.539				0.309

No	CRI-I-CRP_T1	Ref			CRI-II-CRP_T1	Ref		
	CRI-I-CRP_T2	1.25 (1.10 – 1.42)	0.001		CRI-II-CRP_T2	1.18 (1.04 – 1.35)	0.011	
	CRI-I-CRP_T3	1.46 (1.29 – 1.66)	<0.001		CRI-II-CRP_T3	1.37 (1.21 – 1.56)	<0.001	
Yes	CRI-I-CRP_T1	Ref			CRI-II-CRP_T1	Ref		
	CRI-I-CRP_T2	1.15 (0.98 – 1.35)	0.078		CRI-II-CRP_T2	1.08 (0.93 – 1.27)	0.312	
	CRI-I-CRP_T3	1.49 (1.28 – 1.73)	<0.001		CRI-II-CRP_T3	1.46 (1.25 – 1.69)	<0.001	
Life satisfaction				0.544				0.773
Satisfied	CRI-I-CRP_T1	Ref			CRI-II-CRP_T1	Ref		
	CRI-I-CRP_T2	1.23 (1.11 – 1.38)	<0.001		CRI-II-CRP_T2	1.15 (1.03 – 1.28)	0.014	
	CRI-I-CRP_T3	1.47 (1.32 – 1.64)	<0.001		CRI-II-CRP_T3	1.39 (1.25 – 1.54)	<0.001	
Unsatisfied	CRI-I-CRP_T1	Ref			CRI-II-CRP_T1	Ref		
	CRI-I-CRP_T2	1.09 (0.85 – 1.40)	0.501		CRI-II-CRP_T2	1.13 (0.88 – 1.45)	0.336	
	CRI-I-CRP_T3	1.50 (1.17 – 1.91)	0.001		CRI-II-CRP_T3	1.53 (1.20 – 1.95)	0.001	
Antidiabetic drugs				0.635				0.658
No	CRI-I-CRP_T1	Ref			CRI-II-CRP_T1	Ref		
	CRI-I-CRP_T2	1.21 (1.10 – 1.34)	<0.001		CRI-II-CRP_T2	1.14 (1.03 – 1.27)	0.009	
	CRI-I-CRP_T3	1.48 (1.34 – 1.63)	<0.001		CRI-II-CRP_T3	1.41 (1.28 – 1.56)	<0.001	
Yes	CRI-I-CRP_T1	Ref			CRI-II-CRP_T1	Ref		
	CRI-I-CRP_T2	1.01 (0.51 – 2.00)	0.981		CRI-II-CRP_T2	1.08 (0.56 – 2.06)	0.822	
	CRI-I-CRP_T3	1.13 (0.62 – 2.08)	0.690		CRI-II-CRP_T3	1.15 (0.63 – 2.09)	0.644	
Lipid-lowering drug				0.760				0.541
No	CRI-I-CRP_T1	Ref			CRI-II-CRP_T1	Ref		
	CRI-I-CRP_T2	1.22 (1.10 – 1.35)	<0.001		CRI-II-CRP_T2	1.15 (1.04 – 1.27)	0.006	
	CRI-I-CRP_T3	1.48 (1.34 – 1.63)	<0.001		CRI-II-CRP_T3	1.41 (1.27 – 1.55)	<0.001	
Yes	CRI-I-CRP_T1	Ref			CRI-II-CRP_T1	Ref		
	CRI-I-CRP_T2	1.05 (0.55 – 1.99)	0.887		CRI-II-CRP_T2	0.84 (0.44 – 1.62)	0.607	
	CRI-I-CRP_T3	1.22 (0.67 – 2.23)	0.517		CRI-II-CRP_T3	1.20 (0.67 – 2.13)	0.541	
Age				0.386				0.561

< 60	LCI-CRP_T1	Ref			Non-HDL-C-CRP_T1	Ref		
	LCI-CRP_T2	1.18 (1.03 – 1.35)	0.018		Non-HDL-C-CRP_T2	1.24 (1.08 – 1.41)	0.002	
	LCI-CRP_T3	1.69 (1.48 – 1.92)	<0.001		Non-HDL-C-CRP_T3	1.42 (1.25 – 1.62)	<0.001	
≥ 60	LCI-CRP_T1	Ref			Non-HDL-C-CRP_T1	Ref		
	LCI-CRP_T2	1.13 (0.97 – 1.32)	0.114		Non-HDL-C-CRP_T2	1.11 (0.95 – 1.29)	0.183	
	LCI-CRP_T3	1.46 (1.26 – 1.70)	<0.001		Non-HDL-C-CRP_T3	1.37 (1.18 – 1.59)	<0.001	
Gender				0.95				0.428
Female	LCI-CRP_T1	Ref			Non-HDL-C-CRP_T1	Ref		
	LCI-CRP_T2	1.13 (0.98 – 1.31)	0.086		Non-HDL-C-CRP_T2	1.14 (1.00 – 1.32)	0.058	
	LCI-CRP_T3	1.57 (1.37 – 1.80)	<0.001		Non-HDL-C-CRP_T3	1.42 (1.24 – 1.63)	<0.001	
Male	LCI-CRP_T1	Ref			Non-HDL-C-CRP_T1	Ref		
	LCI-CRP_T2	1.17 (1.01 – 1.35)	0.032		Non-HDL-C-CRP_T2	1.22 (1.06 – 1.41)	0.006	
	LCI-CRP_T3	1.58 (1.37 – 1.81)	<0.001		Non-HDL-C-CRP_T3	1.36 (1.18 – 1.57)	<0.001	
Education				0.385				0.479
Elementary school and below	LCI-CRP_T1	Ref			Non-HDL-C-CRP_T1	Ref		
	LCI-CRP_T2	1.12 (0.99 – 1.26)	0.066		Non-HDL-C-CRP_T2	1.13 (1.00 – 1.27)	0.044	
	LCI-CRP_T3	1.51 (1.34 – 1.69)	<0.001		Non-HDL-C-CRP_T3	1.35 (1.21 – 1.52)	<0.001	
Above elementary school	LCI-CRP_T1	Ref			Non-HDL-C-CRP_T1	Ref		
	LCI-CRP_T2	1.26 (1.04 – 1.54)	0.020		Non-HDL-C-CRP_T2	1.33 (1.10 – 1.61)	0.004	
	LCI-CRP_T3	1.82 (1.51 – 2.19)	<0.001		Non-HDL-C-CRP_T3	1.52 (1.26 – 1.83)	<0.001	
Work				0.728				0.858
No	LCI-CRP_T1	Ref			Non-HDL-C-CRP_T1	Ref		
	LCI-CRP_T2	1.09 (0.89 – 1.32)	0.401		Non-HDL-C-CRP_T2	1.23 (1.01 – 1.49)	0.035	
	LCI-CRP_T3	1.57 (1.31 – 1.88)	<0.001		Non-HDL-C-CRP_T3	1.42 (1.18 – 1.71)	<0.001	
Yes	LCI-CRP_T1	Ref			Non-HDL-C-CRP_T1	Ref		
	LCI-CRP_T2	1.18 (1.05 – 1.33)	0.006		Non-HDL-C-CRP_T2	1.16 (1.03 – 1.31)	0.012	
	LCI-CRP_T3	1.60 (1.43 – 1.80)	<0.001		Non-HDL-C-CRP_T3	1.40 (1.25 – 1.58)	<0.001	
Residence				0.733				0.935
City	LCI-CRP_T1	Ref			Non-HDL-C-CRP_T1	Ref		

	LCI-CRP_T2	1.14 (0.94 – 1.38)	0.194		Non-HDL-C-CRP_T2	1.16 (0.96 – 1.40)	0.118	
	LCI-CRP_T3	1.63 (1.36 – 1.94)	<0.001		Non-HDL-C-CRP_T3	1.40 (1.17 – 1.68)	<0.001	
Rural	LCI-CRP_T1	Ref			Non-HDL-C-CRP_T1	Ref		
	LCI-CRP_T2	1.17 (1.04 – 1.32)	0.010		Non-HDL-C-CRP_T2	1.19 (1.06 – 1.34)	0.004	
	LCI-CRP_T3	1.57 (1.40 – 1.76)	<0.001		Non-HDL-C-CRP_T3	1.40 (1.24 – 1.57)	<0.001	
Smoke				0.631				0.297
No	LCI-CRP_T1	Ref			Non-HDL-C-CRP_T1	Ref		
	LCI-CRP_T2	1.19 (1.04 – 1.36)	0.010		Non-HDL-C-CRP_T2	1.19 (1.05 – 1.36)	0.007	
	LCI-CRP_T3	1.64 (1.45 – 1.87)	<0.001		Non-HDL-C-CRP_T3	1.48 (1.30 – 1.68)	<0.001	
Yes	LCI-CRP_T1	Ref			Non-HDL-C-CRP_T1	Ref		
	LCI-CRP_T2	1.10 (0.94 – 1.29)	0.241		Non-HDL-C-CRP_T2	1.16 (0.99 – 1.36)	0.067	
	LCI-CRP_T3	1.49 (1.28 – 1.74)	<0.001		Non-HDL-C-CRP_T3	1.28 (1.10 – 1.50)	0.002	
Drink				0.993				0.847
No	LCI-CRP_T1	Ref			Non-HDL-C-CRP_T1	Ref		
	LCI-CRP_T2	1.16 (1.01 – 1.32)	0.032		Non-HDL-C-CRP_T2	1.19 (1.04 – 1.35)	0.009	
	LCI-CRP_T3	1.59 (1.40 – 1.80)	<0.001		Non-HDL-C-CRP_T3	1.37 (1.21 – 1.56)	<0.001	
Yes	LCI-CRP_T1	Ref			Non-HDL-C-CRP_T1	Ref		
	LCI-CRP_T2	1.15 (0.98 – 1.35)	0.080		Non-HDL-C-CRP_T2	1.17 (1.00 – 1.37)	0.048	
	LCI-CRP_T3	1.58 (1.36 – 1.84)	<0.001		Non-HDL-C-CRP_T3	1.44 (1.24 – 1.68)	<0.001	
Life satisfaction				0.819				0.788
Satisfied	LCI-CRP_T1	Ref			Non-HDL-C-CRP_T1	Ref		
	LCI-CRP_T2	1.17 (1.04 – 1.30)	0.007		Non-HDL-C-CRP_T2	1.19 (1.06 – 1.32)	0.002	
	LCI-CRP_T3	1.60 (1.44 – 1.78)	<0.001		Non-HDL-C-CRP_T3	1.38 (1.24 – 1.54)	<0.001	
Unsatisfied	LCI-CRP_T1	Ref			Non-HDL-C-CRP_T1	Ref		
	LCI-CRP_T2	1.11 (0.86 – 1.43)	0.410		Non-HDL-C-CRP_T2	1.15 (0.90 – 1.48)	0.267	
	LCI-CRP_T3	1.53 (1.20 – 1.96)	0.001		Non-HDL-C-CRP_T3	1.49 (1.16 – 1.90)	0.002	
Antidiabetic drugs				0.259				0.25
No	LCI-CRP_T1	Ref			Non-HDL-C-CRP_T1	Ref		
	LCI-CRP_T2	1.14 (1.03 – 1.27)	0.011		Non-HDL-C-CRP_T2	1.20 (1.08 – 1.32)	0.001	

	LCI-CRP_T3	1.58 (1.44 – 1.75)	<0.001		Non-HDL-C-CRP_T3	1.41 (1.28 – 1.56)	<0.001	
Yes	LCI-CRP_T1	Ref			Non-HDL-C-CRP_T1	Ref		
	LCI-CRP_T2	1.95 (0.99 – 3.84)	0.053		Non-HDL-C-CRP_T2	0.76 (0.39 – 1.48)	0.422	
	LCI-CRP_T3	1.50 (0.78 – 2.87)	0.221		Non-HDL-C-CRP_T3	0.92 (0.52 – 1.61)	0.770	
Lipid-lowering drug				0.153				0.310
No	LCI-CRP_T1	Ref			Non-HDL-C-CRP_T1	Ref		
	LCI-CRP_T2	1.17 (1.05 – 1.30)	0.003		Non-HDL-C-CRP_T2	1.20 (1.08 – 1.32)	0.001	
	LCI-CRP_T3	1.60 (1.45 – 1.77)	<0.001		Non-HDL-C-CRP_T3	1.40 (1.27 – 1.55)	<0.001	
Yes	LCI-CRP_T1	Ref			Non-HDL-C-CRP_T1	Ref		
	LCI-CRP_T2	0.62 (0.32 – 1.22)	0.169		Non-HDL-C-CRP_T2	0.74 (0.39 – 1.40)	0.359	
	LCI-CRP_T3	0.90 (0.50 – 1.62)	0.722		Non-HDL-C-CRP_T3	1.10 (0.62 – 1.98)	0.739	

Age				0.398				0.505
< 60	RC-CRP_T1	Ref			RC/HDL-C-CRP_T1	Ref		
	RC-CRP_T2	1.25 (1.09 – 1.43)	0.001		RC/HDL-C-CRP_T2	1.20 (1.05 – 1.38)	0.009	
	RC-CRP_T3	1.66 (1.46 – 1.89)	<0.001		RC/HDL-C-CRP_T3	1.61 (1.41 – 1.83)	<0.001	
≥ 60	RC-CRP_T1	Ref			RC/HDL-C-CRP_T1	Ref		
	RC-CRP_T2	1.15 (0.99 – 1.33)	0.076		RC/HDL-C-CRP_T2	1.15 (0.99 – 1.33)	0.074	
	RC-CRP_T3	1.44 (1.24 – 1.66)	<0.001		RC/HDL-C-CRP_T3	1.43 (1.24 – 1.65)	<0.001	
Gender				0.859				0.865
Female	RC-CRP_T1	Ref			RC/HDL-C-CRP_T1	Ref		
	RC-CRP_T2	1.20 (1.04 – 1.38)	0.011		RC/HDL-C-CRP_T2	1.18 (1.02 – 1.35)	0.023	
	RC-CRP_T3	1.58 (1.38 – 1.81)	<0.001		RC/HDL-C-CRP_T3	1.56 (1.37 – 1.78)	<0.001	
Male	RC-CRP_T1	Ref			RC/HDL-C-CRP_T1	Ref		
	RC-CRP_T2	1.20 (1.04 – 1.39)	0.015		RC/HDL-C-CRP_T2	1.17 (1.01 – 1.35)	0.038	
	RC-CRP_T3	1.52 (1.32 – 1.75)	<0.001		RC/HDL-C-CRP_T3	1.49 (1.29 – 1.72)	<0.001	
Education				0.139				0.095
Elementary school and below	RC-CRP_T1	Ref			RC/HDL-C-CRP_T1	Ref		
	RC-CRP_T2	1.14 (1.01 – 1.28)	0.035		RC/HDL-C-CRP_T2	1.10 (0.98 – 1.23)	0.119	
	RC-CRP_T3	1.47 (1.31 – 1.64)	<0.001		RC/HDL-C-CRP_T3	1.45 (1.30 – 1.63)	<0.001	

Above elementary school	RC-CRP_T1	Ref			RC/HDL-C-CRP_T1	Ref		
	RC-CRP_T2	1.38 (1.14 – 1.68)	0.001		RC/HDL-C-CRP_T2	1.41 (1.15 – 1.71)	0.001	
	RC-CRP_T3	1.86 (1.53 – 2.24)	<0.001		RC/HDL-C-CRP_T3	1.81 (1.50 – 2.19)	<0.001	
Work				0.583				0.91
No	RC-CRP_T1	Ref			RC/HDL-C-CRP_T1	Ref		
	RC-CRP_T2	1.11 (0.92 – 1.35)	0.272		RC/HDL-C-CRP_T2	1.16 (0.96 – 1.41)	0.117	
	RC-CRP_T3	1.52 (1.28 – 1.82)	<0.001		RC/HDL-C-CRP_T3	1.49 (1.25 – 1.78)	<0.001	
Yes	RC-CRP_T1	Ref			RC/HDL-C-CRP_T1	Ref		
	RC-CRP_T2	1.25 (1.11 – 1.40)	<0.001		RC/HDL-C-CRP_T2	1.18 (1.05 – 1.33)	0.006	
	RC-CRP_T3	1.59 (1.41 – 1.78)	<0.001		RC/HDL-C-CRP_T3	1.57 (1.40 – 1.76)	<0.001	
Residence				0.341				0.193
City	RC-CRP_T1	Ref			RC/HDL-C-CRP_T1	Ref		
	RC-CRP_T2	1.28 (1.05 – 1.55)	0.014		RC/HDL-C-CRP_T2	1.28 (1.05 – 1.56)	0.014	
	RC-CRP_T3	1.74 (1.45 – 2.09)	<0.001		RC/HDL-C-CRP_T3	1.75 (1.46 – 2.11)	<0.001	
Rural	RC-CRP_T1	Ref			RC/HDL-C-CRP_T1	Ref		
	RC-CRP_T2	1.18 (1.05 – 1.33)	0.007		RC/HDL-C-CRP_T2	1.14 (1.01 – 1.28)	0.027	
	RC-CRP_T3	1.50 (1.33 – 1.68)	<0.001		RC/HDL-C-CRP_T3	1.46 (1.30 – 1.63)	<0.001	
Smoke				0.062				0.149
No	RC-CRP_T1	Ref			RC/HDL-C-CRP_T1	Ref		
	RC-CRP_T2	1.31 (1.15 – 1.49)	<0.001		RC/HDL-C-CRP_T2	1.25 (1.10 – 1.43)	0.001	
	RC-CRP_T3	1.70 (1.50 – 1.92)	<0.001		RC/HDL-C-CRP_T3	1.65 (1.46 – 1.87)	<0.001	
Yes	RC-CRP_T1	Ref			RC/HDL-C-CRP_T1	Ref		
	RC-CRP_T2	1.05 (0.89 – 1.23)	0.565		RC/HDL-C-CRP_T2	1.06 (0.90 – 1.24)	0.499	
	RC-CRP_T3	1.37 (1.17 – 1.60)	<0.001		RC/HDL-C-CRP_T3	1.37 (1.17 – 1.60)	<0.001	
Drink				0.970				0.949
No	RC-CRP_T1	Ref			RC/HDL-C-CRP_T1	Ref		
	RC-CRP_T2	1.21 (1.06 – 1.39)	0.004		RC/HDL-C-CRP_T2	1.16 (1.02 – 1.32)	0.026	
	RC-CRP_T3	1.57 (1.38 – 1.78)	<0.001		RC/HDL-C-CRP_T3	1.54 (1.36 – 1.74)	<0.001	
Yes	RC-CRP_T1	Ref			RC/HDL-C-CRP_T1	Ref		

	RC-CRP_T2	1.19 (1.02 – 1.39)	0.032		RC/HDL-C-CRP_T2	1.20 (1.02 – 1.40)	0.024	
	RC-CRP_T3	1.56 (1.33 – 1.82)	<0.001		RC/HDL-C-CRP_T3	1.53 (1.31 – 1.79)	<0.001	
Life satisfaction				0.627				0.321
Satisfied	RC-CRP_T1	Ref			RC/HDL-C-CRP_T1	Ref		
	RC-CRP_T2	1.22 (1.10 – 1.37)	<0.001		RC/HDL-C-CRP_T2	1.21 (1.08 – 1.35)	0.001	
	RC-CRP_T3	1.56 (1.40 – 1.74)	<0.001		RC/HDL-C-CRP_T3	1.55 (1.39 – 1.72)	<0.001	
Unsatisfied	RC-CRP_T1	Ref			RC/HDL-C-CRP_T1	Ref		
	RC-CRP_T2	1.09 (0.84 – 1.40)	0.521		RC/HDL-C-CRP_T2	1.00 (0.77 – 1.29)	0.993	
	RC-CRP_T3	1.56 (1.22 – 1.99)	<0.001		RC/HDL-C-CRP_T3	1.51 (1.19 – 1.91)	0.001	
Antidiabetic drugs				0.222				0.646
No	RC-CRP_T1	Ref			RC/HDL-C-CRP_T1	Ref		
	RC-CRP_T2	1.21 (1.09 – 1.34)	<0.001		RC/HDL-C-CRP_T2	1.18 (1.07 – 1.31)	0.001	
	RC-CRP_T3	1.58 (1.43 – 1.74)	<0.001		RC/HDL-C-CRP_T3	1.54 (1.40 – 1.70)	<0.001	
Yes	RC-CRP_T1	Ref			RC/HDL-C-CRP_T1	Ref		
	RC-CRP_T2	0.92 (0.48 – 1.76)	0.802		RC/HDL-C-CRP_T2	0.83 (0.43 – 1.60)	0.580	
	RC-CRP_T3	1.01 (0.59 – 1.74)	0.961		RC/HDL-C-CRP_T3	1.09 (0.62 – 1.91)	0.762	
Lipid-lowering drug				0.246				0.406
No	RC-CRP_T1	Ref			RC/HDL-C-CRP_T1	Ref		
	RC-CRP_T2	1.21 (1.09 – 1.34)	<0.001		RC/HDL-C-CRP_T2	1.18 (1.07 – 1.31)	0.001	
	RC-CRP_T3	1.58 (1.43 – 1.74)	<0.001		RC/HDL-C-CRP_T3	1.55 (1.40 – 1.71)	<0.001	
Yes	RC-CRP_T1	Ref			RC/HDL-C-CRP_T1	Ref		
	RC-CRP_T2	0.88 (0.47 – 1.63)	0.684		RC/HDL-C-CRP_T2	0.79 (0.41 – 1.52)	0.484	
	RC-CRP_T3	0.94 (0.52 – 1.69)	0.832		RC/HDL-C-CRP_T3	1.04 (0.60 – 1.83)	0.880	

BMI – body mass index, AC – atherogenic coefficient, AIP – atherogenic index of plasma, LCI – lipoprotein combine index, Non-HDL-C – non-high-density lipoprotein cholesterol, CRI-I – Castelli risk index-I, CRI-II – Castelli risk index-II, RC – remnant cholesterol, RC/HDL-C – remnant cholesterol/high density lipoprotein cholesterol, CRP – C-reactive protein.

Table S12. Associations between non-conventional lipid and lipid-inflammatory parameters and hypertension risk after exclusion of individuals with cardiovascular disease

Variable	Event, n(%)	Crude Model		Model 1		Model 2		
		HR (95%CI)	*P* value	HR (95%CI)	*P* value	HR (95%CI)	*P* value	Adjusted *P*
AC-CRP								
T1	649 (30.4)	Ref		Ref		Ref		
T2	733 (35)	1.19 (1.07 – 1.32)	0.001	1.16 (1.04 – 1.29)	0.006	1.17 (1.06 – 1.30)	0.003	0.003
T3	875 (42.4)	1.51 (1.37 – 1.68)	<0.001	1.46 (1.32 – 1.62)	<0.001	1.49 (1.34 – 1.65)	<0.001	<0.001
P for trend			<0.001		<0.001		<0.001	<0.001
AIP-CRP								
T1	665 (30.9)	Ref		Ref		Ref		
T2	700 (33.4)	1.09 (0.98 – 1.21)	0.129	1.09 (0.98 – 1.22)	0.094	1.11 (1 – 1.23)	0.056	0.056
T3	892 (43.5)	1.53 (1.38 – 1.69)	<0.001	1.52 (1.37 – 1.68)	<0.001	1.55 (1.4 – 1.72)	<0.001	<0.001
P for trend			<0.001		<0.001		<0.001	<0.001
CRI-I-CRP								
T1	647 (30.3)	Ref		Ref		Ref		
T2	741 (35.5)	1.21 (1.09 – 1.34)	<0.001	1.18 (1.07 – 1.32)	0.002	1.19 (1.07 – 1.33)	0.001	0.001
T3	869 (42)	1.51 (1.36 – 1.67)	<0.001	1.45 (1.31 – 1.61)	<0.001	1.47 (1.33 – 1.63)	<0.001	<0.001
P for trend			<0.001		<0.001		<0.001	<0.001
CRI-II-CRP								
T1	665 (31.2)	Ref		Ref		Ref		
T2	731 (35)	1.15 (1.04 – 1.28)	0.008	1.12 (1.01 – 1.25)	0.033	1.13 (1.02 – 1.26)	0.019	0.019
T3	861 (41.6)	1.44 (1.30 – 1.59)	<0.001	1.38 (1.24 – 1.52)	<0.001	1.40 (1.27 – 1.55)	<0.001	<0.001
P for trend			<0.001		<0.001		<0.001	<0.001
LCI-CRP								
T1	639 (29.8)	Ref		Ref		Ref		
T2	714 (34.2)	1.18 (1.06 – 1.31)	0.003	1.14 (1.03 – 1.27)	0.013	1.16 (1.04 – 1.29)	0.007	0.007
T3	904 (43.7)	1.60 (1.45 – 1.77)	<0.001	1.56 (1.41 – 1.73)	<0.001	1.59 (1.43 – 1.76)	<0.001	<0.001
P for trend			<0.001		<0.001		<0.001	<0.001
Non-HDL-C-CRP								
T1	648 (30.4)	Ref		Ref		Ref		

T2	749 (35.8)	1.22 (1.10 – 1.35)	<0.001	1.17 (1.05 – 1.30)	0.003	1.18 (1.06 – 1.32)	0.002	0.002
T3	860 (41.6)	1.47 (1.33 – 1.63)	<0.001	1.4 (1.26 – 1.55)	<0.001	1.41 (1.27 – 1.57)	<0.001	<0.001
P for trend			<0.001		<0.001		<0.001	<0.001
RC-CRP								
T1	628 (29.5)	Ref		Ref		Ref		
T2	734 (35)	1.22 (1.10 – 1.36)	<0.001	1.20 (1.08 – 1.34)	0.001	1.21 (1.08 – 1.34)	0.001	0.001
T3	895 (43.2)	1.60 (1.44 – 1.77)	<0.001	1.57 (1.42 – 1.74)	<0.001	1.58 (1.43 – 1.75)	<0.001	<0.001
P for trend			<0.001		<0.001		<0.001	<0.001
RC/HDL-C-CRP								
T1	648 (30.5)	Ref		Ref		Ref		
T2	729 (34.6)	1.16 (1.05 – 1.29)	0.005	1.15 (1.04 – 1.28)	0.008	1.16 (1.04 – 1.29)	0.006	0.006
T3	880 (42.7)	1.52 (1.38 – 1.69)	<0.001	1.51 (1.37 – 1.67)	<0.001	1.53 (1.38 – 1.70)	<0.001	<0.001
P for trend			<0.001		<0.001		<0.001	<0.001
AC								
T1	659 (31.1)	Ref		Ref		Ref		
T2	744 (35.2)	1.16 (1.04 – 1.29)	0.006	1.19 (1.07 – 1.32)	0.001	1.22 (1.09 – 1.35)	<0.001	<0.001
T3	854 (41.4)	1.42 (1.29 – 1.58)	<0.001	1.45 (1.31 – 1.61)	<0.001	1.51 (1.36 – 1.67)	<0.001	<0.001
P for trend			<0.001		<0.001		<0.001	<0.001
AIP								
T1	668 (31.3)	Ref		Ref		Ref		
T2	716 (33.8)	1.08 (0.97 – 1.20)	0.157	1.10 (0.99 – 1.22)	0.079	1.11 (1.00 – 1.24)	0.048	0.048
T3	873 (42.8)	1.47 (1.33 – 1.63)	<0.001	1.54 (1.39 – 1.70)	<0.001	1.58 (1.43 – 1.75)	<0.001	<0.001
P for trend			<0.001		<0.001		<0.001	<0.001
CRI-I								
T1	659 (31.1)	Ref		Ref		Ref		
T2	744 (35.2)	1.16 (1.04 – 1.29)	0.006	1.19 (1.07 – 1.32)	0.001	1.22 (1.09 – 1.35)	<0.001	<0.001
T3	854 (41.4)	1.42 (1.29 – 1.58)	<0.001	1.45 (1.31 – 1.61)	<0.001	1.51 (1.36 – 1.67)	<0.001	<0.001
P for trend			<0.001		<0.001		<0.001	<0.001
CRI-II								

T1	675 (31.9)	Ref		Ref		Ref		
T2	759 (35.9)	1.15 (1.04 – 1.27)	0.009	1.16 (1.05 – 1.29)	0.004	1.19 (1.07 – 1.32)	0.001	0.001
T3	823 (39.8)	1.31 (1.19 – 1.45)	<0.001	1.31 (1.18 – 1.45)	<0.001	1.35 (1.22 – 1.50)	<0.001	<0.001
P for trend			<0.001		<0.001		<0.001	<0.001
LCI								
T1	654 (30.8)	Ref		Ref		Ref		
T2	716 (34)	1.13 (1.01 – 1.25)	0.029	1.14 (1.02 – 1.26)	0.019	1.16 (1.04 – 1.29)	0.007	0.007
T3	887 (43)	1.52 (1.37 – 1.68)	<0.001	1.55 (1.4 – 1.71)	<0.001	1.58 (1.43 – 1.76)	<0.001	<0.001
P for trend			<0.001		<0.001		<0.001	<0.001
Non-HDL-C								
T1	660 (31.5)	Ref		Ref		Ref		
T2	749 (35.2)	1.15 (1.04 – 1.28)	0.007	1.15 (1.04 – 1.28)	0.008	1.17 (1.05 – 1.30)	0.003	0.003
T3	848 (41)	1.39 (1.25 – 1.54)	<0.001	1.37 (1.24 – 1.52)	<0.001	1.40 (1.26 – 1.55)	<0.001	<0.001
P for trend			<0.001		<0.001		<0.001	<0.001
RC								
T1	642 (30.9)	Ref		Ref		Ref		
T2	751 (34.6)	1.14 (1.02 – 1.26)	0.016	1.13 (1.02 – 1.26)	0.019	1.14 (1.02 – 1.26)	0.017	0.017
T3	864 (42.2)	1.47 (1.33 – 1.63)	<0.001	1.51 (1.36 – 1.67)	<0.001	1.52 (1.37 – 1.68)	<0.001	<0.001
P for trend			<0.001		<0.001		<0.001	<0.001
RC/HDL-C								
T1	676 (31.9)	Ref		Ref		Ref		
T2	712 (33.7)	1.06 (0.95 – 1.17)	0.319	1.06 (0.96 – 1.18)	0.251	1.07 (0.97 – 1.19)	0.189	0.189
T3	869 (42.1)	1.41 (1.28 – 1.56)	<0.001	1.46 (1.32 – 1.62)	<0.001	1.49 (1.34 – 1.64)	<0.001	<0.001
P for trend			<0.001		<0.001		<0.001	<0.001

Model 1: adjusted for age and gender.

Model 2: adjusted for Model 1 + ethnicity, marital status, educational level, residential location, employment status, medical insurance coverage, smoking and drinking history, sleep duration, life satisfaction.

AC – atherogenic coefficient, AIP – atherogenic index of plasma, LCI – lipoprotein combine index, Non-HDL-C – non-high-density lipoprotein cholesterol, CRI-I – Castelli risk index-I, CRI-II – Castelli risk index-II, RC – remnant cholesterol, RC/HDL-C – remnant cholesterol/high density lipoprotein cholesterol, CRP – C-reactive protein.

Table S13. Associations between non-conventional lipid and lipid-inflammatory parameters and hypertension risk after exclusion of individuals with hypertension at Wave 2

Variable	Event – n(%)	Crude Model		Model 1		Model 2		
		HR (95%CI)	*P* value	HR (95%CI)	*P* value	HR (95%CI)	*P* value	Adjusted *P*
AC-CRP								
T1	609 (27.7)	Ref		Ref		Ref		
T2	743 (33.7)	1.27 (1.14 – 1.41)	<0.001	1.24 (1.11 – 1.38)	<0.001	1.25 (1.12 – 1.39)	<0.001	<0.001
T3	830 (38.9)	1.50 (1.35 – 1.67)	<0.001	1.45 (1.30 – 1.61)	<0.001	1.47 (1.33 – 1.64)	<0.001	<0.001
P for trend			<0.001		<0.001		<0.001	<0.001
AIP-CRP								
T1	620 (28.1)	Ref		Ref		Ref		
T2	696 (31.7)	1.14 (1.03 – 1.27)	0.015	1.15 (1.03 – 1.28)	0.011	1.17 (1.05 – 1.30)	0.005	0.005
T3	866 (40.5)	1.55 (1.40 – 1.72)	<0.001	1.54 (1.39 – 1.70)	<0.001	1.58 (1.42 – 1.75)	<0.001	<0.001
P for trend			<0.001		<0.001		<0.001	<0.001
CRI-I-CRP								
T1	611 (27.8)	Ref		Ref		Ref		
T2	748 (33.9)	1.27 (1.14 – 1.41)	<0.001	1.24 (1.12 – 1.39)	<0.001	1.25 (1.13 – 1.40)	<0.001	<0.001
T3	823 (38.5)	1.48 (1.34 – 1.65)	<0.001	1.43 (1.29 – 1.59)	<0.001	1.45 (1.3 – 1.61)	<0.001	<0.001
P for trend			<0.001		<0.001		<0.001	<0.001
CRI-II-CRP								
T1	627 (28.6)	Ref		Ref		Ref		
T2	728 (33.1)	1.20 (1.08 – 1.33)	0.001	1.17 (1.05 – 1.30)	0.005	1.18 (1.06 – 1.31)	0.002	0.002
T3	827 (38.6)	1.44 (1.29 – 1.59)	<0.001	1.38 (1.24 – 1.53)	<0.001	1.40 (1.26 – 1.56)	<0.001	<0.001
P for trend			<0.001		<0.001		<0.001	<0.001
LCI-CRP								
T1	598 (27.2)	Ref		Ref		Ref		
T2	715 (32.4)	1.23 (1.10 – 1.37)	<0.001	1.19 (1.07 – 1.33)	0.001	1.20 (1.08 – 1.34)	0.001	0.001
T3	869 (40.6)	1.62 (1.46 – 1.79)	<0.001	1.57 (1.41 – 1.74)	<0.001	1.60 (1.44 – 1.78)	<0.001	<0.001
P for trend			<0.001		<0.001		<0.001	<0.001

Non-HDL-C-CRP								
T1	622 (28.2)	Ref		Ref		Ref		
T2	739 (33.7)	1.24 (1.11 – 1.37)	<0.001	1.19 (1.07 – 1.32)	0.002	1.20 (1.08 – 1.34)	0.001	0.001
T3	821 (38.3)	1.44 (1.3 – 1.60)	<0.001	1.36 (1.23 – 1.51)	<0.001	1.38 (1.24 – 1.53)	<0.001	<0.001
P for trend			<0.001		<0.001		<0.001	<0.001
RC-CRP								
T1	606 (27.5)	Ref		Ref		Ref		
T2	724 (33)	1.24 (1.11 – 1.38)	<0.001	1.22 (1.09 – 1.36)	<0.001	1.22 (1.10 – 1.36)	<0.001	<0.001
T3	852 (39.8)	1.56 (1.40 – 1.73)	<0.001	1.53 (1.38 – 1.70)	<0.001	1.55 (1.39 – 1.72)	<0.001	<0.001
P for trend			<0.001		<0.001		<0.001	<0.001
RC/HDL-C-CRP								
T1	620 (28.1)	Ref		Ref		Ref		
T2	721 (32.8)	1.20 (1.08 – 1.34)	0.001	1.19 (1.07 – 1.33)	0.001	1.20 (1.08 – 1.34)	0.001	0.001
T3	841 (39.4)	1.50 (1.35 – 1.66)	<0.001	1.48 (1.34 – 1.65)	<0.001	1.51 (1.36 – 1.68)	<0.001	<0.001
P for trend			<0.001		<0.001		<0.001	<0.001
AC								
T1	616 (28.1)	Ref		Ref		Ref		
T2	731 (33.3)	1.23 (1.10 – 1.37)	<0.001	1.26 (1.13 – 1.40)	<0.001	1.29 (1.16 – 1.43)	<0.001	<0.001
T3	835 (38.8)	1.49 (1.34 – 1.65)	<0.001	1.52 (1.37 – 1.68)	<0.001	1.58 (1.42 – 1.76)	<0.001	<0.001
P for trend			<0.001		<0.001		<0.001	<0.001
AIP								
T1	623 (28.4)	Ref		Ref		Ref		
T2	703 (31.9)	1.14 (1.02 – 1.27)	0.018	1.16 (1.04 – 1.29)	0.007	1.18 (1.05 – 1.31)	0.003	0.003
T3	856 (39.9)	1.52 (1.37 – 1.68)	<0.001	1.58 (1.42 – 1.75)	<0.001	1.63 (1.46 – 1.80)	<0.001	<0.001
P for trend			<0.001		<0.001		<0.001	<0.001
CRI-I								
T1	616 (28.1)	Ref		Ref		Ref		
T2	731 (33.3)	1.23 (1.10 – 1.37)	<0.001	1.26 (1.13 – 1.40)	<0.001	1.29 (1.16 – 1.43)	<0.001	<0.001
T3	835 (38.8)	1.49 (1.34 – 1.65)	<0.001	1.52 (1.37 – 1.68)	<0.001	1.58 (1.42 – 1.76)	<0.001	<0.001

P for trend			<0.001		<0.001		<0.001	<0.001
CRI-II								
T1	632 (28.9)	Ref		Ref		Ref		
T2	741 (33.8)	1.20 (1.08 – 1.34)	0.001	1.22 (1.09 – 1.35)	<0.001	1.25 (1.12 – 1.39)	<0.001	<0.001
T3	809 (37.5)	1.37 (1.24 – 1.53)	<0.001	1.37 (1.24 – 1.52)	<0.001	1.42 (1.28 – 1.58)	<0.001	<0.001
P for trend			<0.001		<0.001		<0.001	<0.001
LCI								
T1	619 (28.1)	Ref		Ref		Ref		
T2	704 (32)	1.17 (1.05 – 1.30)	0.005	1.17 (1.05 – 1.30)	0.004	1.19 (1.07 – 1.33)	0.001	0.001
T3	859 (40.1)	1.54 (1.39 – 1.70)	<0.001	1.56 (1.41 – 1.73)	<0.001	1.61 (1.45 – 1.78)	<0.001	<0.001
P for trend			<0.001		<0.001		<0.001	<0.001
Non-HDL-C								
T1	635 (29)	Ref		Ref		Ref		
T2	725 (33.1)	1.18 (1.06 – 1.31)	0.003	1.17 (1.06 – 1.31)	0.003	1.20 (1.07 – 1.33)	0.001	0.001
T3	822 (38.1)	1.39 (1.26 – 1.55)	<0.001	1.38 (1.24 – 1.53)	<0.001	1.40 (1.26 – 1.56)	<0.001	<0.001
P for trend			<0.001		<0.001		<0.001	<0.001
RC								
T1	621 (28.7)	Ref		Ref		Ref		
T2	736 (32.6)	1.15 (1.04 – 1.29)	0.008	1.16 (1.04 – 1.29)	0.008	1.16 (1.04 – 1.29)	0.006	0.006
T3	825 (38.9)	1.44 (1.30 – 1.60)	<0.001	1.48 (1.33 – 1.64)	<0.001	1.49 (1.34 – 1.66)	<0.001	<0.001
P for trend			<0.001		<0.001		<0.001	<0.001
RC/HDL-C								
T1	649 (29.5)	Ref		Ref		Ref		
T2	690 (31.5)	1.07 (0.96 – 1.19)	0.198	1.09 (0.98 – 1.21)	0.129	1.10 (0.99 – 1.22)	0.087	0.087
T3	843 (39.2)	1.41 (1.27 – 1.56)	<0.001	1.46 (1.32 – 1.62)	<0.001	1.49 (1.35 – 1.66)	<0.001	<0.001
P for trend			<0.001		<0.001		<0.001	<0.001

Model 1: adjusted for age and gender.

Model 2: adjusted for Model 1 + ethnicity, marital status, educational level, residential location, employment status, medical insurance coverage, smoking and drinking history, sleep duration, life satisfaction.

AC – atherogenic coefficient, AIP – atherogenic index of plasma, LCI – lipoprotein combine index, Non-HDL-C – non-high-density lipoprotein cholesterol, CRI-I – Castelli risk index-I, CRI-II – Castelli risk index-II,

RC – remnant cholesterol, RC/HDL-C – remnant cholesterol/high density lipoprotein cholesterol, CRP – C-reactive protein.

Table S14. Associations between non-conventional lipid and lipid-inflammatory parameters and hypertension risk after exclusion of individuals with missing covariates

Variable	Event, n(%)	Crude Model		Model 1		Model 2		
		**HR (95%CI)**	***P* value**	**HR (95%CI)**	***P* value**	**HR (95%CI)**	***P* value**	**Adjusted *P***
AC-CRP								
T1	561 (30.7)	Ref		Ref		Ref		
T2	633 (34.3)	1.14 (1.02 – 1.28)	0.025	1.11 (0.99 – 1.24)	0.07	1.12 (1.00 – 1.26)	0.047	0.047
T3	779 (42.7)	1.51 (1.35 – 1.68)	<0.001	1.46 (1.31 – 1.62)	<0.001	1.48 (1.33 – 1.65)	<0.001	<0.001
P for trend			<0.001		<0.001		<0.001	<0.001
AIP-CRP								
T1	552 (30.2)	Ref		Ref		Ref		
T2	609 (33.1)	1.11 (0.99 – 1.24)	0.086	1.11 (0.99 – 1.25)	0.074	1.13 (1.00 – 1.26)	0.045	0.045
T3	812 (44.3)	1.6 (1.44 – 1.79)	<0.001	1.59 (1.43 – 1.78)	<0.001	1.64 (1.47 – 1.83)	<0.001	<0.001
P for trend			<0.001		<0.001		<0.001	<0.001
CRI-I-CRP								
T1	558 (30.5)	Ref		Ref		Ref		
T2	639 (34.7)	1.17 (1.04 – 1.31)	0.008	1.14 (1.02 – 1.28)	0.023	1.15 (1.03 – 1.29)	0.017	0.017
T3	776 (42.5)	1.51 (1.36 – 1.69)	<0.001	1.46 (1.31 – 1.63)	<0.001	1.48 (1.33 – 1.65)	<0.001	<0.001
P for trend			<0.001		<0.001		<0.001	<0.001
CRI-II-CRP								
T1	576 (31.4)	Ref		Ref		Ref		
T2	634 (34.4)	1.12 (1.00 – 1.25)	0.057	1.08 (0.97 – 1.21)	0.165	1.09 (0.98 – 1.23)	0.116	0.116
T3	763 (41.9)	1.43 (1.28 – 1.59)	<0.001	1.38 (1.23 – 1.53)	<0.001	1.40 (1.25 – 1.56)	<0.001	<0.001
P for trend			<0.001		<0.001		<0.001	<0.001
LCI-CRP								
T1	549 (30.3)	Ref		Ref		Ref		
T2	609 (32.7)	1.09 (0.97 – 1.22)	0.138	1.06 (0.95 – 1.19)	0.312	1.07 (0.96 – 1.20)	0.232	0.232

T3	815 (44.6)	1.61 (1.44 – 1.79)	<0.001	1.56 (1.40 – 1.74)	<0.001	1.59 (1.43 – 1.78)	<0.001	<0.001
P for trend			<0.001		<0.001		<0.001	<0.001
Non-HDL-C-CRP								
T1	571 (31.1)	Ref		Ref		Ref		
T2	641 (34.9)	1.14 (1.02 – 1.28)	0.019	1.10 (0.99 – 1.24)	0.084	1.11 (0.99 – 1.25)	0.063	0.063
T3	761 (41.8)	1.44 (1.29 – 1.60)	<0.001	1.37 (1.23 – 1.53)	<0.001	1.38 (1.24 – 1.54)	<0.001	<0.001
P for trend			<0.001		<0.001		<0.001	<0.001
RC-CRP								
T1	549 (30)	Ref		Ref		Ref		
T2	628 (34.1)	1.16 (1.03 – 1.30)	0.012	1.14 (1.02 – 1.28)	0.024	1.15 (1.02 – 1.29)	0.02	0.02
T3	796 (43.7)	1.59 (1.43 – 1.77)	<0.001	1.56 (1.4 – 1.74)	<0.001	1.58 (1.41 – 1.76)	<0.001	<0.001
P for trend			<0.001		<0.001		<0.001	<0.001
RC/HDL-C-CRP								
T1	562 (30.6)	Ref		Ref		Ref		
T2	613 (33.5)	1.11 (0.99 – 1.24)	0.082	1.10 (0.98 – 1.23)	0.103	1.11 (0.99 – 1.24)	0.078	0.078
T3	798 (43.6)	1.55 (1.39 – 1.73)	<0.001	1.54 (1.38 – 1.72)	<0.001	1.57 (1.40 – 1.75)	<0.001	<0.001
P for trend			<0.001		<0.001		<0.001	<0.001
AC								
T1	549 (30.3)	Ref		Ref		Ref		
T2	642 (35)	1.19 (1.06 – 1.33)	0.003	1.21 (1.08 – 1.36)	0.001	1.24 (1.11 – 1.39)	<0.001	<0.001
T3	782 (42.2)	1.51 (1.35 – 1.69)	<0.001	1.53 (1.37 – 1.71)	<0.001	1.60 (1.43 – 1.78)	<0.001	<0.001
P for trend			<0.001		<0.001		<0.001	<0.001
AIP								
T1	557 (30.6)	Ref		Ref		Ref		
T2	625 (34.2)	1.13 (1.01 – 1.27)	0.03	1.16 (1.03 – 1.30)	0.012	1.18 (1.05 – 1.32)	0.006	0.006
T3	791 (42.8)	1.52 (1.37 – 1.70)	<0.001	1.58 (1.42 – 1.76)	<0.001	1.63 (1.46 – 1.82)	<0.001	<0.001
P for trend			<0.001		<0.001		<0.001	<0.001
CRI-I								
T1	558 (30.5)	Ref		Ref		Ref		

T2	639 (34.7)	1.17 (1.04 – 1.31)	0.008	1.14 (1.02 – 1.28)	0.023	1.24 (1.11 – 1.39)	<0.001	<0.001
T3	776 (42.5)	1.51 (1.36 – 1.69)	<0.001	1.46 (1.31 – 1.63)	<0.001	1.60 (1.43 – 1.78)	<0.001	<0.001
P for trend			<0.001		<0.001		<0.001	<0.001
CRI-II								
T1	576 (31.4)	Ref		Ref		Ref		
T2	634 (34.4)	1.12 (1.00 – 1.25)	0.057	1.08 (0.97 – 1.21)	0.165	1.23 (1.10 – 1.38)	<0.001	<0.001
T3	763 (41.9)	1.43 (1.28 – 1.59)	<0.001	1.38 (1.23 – 1.53)	<0.001	1.43 (1.28 – 1.59)	<0.001	<0.001
P for trend			<0.001		<0.001		<0.001	<0.001
LCI								
T1	549 (30.3)	Ref		Ref		Ref		
T2	609 (32.7)	1.09 (0.97 – 1.22)	0.138	1.06 (0.95 – 1.19)	0.312	1.18 (1.05 – 1.32)	0.005	0.005
T3	815 (44.6)	1.61 (1.44 – 1.79)	<0.001	1.56 (1.40 – 1.74)	<0.001	1.60 (1.43 – 1.79)	<0.001	<0.001
P for trend			<0.001		<0.001		<0.001	<0.001
Non-HDL-C								
T1	571 (31.1)	Ref		Ref		Ref		
T2	641 (34.9)	1.14 (1.02 – 1.28)	0.019	1.10 (0.99 – 1.24)	0.084	1.16 (1.04 – 1.30)	0.008	0.008
T3	761 (41.8)	1.44 (1.29 – 1.60)	<0.001	1.37 (1.23 – 1.53)	<0.001	1.37 (1.22 – 1.53)	<0.001	<0.001
P for trend			<0.001		<0.001		<0.001	<0.001
RC								
T1	549 (30)	Ref		Ref		Ref		
T2	628 (34.1)	1.16 (1.03 – 1.30)	0.012	1.14 (1.02 – 1.28)	0.024	1.20 (1.07 – 1.34)	0.002	0.002
T3	796 (43.7)	1.59 (1.43 – 1.77)	<0.001	1.56 (1.40 – 1.74)	<0.001	1.53 (1.37 – 1.71)	<0.001	<0.001
P for trend			<0.001		<0.001		<0.001	<0.001
RC/HDL-C								
T1	562 (30.6)	Ref		Ref		Ref		
T2	613 (33.5)	1.11 (0.99 – 1.24)	0.082	1.10 (0.98 – 1.23)	0.103	1.11 (0.99 – 1.24)	0.077	0.077
T3	798 (43.6)	1.55 (1.39 – 1.73)	<0.001	1.54 (1.38 – 1.72)	<0.001	1.53 (1.37 – 1.71)	<0.001	<0.001
P for trend			<0.001		<0.001		<0.001	<0.001

Model 1: adjusted for age and gender.

Model 2: adjusted for Model 1 + ethnicity, marital status, educational level, residential location, employment status, medical insurance coverage, smoking and drinking history, sleep duration, life satisfaction.

AC – atherogenic coefficient, AIP – atherogenic index of plasma, LCI – lipoprotein combine index, Non-HDL-C – non-high-density lipoprotein cholesterol, CRI-I – Castelli risk index-I, CRI-II – Castelli risk index-II, RC – remnant cholesterol, RC/HDL-C – remnant cholesterol/high density lipoprotein cholesterol, CRP – C-reactive protein.

Table S15. Associations between non-conventional lipid and lipid-inflammatory parameters and hypertension risk after incorporation of blood indicators

Variable	Event, n(%)	Crude Model		Model 1		Model 2		
		HR (95%CI)	*P* value	HR (95%CI)	*P* value	HR (95%CI)	*P* value	Adjusted *P*
AC-CRP								
T1	706 (30.7)	Ref		Ref		Ref		
T2	835 (36.4)	1.22 (1.11 – 1.35)	<0.001	1.19 (1.08 – 1.32)	0.001	1.15 (1.04 – 1.28)	0.006	0.006
T3	991 (43.1)	1.53 (1.39 – 1.68)	<0.001	1.47 (1.33 – 1.62)	<0.001	1.36 (1.23 – 1.51)	<0.001	<0.001
P for trend			<0.001		<0.001		<0.001	<0.001
AIP-CRP								
T1	721 (31.3)	Ref		Ref		Ref		
T2	788 (34.4)	1.11 (1.00 – 1.23)	0.041	1.12 (1.01 – 1.24)	0.028	1.12 (1.01 – 1.24)	0.036	0.036
T3	1023 (44.5)	1.56 (1.41 – 1.71)	<0.001	1.54 (1.40 – 1.70)	<0.001	1.45 (1.31 – 1.61)	<0.001	<0.001
P for trend			<0.001		<0.001		<0.001	<0.001
CRI-I-CRP								
T1	706 (30.7)	Ref		Ref		Ref		
T2	841 (36.6)	1.23 (1.11 – 1.36)	<0.001	1.20 (1.09 – 1.33)	<0.001	1.16 (1.05 – 1.28)	0.005	0.005
T3	985 (42.9)	1.52 (1.38 – 1.67)	<0.001	1.46 (1.32 – 1.61)	<0.001	1.35 (1.22 – 1.49)	<0.001	<0.001
P for trend			<0.001		<0.001		<0.001	<0.001
CRI-II-CRP								
T1	728 (31.7)	Ref		Ref		Ref		
T2	825 (35.9)	1.17 (1.06 – 1.29)	0.003	1.13 (1.02 – 1.25)	0.016	1.10 (0.99 – 1.22)	0.067	0.067
T3	979 (42.6)	1.45 (1.32 – 1.60)	<0.001	1.39 (1.26 – 1.53)	<0.001	1.29 (1.16 – 1.42)	<0.001	<0.001
P for trend			<0.001		<0.001		<0.001	<0.001
LCI-CRP								

T1	699 (30.4)	Ref		Ref		Ref		
T2	807 (35.1)	1.18 (1.07 – 1.31)	0.001	1.15 (1.04 – 1.27)	0.008	1.12 (1.01 – 1.25)	0.025	0.025
T3	1026 (44.7)	1.61 (1.46 – 1.77)	<0.001	1.56 (1.42 – 1.72)	<0.001	1.45 (1.31 – 1.61)	<0.001	<0.001
P for trend			<0.001		<0.001		<0.001	<0.001
Non-HDL-C-CRP								
T1	715 (31.1)	Ref		Ref		Ref		
T2	843 (36.7)	1.22 (1.10 – 1.35)	<0.001	1.17 (1.06 – 1.30)	0.002	1.13 (1.02 – 1.25)	0.017	0.017
T3	974 (42.4)	1.47 (1.33 – 1.62)	<0.001	1.39 (1.26 – 1.53)	<0.001	1.28 (1.15 – 1.42)	<0.001	<0.001
P for trend			<0.001		<0.001		<0.001	<0.001
RC-CRP								
T1	697 (30.3)	Ref		Ref		Ref		
T2	826 (36)	1.22 (1.11 – 1.35)	<0.001	1.20 (1.08 – 1.33)	<0.001	1.17 (1.06 – 1.29)	0.003	0.003
T3	1009 (43.9)	1.59 (1.44 – 1.75)	<0.001	1.55 (1.41 – 1.71)	<0.001	1.43 (1.29 – 1.59)	<0.001	<0.001
P for trend			<0.001		<0.001		<0.001	<0.001
RC/HDL-C-CRP								
T1	713 (31)	Ref		Ref		Ref		
T2	817 (35.6)	1.18 (1.07 – 1.30)	0.001	1.17 (1.06 – 1.29)	0.003	1.15 (1.03 – 1.27)	0.009	0.009
T3	1002 (43.6)	1.53 (1.39 – 1.69)	<0.001	1.52 (1.38 – 1.67)	<0.001	1.41 (1.27 – 1.57)	<0.001	<0.001
P for trend			<0.001		<0.001		<0.001	<0.001
AC								
T1	716 (31.2)	Ref		Ref		Ref		
T2	834 (36.3)	1.20 (1.09 – 1.33)	<0.001	1.23 (1.11 – 1.36)	<0.001	1.22 (1.11 – 1.35)	<0.001	<0.001
T3	982 (42.8)	1.49 (1.35 – 1.64)	<0.001	1.52 (1.38 – 1.67)	<0.001	1.43 (1.30 – 1.59)	<0.001	<0.001
P for trend			<0.001		<0.001		<0.001	<0.001
AIP								
T1	728 (31.7)	Ref		Ref		Ref		
T2	797 (34.7)	1.10 (1.00 – 1.22)	0.056	1.13 (1.02 – 1.25)	0.02	1.12 (1.01 – 1.24)	0.032	0.032
T3	1007 (43.8)	1.51 (1.37 – 1.66)	<0.001	1.57 (1.43 – 1.73)	<0.001	1.50 (1.36 – 1.66)	<0.001	<0.001
P for trend			<0.001		<0.001		<0.001	<0.001

CRI-I								
T1	716 (31.2)	Ref		Ref		Ref		
T2	834 (36.3)	1.20 (1.09 – 1.33)	<0.001	1.23 (1.11 – 1.36)	<0.001	1.22 (1.11 – 1.35)	<0.001	<0.001
T3	982 (42.8)	1.49 (1.35 – 1.64)	<0.001	1.52 (1.38 – 1.67)	<0.001	1.43 (1.30 – 1.59)	<0.001	<0.001
P for trend			<0.001		<0.001		<0.001	<0.001
CRI-II								
T1	739 (32.2)	Ref		Ref		Ref		
T2	844 (36.7)	1.17 (1.06 – 1.29)	0.002	1.18 (1.07 – 1.31)	0.001	1.18 (1.07 – 1.30)	0.001	0.001
T3	949 (41.3)	1.37 (1.24 – 1.50)	<0.001	1.37 (1.24 – 1.50)	<0.001	1.30 (1.18 – 1.44)	<0.001	<0.001
P for trend			<0.001		<0.001		<0.001	<0.001
LCI								
T1	716 (31.2)	Ref		Ref		Ref		
T2	804 (35)	1.15 (1.04 – 1.27)	0.008	1.15 (1.04 – 1.28)	0.005	1.14 (1.03 – 1.27)	0.009	0.009
T3	1012 (44.1)	1.55 (1.40 – 1.70)	<0.001	1.58 (1.43 – 1.74)	<0.001	1.48 (1.33 – 1.63)	<0.001	<0.001
P for trend			<0.001		<0.001		<0.001	<0.001
Non-HDL-C								
T1	734 (32.1)	Ref		Ref		Ref		
T2	836 (36.3)	1.17 (1.06 – 1.29)	0.002	1.17 (1.06 – 1.29)	0.002	1.14 (1.03 – 1.26)	0.01	0.01
T3	962 (41.9)	1.40 (1.27 – 1.54)	<0.001	1.38 (1.26 – 1.52)	<0.001	1.28 (1.16 – 1.42)	<0.001	<0.001
P for trend			<0.001		<0.001		<0.001	<0.001
RC								
T1	710 (31.6)	Ref		Ref		Ref		
T2	853 (35.9)	1.17 (1.06 – 1.29)	0.002	1.17 (1.06 – 1.29)	0.002	1.14 (1.03 – 1.27)	0.009	0.009
T3	969 (42.8)	1.47 (1.33 – 1.61)	<0.001	1.50 (1.37 – 1.66)	<0.001	1.40 (1.27 – 1.55)	<0.001	<0.001
P for trend			<0.001		<0.001		<0.001	<0.001
RC/HDL-C								
T1	747 (32.5)	Ref		Ref		Ref		
T2	798 (34.7)	1.08 (0.97 – 1.19)	0.145	1.09 (0.99 – 1.21)	0.087	1.08 (0.98 – 1.19)	0.138	0.138
T3	987 (43)	1.42 (1.29 – 1.56)	<0.001	1.47 (1.34 – 1.62)	<0.001	1.39 (1.26 – 1.54)	<0.001	<0.001

P for trend	<0.001	<0.001	<0.001	<0.001

Model 1: adjusted for age and gender.

Model 2: adjusted for Model 1 + ethnicity, marital status, educational level, residential location, employment status, medical insurance coverage, smoking and drinking history, sleep duration, life satisfaction, fasting blood glucose, WBC, PLT, BUN, Scr, HbA1c and UA.

WBC – white blood cell, PLT – platelets, BUN – blood urea nitrogen, Scr – serum creatinine, HbA1c – glycosylated hemoglobin, UA – uric acid, AC – atherogenic coefficient, AIP – atherogenic index of plasma, LCI – lipoprotein combine index, Non-HDL-C – non-high-density lipoprotein cholesterol, CRI-I – Castelli risk index-I, CRI-II – Castelli risk index-II, RC – remnant cholesterol, RC/HDL-C – remnant cholesterol/high density lipoprotein cholesterol, CRP – C-reactive protein.

Table S16. Cox regression analysis adjusted for time-dependent covariates

**Variable**	**Model 2**		**Variable**	**Model 2**	
	**HR (95%CI)**	***P* value**		**HR (95%CI)**	***P* value**
AC-CRP			Cum_AC-CRP		
T1	Ref		T1	Ref	
T2	1.20 (1.08 – 1.34)	<0.001	T2	1.38 (1.18 – 1.62)	<0.001
T3	1.50 (1.35 – 1.67)	<0.001	T3	1.66 (1.43 – 1.94)	<0.001
AIP-CRP			Cum_AIP-CRP		
T1	Ref		T1	Ref	
T2	1.15 (1.03 – 1.28)	0.015	T2	1.35 (1.15 – 1.58)	<0.001
T3	1.60 (1.44 – 1.78)	<0.001	T3	1.82 (1.56 – 2.12)	<0.001
CRI-I-CRP			Cum_CRI-I-CRP		
T1	Ref		T1	Ref	
T2	1.22 (1.09 – 1.36)	<0.001	T2	1.46 (1.24 – 1.70)	<0.001
T3	1.49 (1.34 – 1.66)	<0.001	T3	1.67 (1.43 – 1.95)	<0.001
CRI-II-CRP			Cum_CRI-II-CRP		
T1	Ref		T1	Ref	
T2	1.15 (1.03 – 1.28)	0.012	T2	1.43 (1.22 – 1.67)	<0.001
T3	1.41 (1.27 – 1.56)	<0.001	T3	1.61 (1.38 – 1.88)	<0.001
LCI-CRP			Cum_LCI-CRP		
T1	Ref		T1	Ref	

T2	1.16 (1.04 – 1.29)	0.009	T2	1.33 (1.14 – 1.56)	<0.001
T3	1.63 (1.47 – 1.81)	<0.001	T3	1.85 (1.58 – 2.15)	<0.001
Non-HDL-C-CRP			Cum_Non-HDL-C-CRP		
T1	Ref		T1	Ref	
T2	1.19 (1.07 – 1.33)	0.002	T2	1.35 (1.15 – 1.58)	<0.001
T3	1.42 (1.28 – 1.58)	<0.001	T3	1.61 (1.38 – 1.88)	<0.001
RC-CRP			Cum_RC-CRP		
T1	Ref		T1	Ref	
T2	1.19 (1.07 – 1.33)	0.002	T2	1.34 (1.14 – 1.57)	<0.001
T3	1.57 (1.41 – 1.74)	<0.001	T3	1.69 (1.45 – 1.96)	<0.001
RC/HDL-C-CRP			Cum_RC/HDL-C-CRP		
T1	Ref		T1	Ref	
T2	1.17 (1.05 – 1.31)	0.004	T2	1.45 (1.23 – 1.69)	<0.001
T3	1.54 (1.39 – 1.71)	<0.001	T3	1.77 (1.52 – 2.06)	<0.001

Model 2: adjusted for age, gender, ethnicity, marital status, educational level, residential location, employment status, medical insurance coverage, smoking and drinking history, sleep duration, life satisfaction.

Table S17. Inverse probability-weighted Cox regression analysis of the association between non-conventional lipid-inflammatory parameters and hypertension

**Variable**	**Model 2**		**Variable**	**Model 2**	
	**HR (95%CI)**	***P* value**		**HR (95%CI)**	***P* value**
AC-CRP			Cum_AC-CRP		
T1	Ref		T1	Ref	
T2	1.20 (1.08 – 1.32)	<0.001	T2	1.37 (1.17 – 1.59)	<0.001
T3	1.48 (1.34 – 1.63)	<0.001	T3	1.64 (1.41 – 1.90)	<0.001
AIP-CRP			Cum_AIP-CRP		
T1	Ref		T1	Ref	
T2	1.13 (1.02 – 1.25)	0.021	T2	1.31 (1.12 – 1.52)	<0.001
T3	1.57 (1.42 – 1.73)	<0.001	T3	1.75 (1.51 – 2.03)	<0.001

CRI-I-CRP			Cum_CRI-I-CRP		
T1	Ref		T1	Ref	
T2	1.21 (1.09 – 1.34)	<0.001	T2	1.44 (1.23 – 1.68)	<0.001
T3	1.46 (1.33 – 1.61)	<0.001	T3	1.66 (1.43 – 1.93)	<0.001
CRI-II-CRP			Cum_CRI-II-CRP		
T1	Ref		T1	Ref	
T2	1.14 (1.03 – 1.26)	0.011	T2	1.42 (1.22 – 1.65)	<0.001
T3	1.4 (1.27 – 1.54)	<0.001	T3	1.6 (1.38 – 1.86)	<0.001
LCI-CRP			Cum_LCI-CRP		
T1	Ref		T1		
T2	1.15 (1.04 – 1.28)	0.007	T2	1.32 (1.13 – 1.55)	<0.001
T3	1.57 (1.43 – 1.73)	<0.001	T3	1.82 (1.57 – 2.11)	<0.001
Non-HDL-C-CRP			Cum_Non-HDL-C-CRP		
T1	Ref		T1	Ref	
T2	1.18 (1.06 – 1.30)	0.002	T2	1.33 (1.14 – 1.55)	<0.001
T3	1.39 (1.26 – 1.54)	<0.001	T3	1.59 (1.37 – 1.84)	<0.001
RC-CRP			Cum_RC-CRP		
T1	Ref		T1	Ref	
T2	1.20 (1.08 – 1.32)	<0.001	T2	1.31 (1.13 – 1.53)	<0.001
T3	1.55 (1.41 – 1.71)	<0.001	T3	1.64 (1.42 – 1.90)	<0.001
RC/HDL-C-CRP			Cum_RC/HDL-C-CRP		
T1	Ref		T1	Ref	
T2	1.17 (1.06 – 1.29)	0.003	T2	1.42 (1.22 – 1.65)	<0.001
T3	1.53 (1.39 – 1.69)	<0.001	T3	1.73 (1.49 – 2.00)	<0.001

Model 2: adjusted for age, gender, ethnicity, marital status, educational level, residential location, employment status, medical insurance coverage, smoking and drinking history, sleep duration, life satisfaction.

Table S18. E-value analysis

Variable	E value	Variable	E value	Variable	E value	Variable	E value

AC	2.52	AC-CRP	2.34	Cum_AC	2.73	Cum_AC-CRP	2.73
AIP	2.60	AIP-CRP	2.54	Cum_AIP	2.77	Cum_AIP-CRP	2.98
CRI-I	2.52	CRI-I-CRP	2.32	Cum_CRI-I	2.73	Cum_CRI-I-CRP	2.77
CRI-II	2.17	CRI-II-CRP	2.17	Cum_CRI-II	2.32	Cum_CRI-II-CRP	2.64
LCI	2.60	LCI-CRP	2.56	Cum_LCI	2.79	Cum_LCI-CRP	3.08
Non-HDL-C	2.15	Non-HDL-C-CRP	2.15	Cum_Non-HDL-C	2.28	Cum_Non-HDL-C-CRP	2.60
RC	2.39	RC-CRP	2.49	Cum_RC	2.58	Cum_RC-CRP	2.77
RC/HDL-C	2.37	RC/HDL-C-CRP	2.45	Cum_RC/HDL-C	2.69	Cum_RC/HDL-C-CRP	2.94

AC – atherogenic coefficient, AIP – atherogenic index of plasma, LCI – lipoprotein combine index, Non-HDL-C – non-high-density lipoprotein cholesterol, CRI-I – Castelli risk index-I, CRI-II – Castelli risk index-II, RC – remnant cholesterol, RC/HDL-C – remnant cholesterol/high density lipoprotein cholesterol, CRP – C-reactive protein.

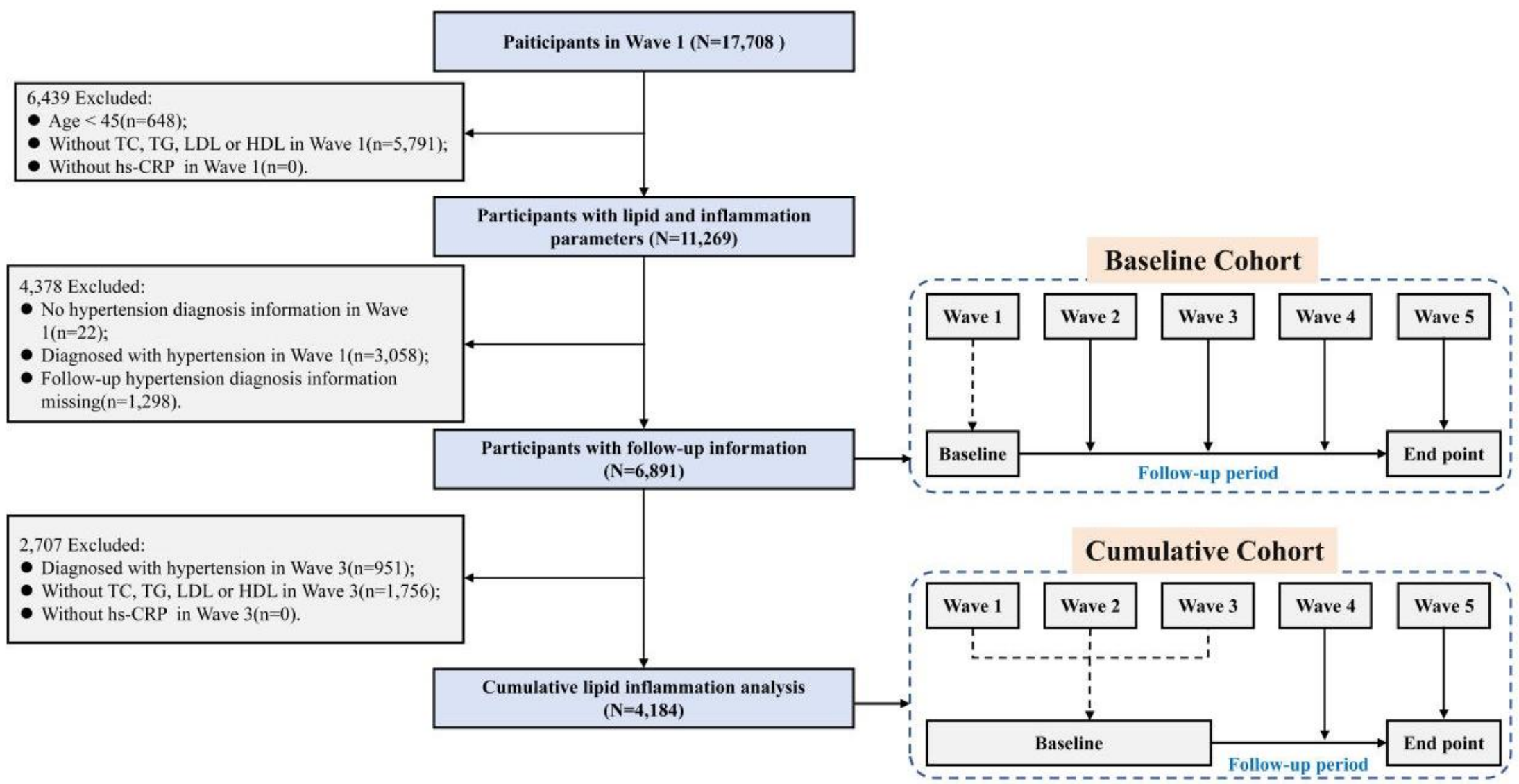

Figure S1. Flowchart of participant selection

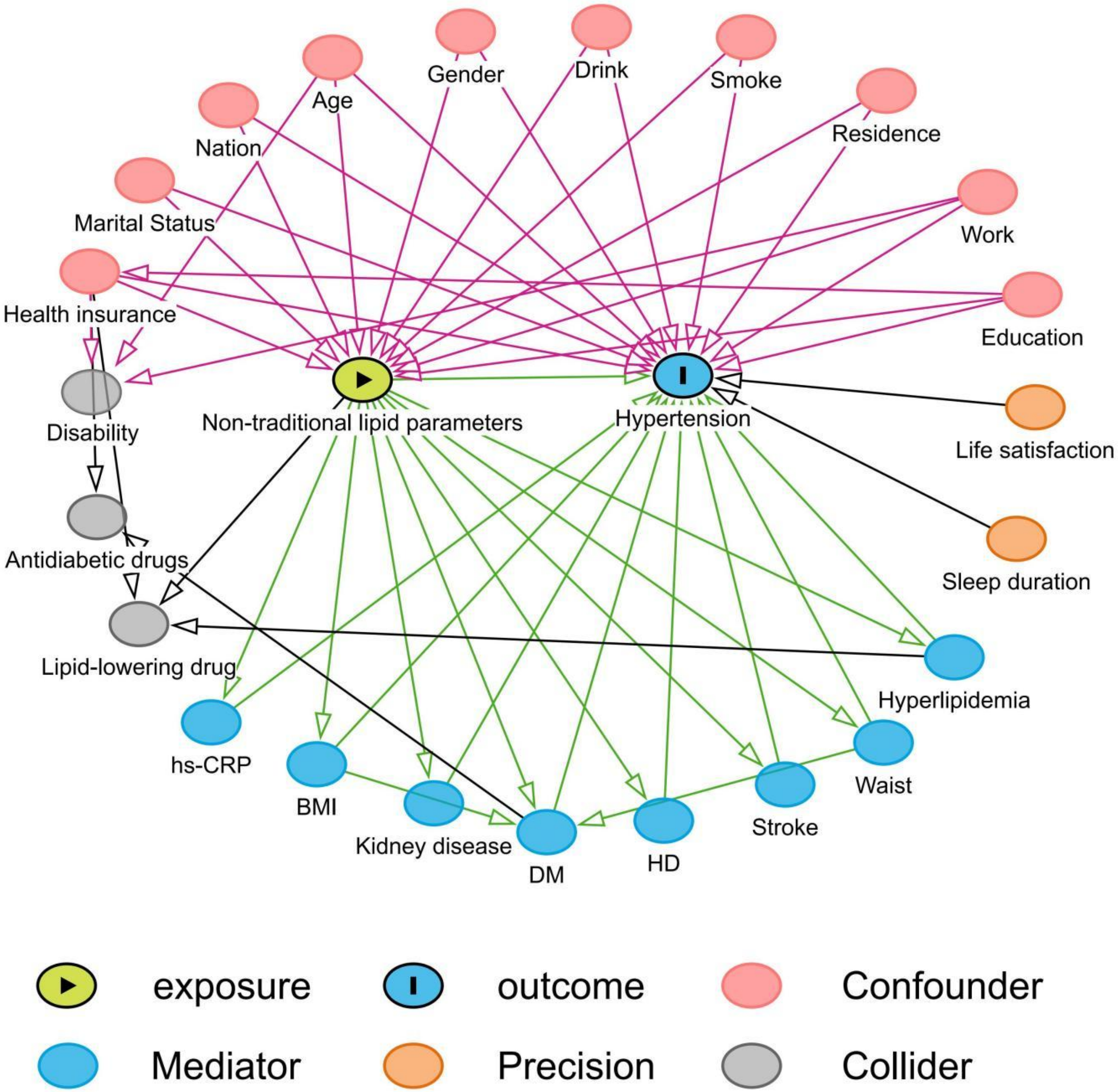

Figure S2. Directed acyclic graph illustrating the associations among exposure factors (non-conventional blood lipid parameters) – outcome indicator (hypertension) and covariates

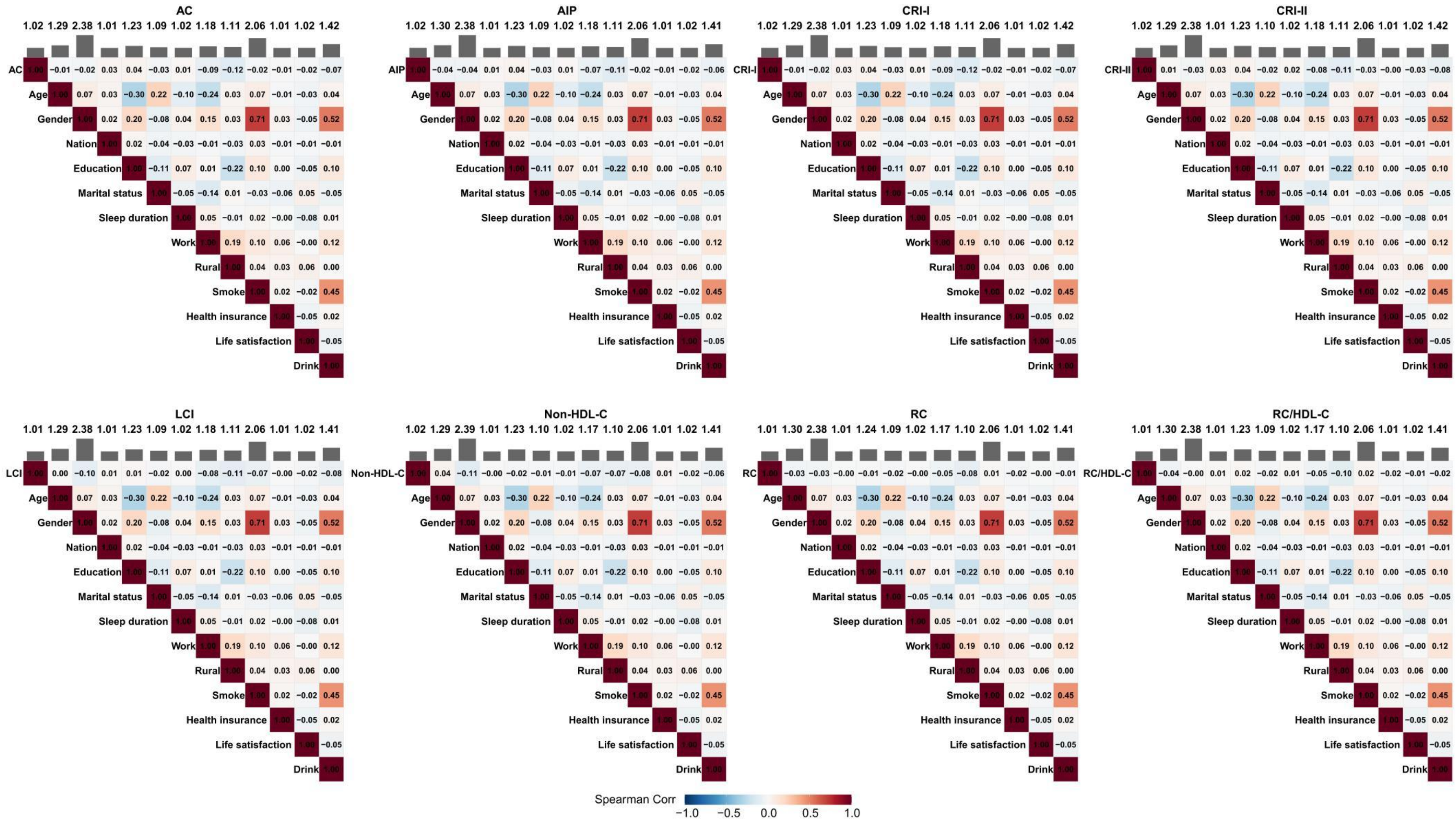

Figure S3. Pearson correlation analysis and VIF assessment of non-conventional lipid parameters and covariates in the model. **Panel A.** AC. **Panel B.** AIP. **Panel C.** CRI-I. **Panel D.** CRI-II. **Panel E.** LCI. **Panel F.** Non-HDL-C. **Panel G.** RC. **Panel H.** RC/HDL-C. VIF – variance inflation factors, AC – atherogenic coefficient, AIP – atherogenic index of plasma, LCI – lipoprotein combine index, Non-HDL-C – non-high-density lipoprotein cholesterol, CRI-I – Castelli risk index-I, CRI-II – Castelli risk index-II, RC – remnant cholesterol, RC/HDL-C –

remnant cholesterol/high density lipoprotein cholesterol.

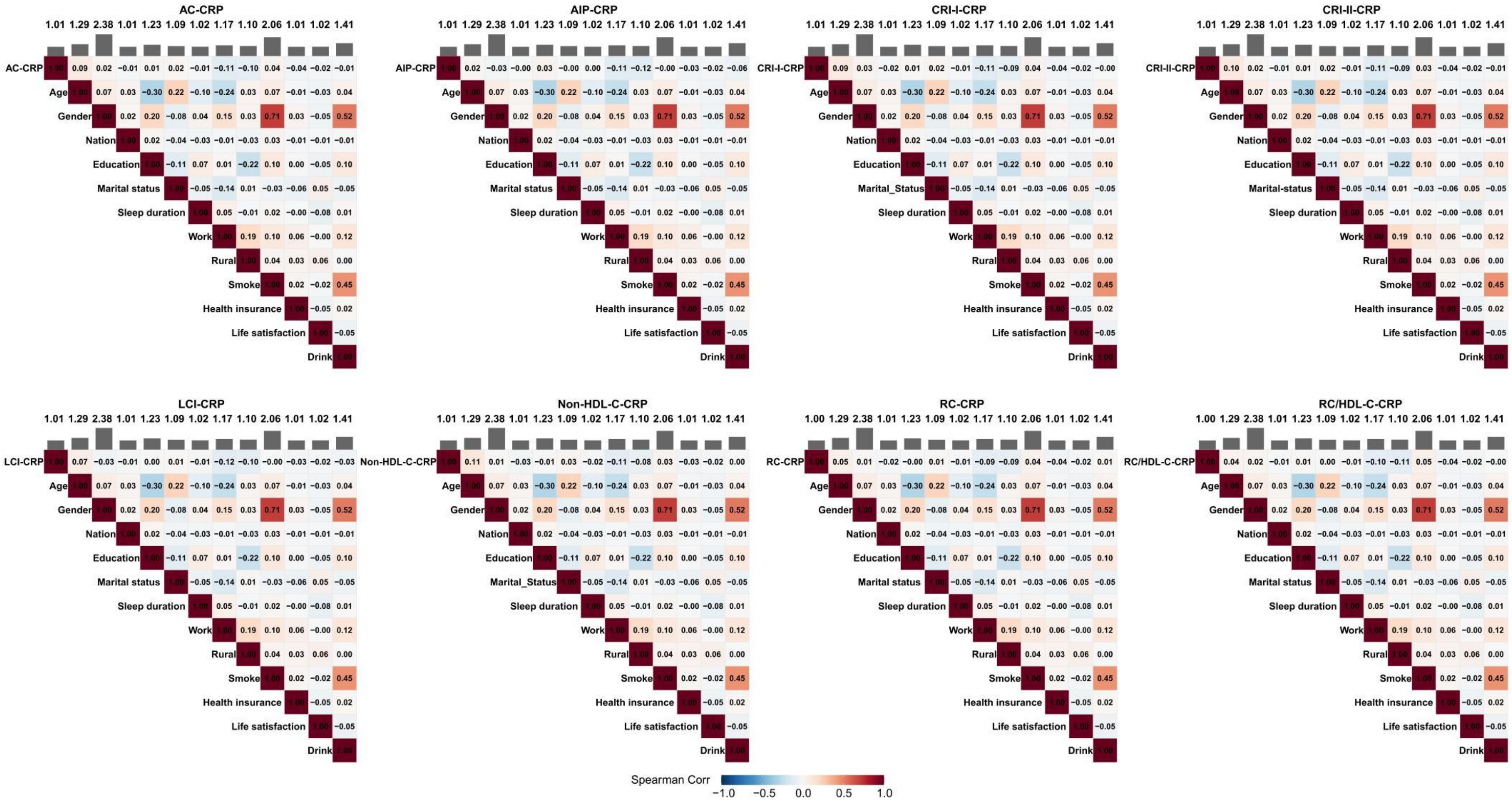

Figure S4. Pearson correlation analysis and VIF assessment of non-conventional lipid-inflammatory parameters and covariates in the model. **Panel A.** AC-CRP. **Panel B.** AIP-CRP. **Panel C.** CRI-I-CRP. **Panel D.** CRI-II-CRP. **Panel E.** LCI-CRP. **Panel F.** Non-HDL-C-CRP. **Panel G.** RC-CRP. **Panel H.** RC/HDL-C-CRP. VIF – variance inflation factors, AC – atherogenic coefficient, AIP – atherogenic index of plasma, LCI – lipoprotein combine index, Non-HDL-C – non-high-density lipoprotein cholesterol, CRI-I – Castelli risk index-I, CRI-II – Castelli risk index-II,

RC – remnant cholesterol, RC/HDL-C – remnant cholesterol/high density lipoprotein cholesterol, CRP – C-reactive protein.

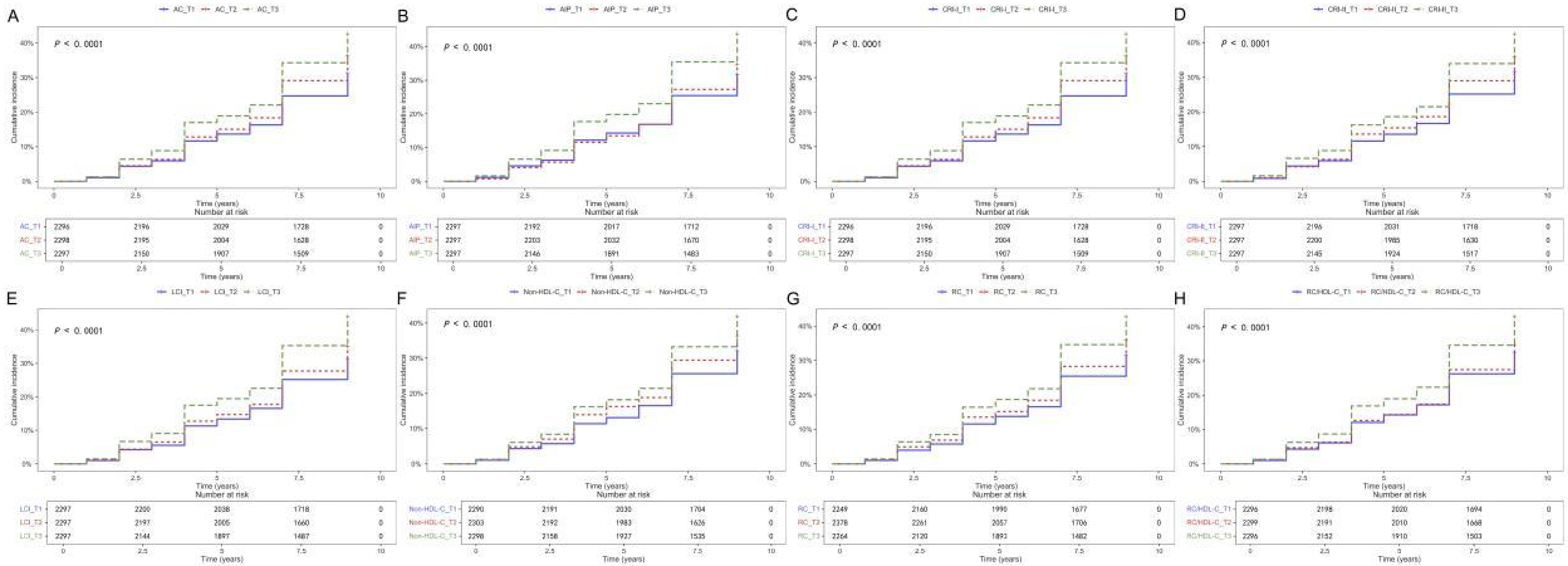

Figure S5. Kaplan-Meier plot of cumulative incidence of hypertension risk based on non-conventional lipid parameter levels. **Panel A.** AC. **Panel B.** AIP. **Panel C.** CRI-I. **Panel D.** CRI-II. **Panel E.** LCI. **Panel F.** Non-HDL-C. **Panel G.** RC. **Panel H.** RC/HDL-C. AC – atherogenic coefficient, AIP – atherogenic index of plasma, LCI – lipoprotein combine index, Non-HDL-C – non-high-density lipoprotein cholesterol, CRI-I – Castelli risk index-I, CRI-II – Castelli risk index-II, RC – remnant cholesterol, RC/HDL-C – remnant cholesterol/high density lipoprotein cholesterol.

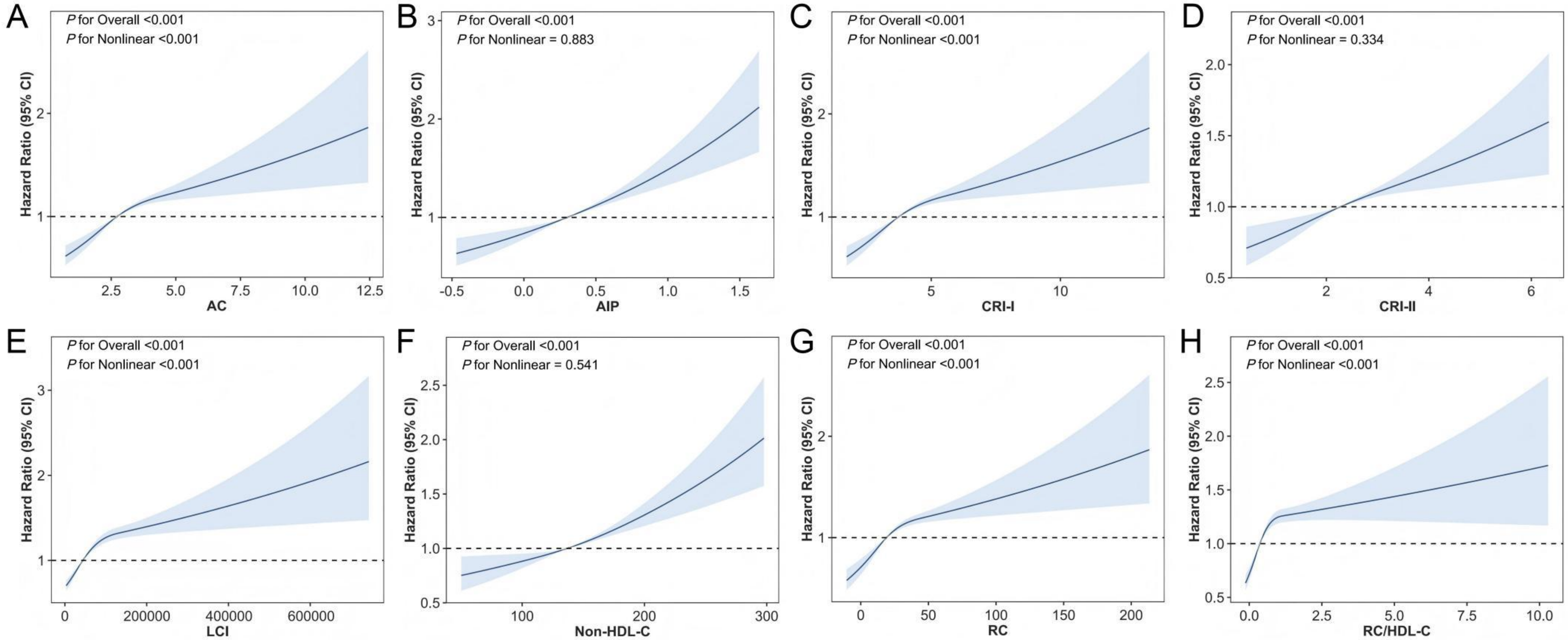

Figure S6. Dose-response relationship between non-conventional lipid parameters and hypertension risk. **Panel A.** AC. **Panel B.** AIP. **Panel C.** CRI-I. **Panel D.** CRI-II. **Panel E.** LCI. **Panel F.** Non-HDL-C. **Panel G.** RC. **Panel H.** RC/HDL-C. AC – atherogenic coefficient, AIP – atherogenic index of plasma, LCI – lipoprotein combine index, Non-HDL-C – non-high-density lipoprotein cholesterol, CRI-I – Castelli risk index-I, CRI-II – Castelli risk index-II, RC – remnant cholesterol, RC/HDL-C – remnant cholesterol/high density lipoprotein cholesterol.

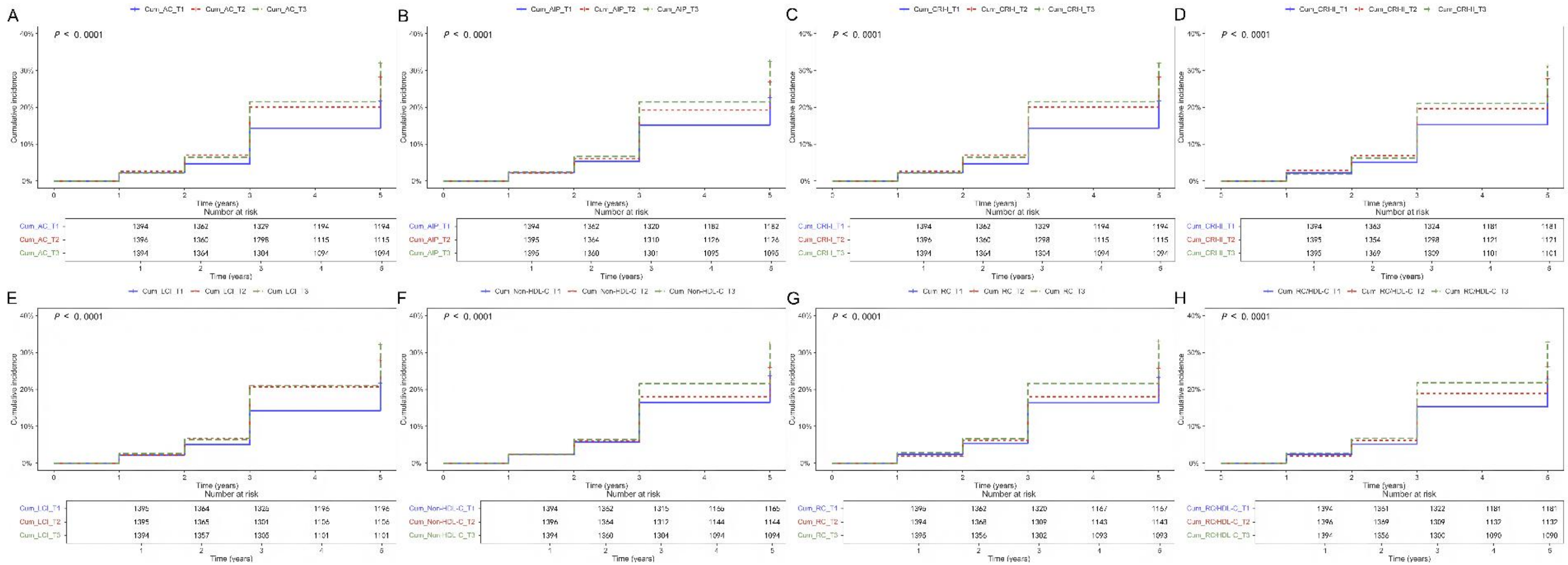

Figure S7. Kaplan-Meier plot of cumulative incidence of hypertension risk based on cumulative non-conventional lipid parameter levels. **Panel A.** Cum_AC. **Panel B.** Cum_AIP. **Panel C.** Cum_CRI-I. **Panel D.** Cum_CRI-II. **Panel E.** Cum_LCI. **Panel F.** Cum_Non-HDL-C. **Panel G.** Cum_RC. **Panel H.** Cum_RC/HDL-C. Cum_AC – cumulative atherogenic coefficient, Cum_AIP – cumulative atherogenic index of plasma, Cum_LCI – cumulative lipoprotein combine index, Cum_Non-HDL-C – cumulative non-high-density lipoprotein cholesterol, Cum_CRI-I – cumulative Castelli risk index-I, Cum_CRI-II – cumulative Castelli risk index-II, Cum_RC – cumulative remnant cholesterol, Cum_RC/HDL-C – cumulative remnant cholesterol/high density lipoprotein cholesterol.

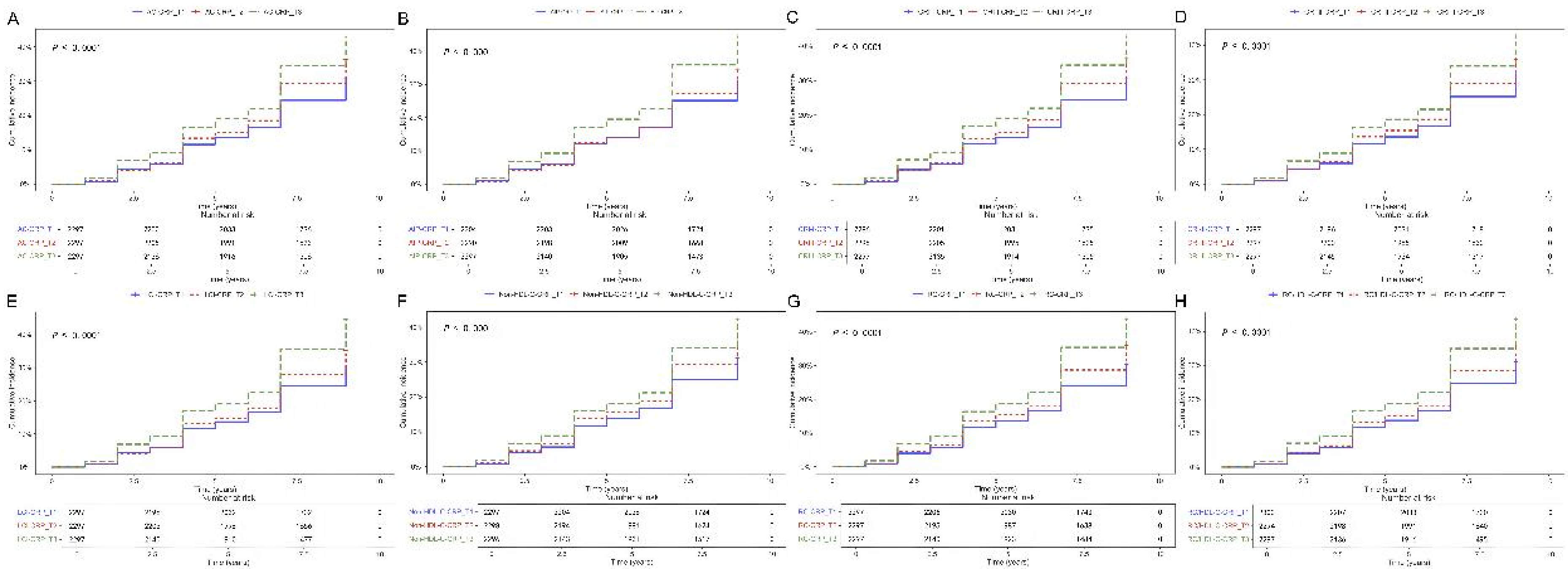

Figure S8. Kaplan-Meier plot of cumulative incidence of hypertension risk based on non-conventional lipid-inflammatory parameter levels. **Panel A.** AC-CRP. **Panel B.** AIP-CRP. **Panel C.** CRI-I-CRP. **Panel D.** CRI-II-CRP. **Panel E.** LCI-CRP. **Panel F.** Non-HDL-C-CRP. **Panel G.** RC-CRP. **Panel H.** RC/HDL-C-CRP. AC – atherogenic coefficient, AIP – atherogenic index of plasma, LCI – lipoprotein combine index, Non-HDL-C – non-high-density lipoprotein cholesterol, CRI-I – Castelli risk index-I, CRI-II – Castelli risk index-II, RC – remnant cholesterol, RC/HDL-C – remnant cholesterol/high density lipoprotein cholesterol, CRP – C-reactive protein.

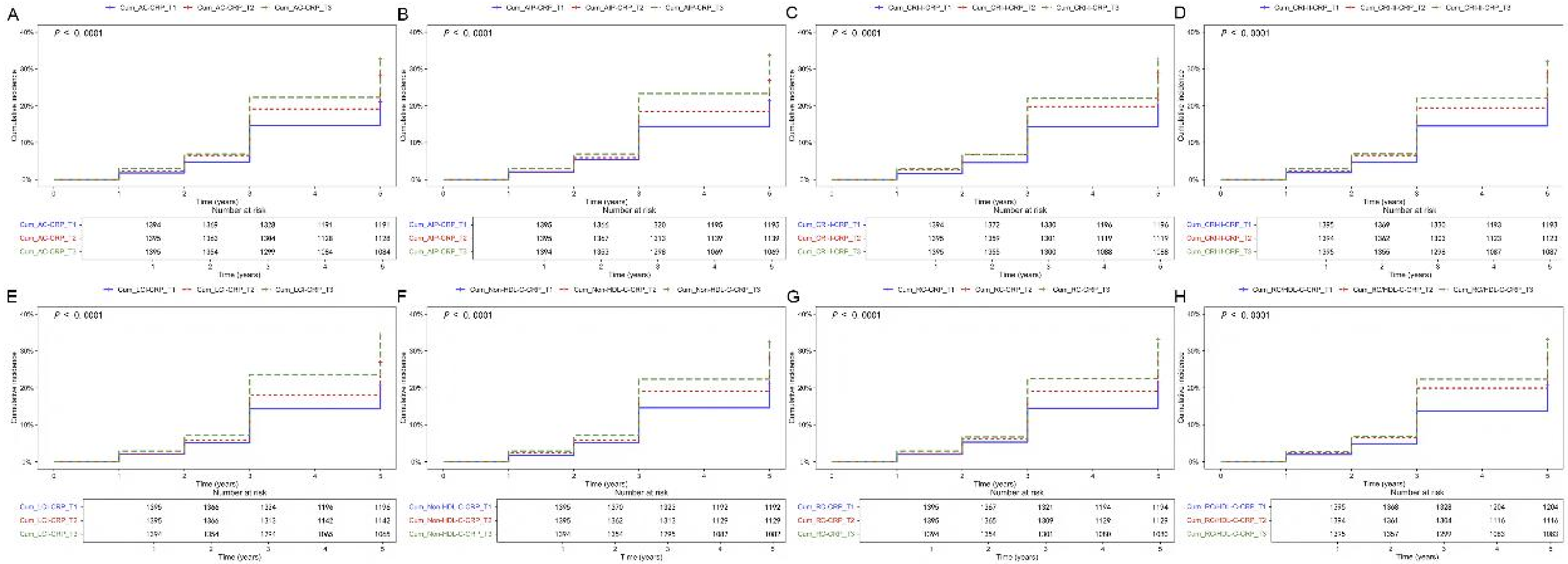

Figure S9. Kaplan-Meier plot of cumulative incidence of hypertension risk based on cumulative non-conventional lipid-inflammatory parameter levels. **Panel A.** Cum_AC-CRP. **Panel B.** Cum_AIP-CRP. **Panel C.** Cum_CRI-I-CRP. **Panel D.** Cum_CRI-II-CRP. **Panel E.** Cum_LCI-CRP. **Panel F.** Cum_Non-HDL-C-CRP. **Panel G.** Cum_RC-CRP. **Panel H.** Cum_RC/HDL-C-CRP. Cum_AC – cumulative atherogenic coefficient, Cum_AIP – cumulative atherogenic index of plasma, Cum_LCI – cumulative lipoprotein combine index, Cum_Non-HDL-C – cumulative non-high-density lipoprotein cholesterol, Cum_CRI-I – cumulative Castelli risk index-I, Cum_CRI-II – cumulative Castelli risk index-II, Cum_RC – cumulative remnant cholesterol, Cum_RC/HDL-C – cumulative remnant cholesterol/high density lipoprotein cholesterol, CRP – C-reactive protein.

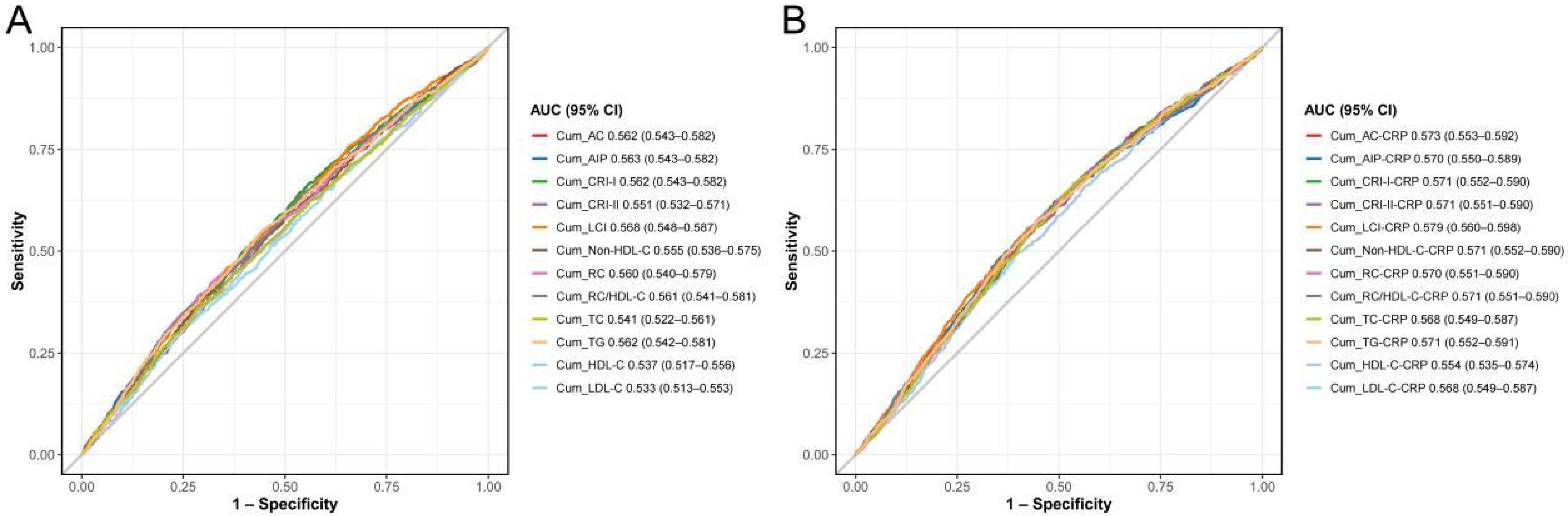

Figure S10. Predictive ability of cumulative lipid and lipid-inflammatory parameters for hypertension. AUC – area under the curve, Cum_AC – cumulative atherogenic coefficient, Cum_AIP – cumulative atherogenic index of plasma, Cum_LCI – cumulative lipoprotein combine index, Cum_Non-HDL-C – cumulative non-high-density lipoprotein cholesterol, Cum_CRI-I – cumulative Castelli risk index-I, Cum_CRI-II – cumulative Castelli risk index-II, Cum_RC – cumulative remnant cholesterol, Cum_RC/HDL-C – cumulative remnant cholesterol/high density lipoprotein cholesterol, CRP – C-reactive protein.

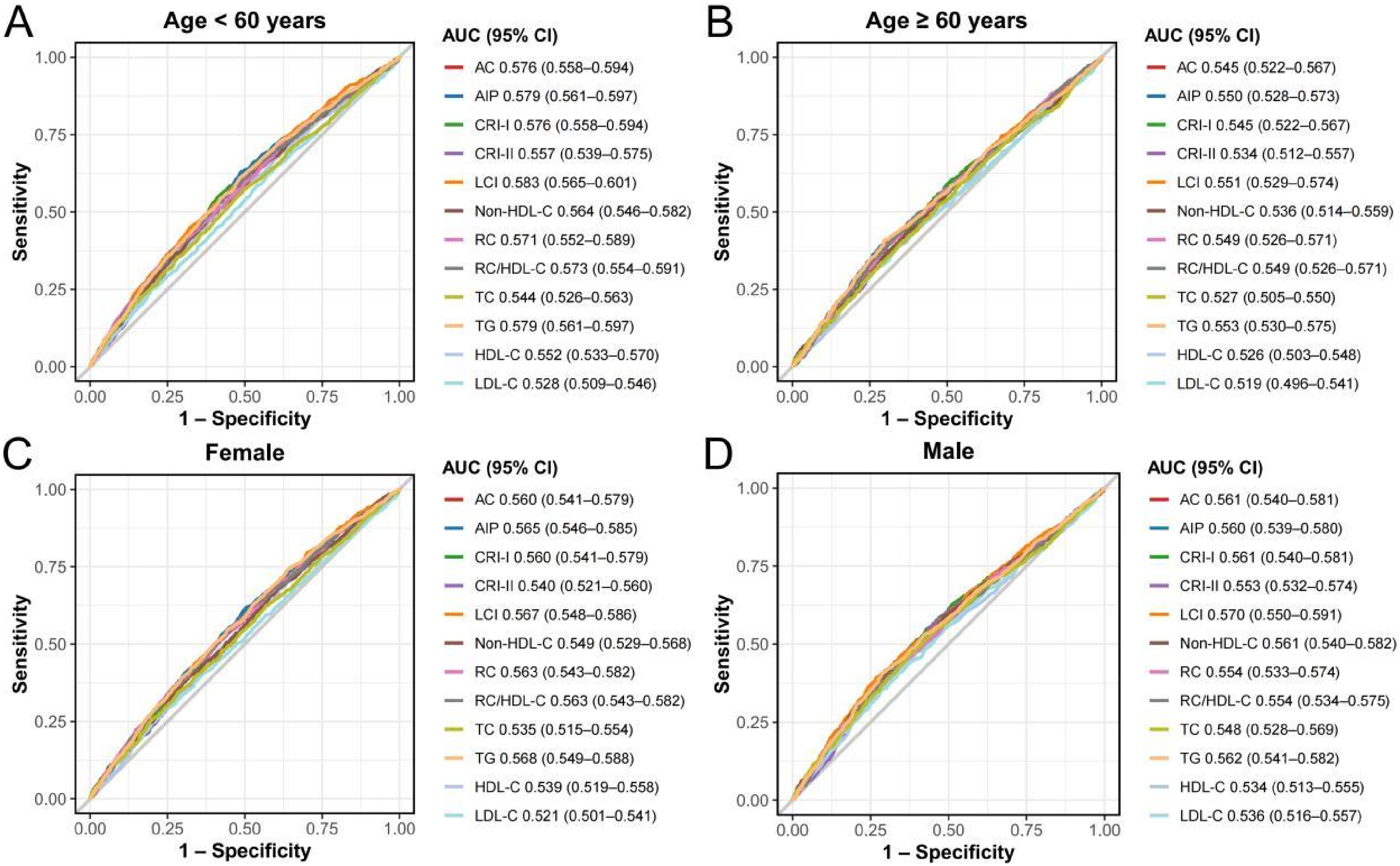

Figure S11. Predictive ability of lipid parameters stratified by age and sex for hypertension. AUC – area under the curve, AC – atherogenic coefficient, AIP – atherogenic index of plasma, LCI – lipoprotein combine index, Non-HDL-C – non-high-density lipoprotein cholesterol, CRI-I – Castelli risk index-I, CRI-II – Castelli risk index-II, RC – remnant cholesterol, RC/HDL-C – remnant cholesterol/high density lipoprotein cholesterol.

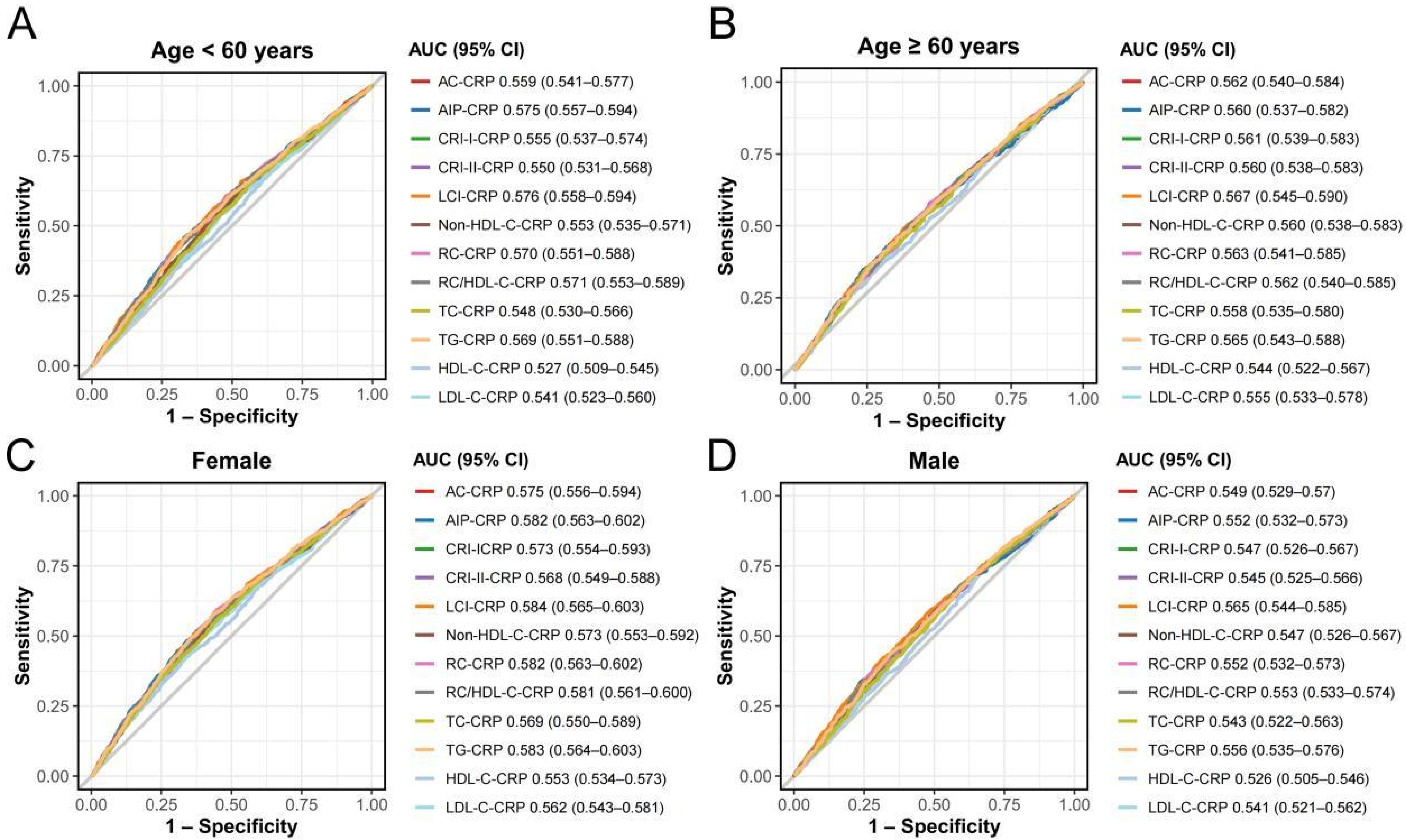

Figure S12. Predictive ability of lipid-inflammatory parameters stratified by age and sex for hypertension. AUC – area under the curve, AC – atherogenic coefficient, AIP – atherogenic index of plasma, LCI – lipoprotein combine index, Non-HDL-C – non-high-density lipoprotein cholesterol, CRI-I – Castelli risk index-I, CRI-II – Castelli risk index-II, RC – remnant cholesterol, RC/HDL-C – remnant cholesterol/high density lipoprotein cholesterol, CRP – C-reactive protein.

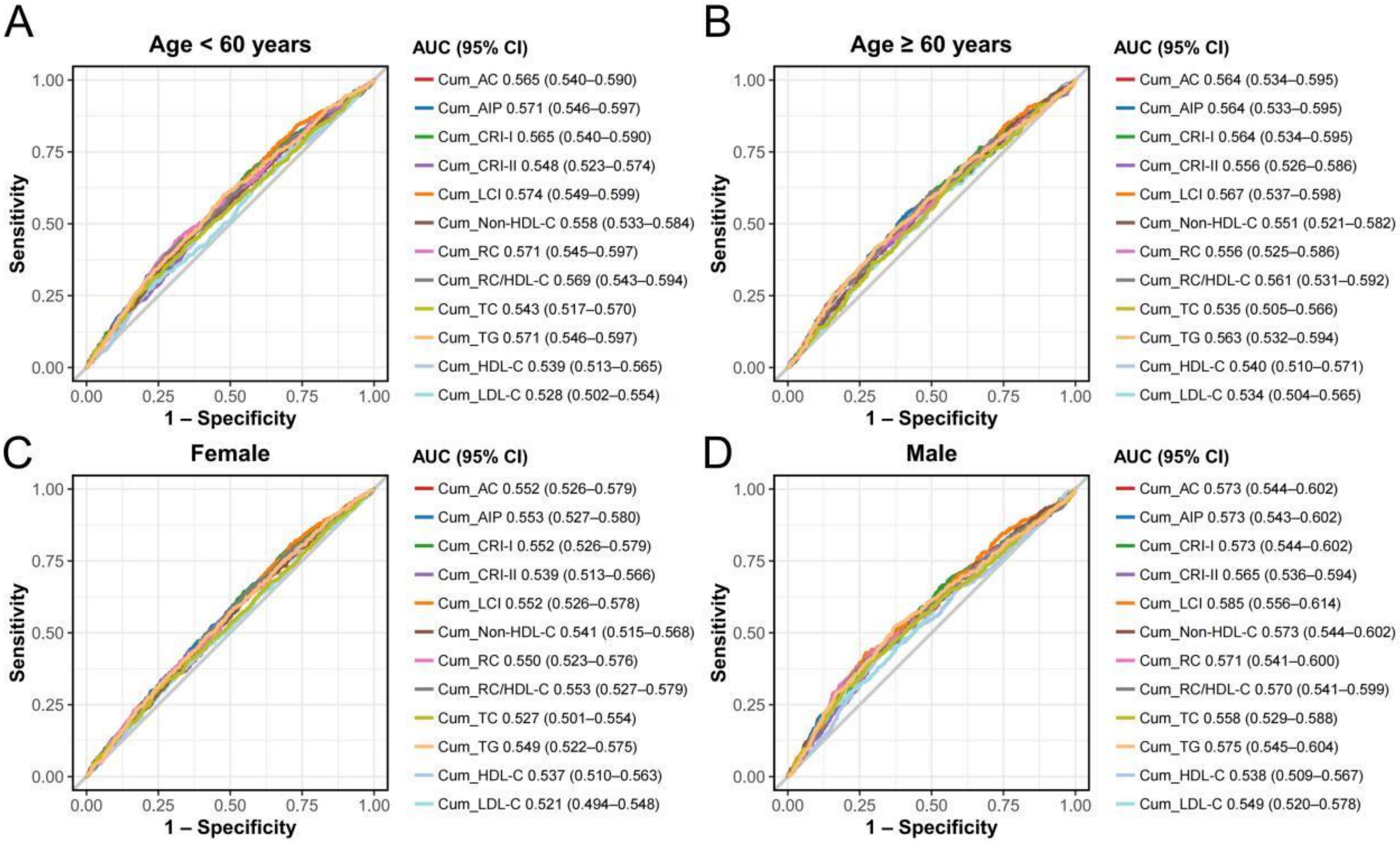

Figure S13. Predictive ability of cumulative lipid parameters stratified by age and sex for hypertension. AUC – area under the curve, Cum_AC – cumulative atherogenic coefficient, Cum_AIP – cumulative atherogenic index of plasma, Cum_LCI – cumulative lipoprotein combine index, Cum_Non-HDL-C – cumulative non-high-density lipoprotein cholesterol, Cum_CRI-I – cumulative Castelli risk index-I, Cum_CRI-II – cumulative Castelli risk index-II, Cum_RC – cumulative remnant cholesterol, Cum_RC/HDL-C – cumulative remnant cholesterol/high density lipoprotein cholesterol.

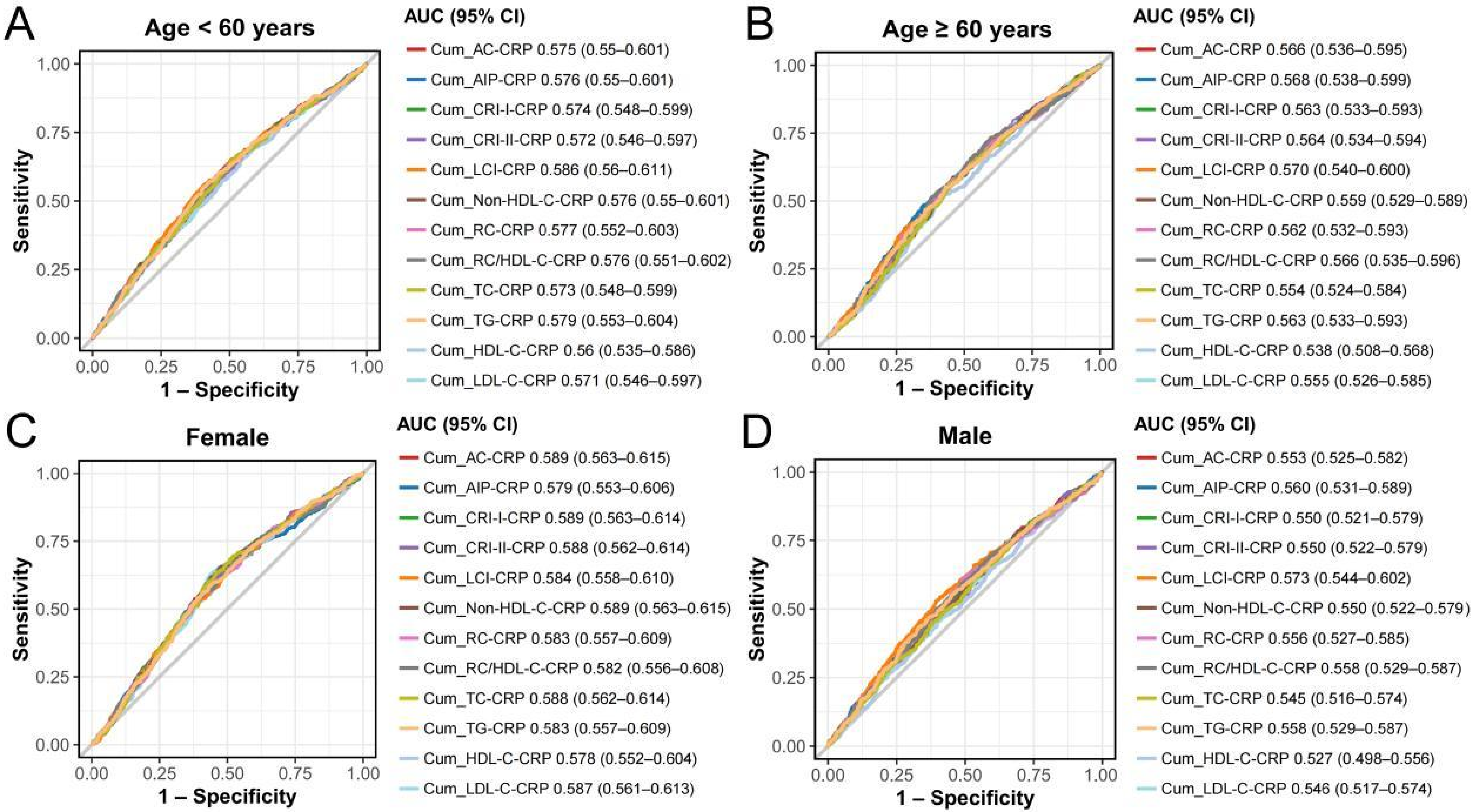

Figure S14. Predictive ability of cumulative lipid-inflammatory parameters stratified by age and sex for hypertension. AUC – area under the curve, Cum_AC – cumulative atherogenic coefficient, Cum_AIP – cumulative atherogenic index of plasma, Cum_LCI – cumulative lipoprotein combine index, Cum_Non-HDL-C – cumulative non-high-density lipoprotein cholesterol, Cum_CRI-I – cumulative Castelli risk index-I, Cum_CRI-II – cumulative Castelli risk index-II, Cum_RC – cumulative remnant cholesterol, Cum_RC/HDL-C – cumulative remnant cholesterol/high density lipoprotein cholesterol, CRP – C-reactive protein.
